# New Chemical Constituents from the Bark of *Dendropanax morbifera* Leveille and Their Evaluation of Antioxidant Activities

**DOI:** 10.3390/molecules24213967

**Published:** 2019-11-01

**Authors:** Ill-Min Chung, Seung-Hyun Kim, Chang Kwon, So-Yeon Kim, Yu-Jin Yang, Ju-Sung Kim, Mohd Ali, Ateeque Ahmad

**Affiliations:** 1 Department of Crop Science, College of Sanghuh Life Science, Konkuk University, 120 Neungdong-ro, Gwangjin-gu, Seoul 05029, Korea; imcim@konkuk.ac.kr (I.-M.C.); kshkim@konkuk.ac.kr (S.-H.K.); chang794@konkuk.ac.kr (C.W.); hellosy1@konkuk.ac.kr (S.-Y.K.); jin0931@konkuk.ac.kr (Y.-J.Y.); 2 Major in Plant Resource and Environment, SARI, Jeju National University, Jeju 63243, Korea; aha2011@jejunu.ac.kr; 3 Department of Pharmacognosy and Phytochemistry, Hamdard University, New Delhi 110062, India; maliphyto@gmail.com; 4 Process Chemistry and Technology Department, CSIR-Central Institute of Medicinal and Aromatic Plants, Lucknow 226015, India

**Keywords:** structure determination, new and known compounds, DPPH, NO, reducing power phosphomolybdenum activities

## Abstract

Four new constituents, as *cis*-6-oxogeran-4-enyl-10-oxy-*O*-β-arabinopyranosyl-4′-*O*-β-arabinopyranosyl-2″-octadec-9‴,12‴,15‴-trienoate (**1**), geran-3(10)-enyl-1-oxy-*O*-β-arabinopyranosyl-4′-*O*-β-arabinopyranosyl-2″-octadec-9‴,12‴,15‴-trienoate (**2**), geranilan-8-oxy-*O*-*α*-d-xylopyranosyl-2′-*n*-octadec-9″,12″,15″-trienoate (**3**), 1-cyclohex-2′, 5′-dienyl 1-cyclohexylethanol-*O*-β-d-xylopyranoside (**4**), along with six known constituents, guaiacol-*O*-β-d-arabinopyaranoside (**5**), *n*-tetradecanyl oleate (**6**), oleyl-*O-*β-d-xyloside (**7**), *n*-octadec-9,12-dienoyl-*O*-β-d-arabinopyranoside (**8**), linolenyl-*O*-β-d-arabinofuranoside (**9**) andglyceryl-1,3-dipalmito-2-olein (**10**), were isolated and identified from the *Dendropanax morbifera* bark. The new structures were established by one-and two-dimensional NMR (and in combination with IR, FAB-MSand HR-ESI-FTMS. The comparative evaluation of antioxidant potential by phosphomolybdenum, DPPH, FRAP and the NO assay of four different compounds (**1**–**4**), we have found that the compounds 1 and 2 have power as a natural antioxidant, whereas the compound **3** and **4** exhibited mild activity in comparison to compounds **1** and **2**.

## 1. Introduction

The genus *Dendropanax* belongs to the family Araliaceae, can be found distributed in East Asia, Korea, Japan, the Malay Peninsula, and central South America. *Dendropanax morbifera* Leveille (Araliaceae) is an endemic species in Korea, and distributes within the southern part of Korea [1]. The roots and stems of this plant are used in folk medicine for the treatment of migraine headaches, dysmenorrhea and to remove wind dampness [2,3]. The plant is commonly cultivated in gardens, and it is sometimes used for flower arrangements. The stem is erect and, can grow to a height of 5 m. The leaves are 3-lobed and glossy green in color.

**Figure 1:**
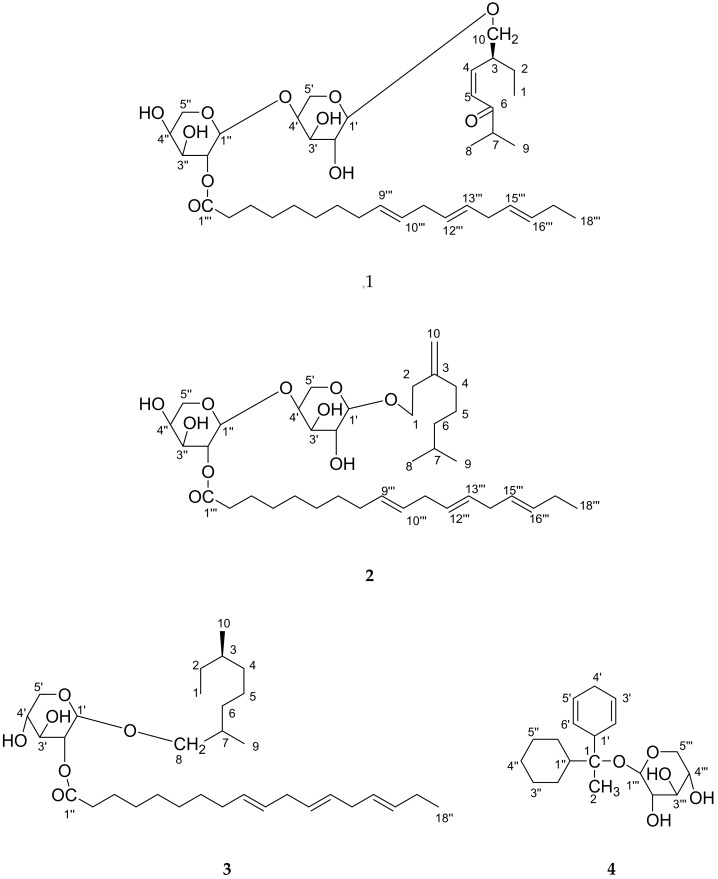
New chemical structures (**1**–**4**) isolated from *D. morbifera*.

**Figure 2:**
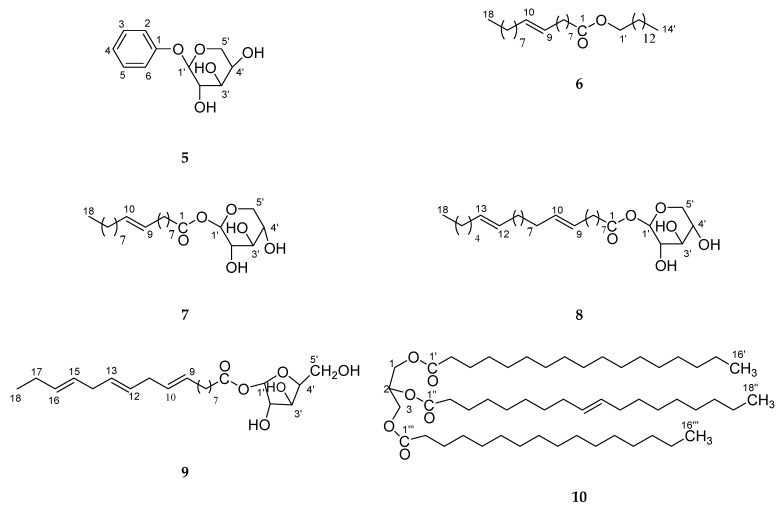
Known chemical structures (**5**–**10**) isolated from *D. morbifera*.

Polyactylenes and falcarinol compounds have been isolated from *Dendropanx arboreus* and shown to have cytotoxic activity [4,5], antiseptic effects [6] and to be major allergens [7]. Two antifungal falcarinols were described [6] and several other acetylinic constituents, such as *cis*-9, 17-octadecadiene-12, 14-diyne-1, 16-diol, 16-hydroxy-*cis*-9,17-octadecadiene-12,14-diynoic acid, and *cis*-9, trans-16-octadecadiene-12,14-diynoic acid from *D. trifidus* have been reported [7]. Although a diverse selection of polyacetylenes has been isolated from this family, biological evaluations of these compounds with the exception of falcarinol, have been preliminary in nature [5,8]. Dendrotrifidic acid was also isolated from the leaves of *Dendropanax trifidius* [9], and this tree was reported to have antifungal activity. A triterpene oxide, dendropanaxide, also known as epoxyglutinane and campanulin [10], and glutinol showed in vitro cytotoxic activity against P-388 and KB cells [11]. As a part of our continuing research [12,13] to find novel compounds from natural plants, *D. morbifera* [3,14,15,16,17]. In Korea *D. morbifera* Leveille has been long used as a traditional medicine and healthy food. It is recommended for several diseases in original texts of Donguibogam (written in the 17th century, Korean). *D. moribifera* extracts have a history of use in traditional medicine for the treatment of various diseases [17]. Nonetheless, there is no information on the antioxidant effect of thebark of *D. morbifera*, and because of this reason, we have studied the acetone extract from the bark of *D. morbifera*. Herein, the isolation and structure elucidation of four new monoterpenes sugar derivatives of long-chain compounds, *cis*-6-oxogeran-4-enyl-10-oxy-*O*-β-arabinopyranosyl-4′-*O*-β-arabinopyranosyl-2″-octadec-9‴,12‴,15‴-trienoate (**1**), geran-3(10)-enyl-1-oxy-*O*-β-arabinopyranosyl-4′-*O*-β-arabinopyranosyl-2″-octadec-9‴,12‴,15‴-trienoate (**2**), geranilan-8-oxy-*O*-*α*-d-xylopyranosyl-2′-*n*-octadec-9″,12″,15″-trienoate (**3**), and 1-cyclohex-2′,5′-dienyl 1-cyclohexylethanol-*O*-β-d-xylopyranoside **(4)**, along with six known constituents, asguaiacol-*O*-β-d-arabinopyaranoside (**5**), *n*-tetradecanyl oleate (**6**), oleyl-*O*-β-d-xyloside (**7**), *n*-octadec-9,12-dienoyl-*O*-β-d-arabinopyranoside (**8**), linolenyl-*O*-β-d-arabinofuranoside (**9**), andglyceryl-1, 3-dipalmito-2-olein (**10**), were isolated and identified from *D. morbifera* bark. The new structures (**1**–**4**; Figure 1) were established by one and two-dimensional NMR (Nuclear Magnetic Resonance), and in combination with IR, FABMS (Fast atom bombardment-mass spectrometry) and HR-ESTFTMS, (High resolution electrospray ionization spectroscopy-Fourier Transform mass spectrometry) along with six known compounds (**5–10**; Figure 2) for the first time in this plant. The compounds **1**–**4** were investigated for the antioxidant potential using 1,1-diphenyl-2-picrylhydrazyl (DPPH) radical scavenging activity, reducing power and nitric oxide (NO) and phosphomolybdenum activity, and the results demonstrate that the compounds **1** and **2** have power as a natural antioxidant, whereas the compounds **3** and **4** exhibited mild activity in comparison to compounds **1** and **2**.

## 2. Results and Discussion

**Figure 3:**
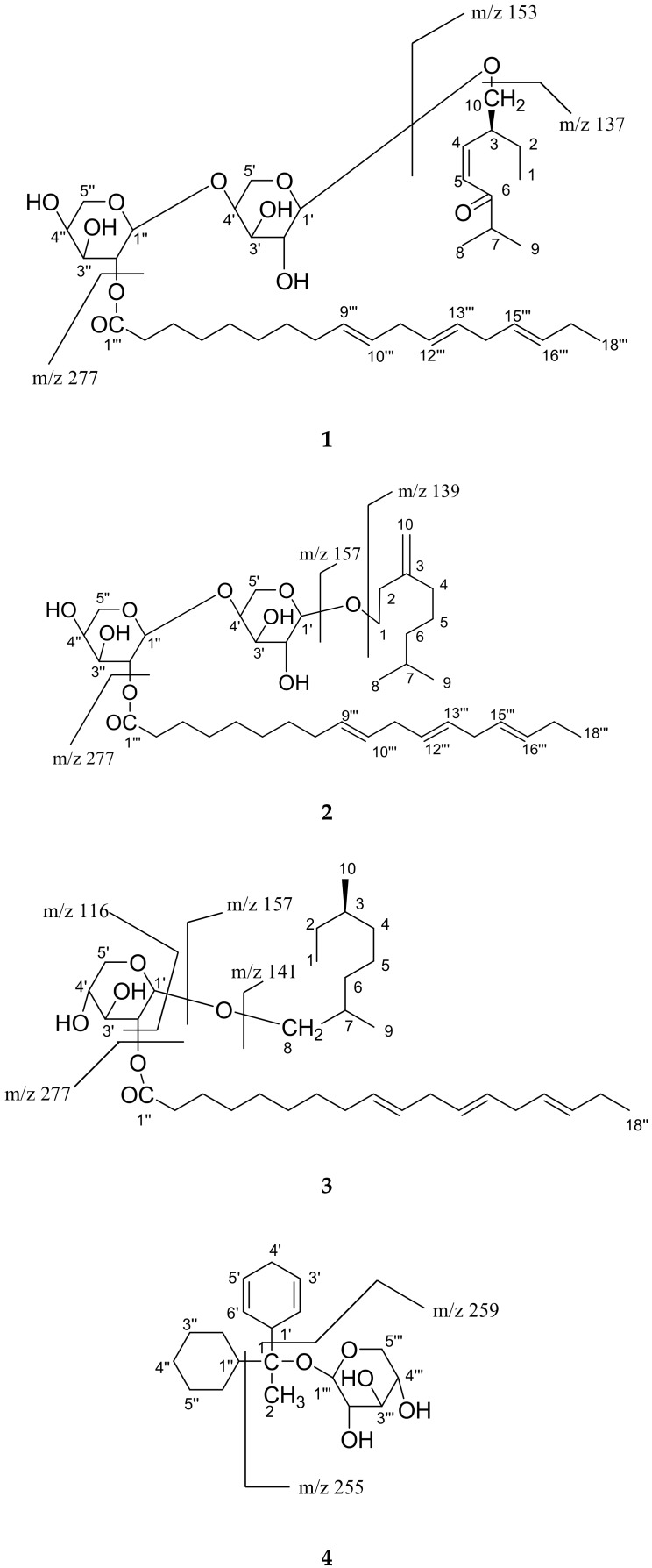
Mass fragmentation of compounds (**1**–**4**) isolated from *D. morbifera*.

Compound **1** was obtained as a white semi-solid. The IR absorption bands at 3425, 3363 and 3271 cm^−1^ indicated the presence of hydroxyl groups. Its molecular ion peak at *m*/*z* 695 [M + H]^+^ was determined based on basis of (FAB) mass and ^13^C NMR spectra consistent with the molecular ion peak of a monoterpene sugar, indicating eight degrees of unsaturation. (ESIFTMS)provided the exact mass of the protonated molecular ion *m*/*z* (695.4371), from which the molecular composition C_38_H_63_O_11_ was calculated. The fragmentation patterns of compound **1** are shown in Figure 3.

The ^1^H NMR spectrum of **1** showed one-proton and two-proton multiplets which at δ 5.34 (H-10‴), 5.32 ((H-12‴), H-13‴), 5.30 (H-15‴),, 5.24 (H-16‴), 3.46 (H-2′), 4.01 (H-3′), 3.93 (H-4′), 4.71(H-2″), 4.20 (H-3″), 4.15 (H-4″), 3.04 (H-3), 2.47 (H-7), 2.34 (H-11‴), 2.21 (H-14‴), 2.05 (H_2_-8‴), 2.01 (H-14‴), 1.79 (H-2), 1.61 (H_2_-3‴), where these positions were assigned as as shown. Other methylene protons as multiplets at δ 1.30, 1.35, and other methylene protons resonating between δ 2.05–1.56. A triplet at δ 2.77 appeared for methylene protons attached with keto group and assigned for H_2_-2‴. The olefinic protons in themonoterpene moiety appeared double doublet and doublet at δ 6.28 (dd, *J* = 7.0, 6.8 Hz, H-4) and 5.34 (d, *J* = 7.0 Hz)) and anomeric protons appeared as a doublets at δ 4.92 (d, *J* = 8.5 Hz, H-1′) and 4.90 (d, *J* = 8.0 Hz, H-1″). A three-proton triplets appeared at δ 0.88 (*J* = 7.1 Hz), 0.86 (*J* = 6.5 Hz), and 0.83 (*J* = 6.3 Hz), assigned for Me-8, Me-9, and Me-18‴, respectively. The sugar unit in **1** wereidentified as β-d-arabinopyranose by an analysis of thecoupling constants of the anomeric signals of the sugar protons, as one proton, two doublets δ 4.92 (d, *J* = 8.5 Hz, H-1′), 4.90 (d, *J* = 8.0 Hz, H-1″) and the remaining sugar protons resonated as multiplets at δ 3.34–4.71.

**Figure 4:**
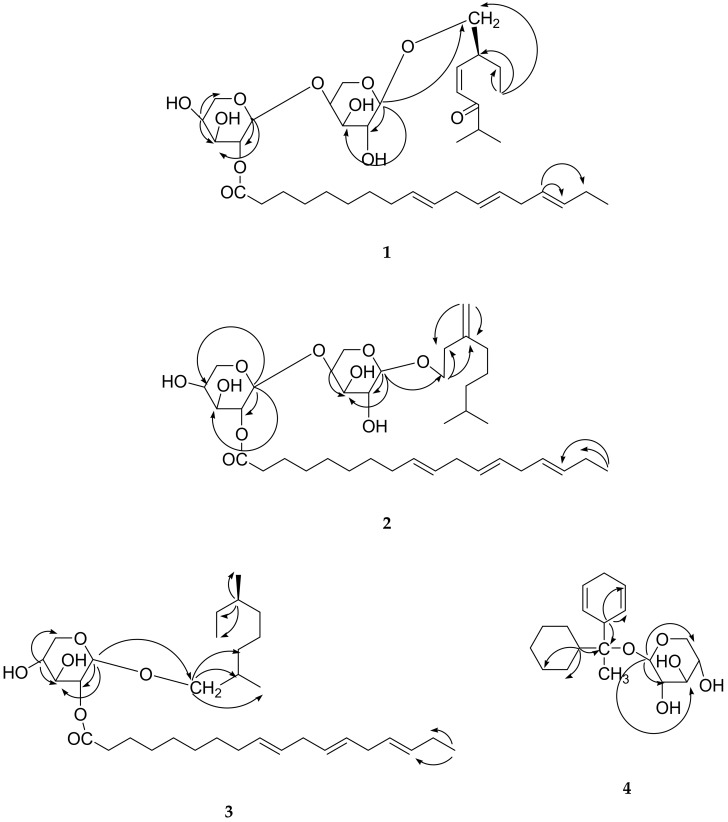
Heteronuclear multiple-bond correlation spectroscopy (HMBC). Correlation of compounds (**1**–**4**) isolated from *D. morbifera*.

The ^13^C NMR spectrum of **1** displayed 38 carbon signals, with 28 attributed to the aglycone part and 10 to thesugar units. Important carbon signals appeared for anomeric carbons δ 109.13 (C-1′) and δ 107.36 (C-1″), and the other sugar carbons resonated between δ 78.92 to δ 63.03, carbonyl carbon δ 202.41 (C-6), and methyls carbons δ 17.92 (C-1), 18.74 (C-9) and 19.82 (C-8), and methylene and methines carbons in monoterpene at δ 24.78 (C-2), and 17.66 (C-3), 54.74 (C-7), and olefinic carbons at δ 142.99 (C-4) and 136.26 (C-5). The ^1^H-^1^H correlation spectroscopy (COSY) spectrum of **1** showed correlations of H-1 with H-2 and H-3; H-7 with H-8 and H-9, H-9; H-1″ with H-2″; H-12‴ with H-11‴ and H-13″.Theheteronuclear single quantum coherence(HSQC) spectrum of **1** showed important correlations of H_3_-1 at δ 0.86 with C-1 at δ 17.92; H_2_-10 at δ 3.58 with C-10 at δ 62.37; H-9‴ at δ 5.24 with C-9‴ at δ 132.86; H-12‴ at δ 5.32 with C-12‴ at δ 129.07; H-1′ at δ 4.92 with C-1′ at δ 109.13; H-5′ at δ 3.83 with C-5′ at δ 63.32; H-5″ at δ 3.63 with C-5″ at δ 63.03; H-4 at δ 6.28 with C-4 at δ 142.99; H-5 at δ 5.48 with C-5 at δ 136.26.Theheteronuclear multiple-bond correlation spectroscopy (HMBC) spectrum of **1** (Figure 4) exhibited interactions H-1′ with C-2′, C-3′ and C-10; H-1″ with C-2″, C-3″, C-4″and C-5″; H-4′ with C-2′, C-3′ and C-5′; H-4″ with C-2″, C-3″ and C-5″; H-7 with C-5, C-8 and C-9; H-1 with C-2, C-3 and C-10; H-16″ with C-17″ and C-18″. The COSY, HSQC and heteronuclear multiple-bond correlation spectroscopy (HMBC)are also in agreement with the compound **1**. The ^1^H and ^13^C NMR data of the sugar was also compared through previous literature [12,17]. On the basis of these evidences, the structure of **1** was established as *cis*-6-oxogeran-4-enyl-10-oxy-*O*-β-arabinopyranosyl-4′-*O*-β-arabinopyranosyl-2″-octadec-9‴,12‴,15‴-trienaote (**1**). This is a new compound, and has here been reported for the first time in nature.

Compound **2** was obtained as a white semi-solid. The IR absorption bands at 3425, 3371 and 3280 cm^−1^ indicated the presence of hydroxyl groups. Its molecular ion peak at *m*/*z* 681 [M + H]^+^ was determined based on FAB mass and ^13^C NMR spectra consistent with the molecular ion peak of a monoterpene sugars indicating seven degrees of unsaturation. High resolution of ESIFTMS provided the exact mass of the protonated molecular ion *m*/*z* (681.4578), from which the molecular composition C_38_H_65_O_10_ was calculated. The fragmentation patterns of compound **2** are shown in Figure 3.

The ^1^H NMR spectrum of **2** showed one-proton multiplets at δ 6.01, 5.97, 5.48, 5.43, 5.34 and 5.23, where these were assigned to the H-12‴, H-13‴, H-10‴, H-15‴, H-9‴ and H-16‴, respectively. The exo-olefinic methylene protons in themonoterpene moiety as broad singlet at δ 4.96 and 4.94 (br s, H_2_-10a, 10b) and other neighboring methylene protons appeared as multiplets at δ 2.41, 1.98 (m, H_2_-4, H_2_-2) and methine protons at δ 1.80 (m, H-7). Other methylene protons in long-chain appeared as multiplets which resonating between at δ 1.68–1.28. A three-proton triplet at δ 0.93 (*J* = 6.5 Hz), and three-proton two doublets appeared atδ 0.88, 0.85, assigned for Me-18‴ and Me-8, Me-9, respectively.

The sugar units in **2** wereidentified as β-d-arabinopyranose by an analysis of thecoupling constants of the anomeric signals of the sugar protons as one-proton two doublets δ 4.84 (d, *J* = 8.0 Hz, H-1′), 4.80 (d, *J* = 8.0 Hz, H-1″) and methine protons two double doublets appeared at δ 3.76 ( dd, *J* = 6.5, 8.0 Hz, H-2′), 4.10 (dd, *J* = 7.5, 8.0 Hz, H-2″), and methylene protons two doublets appeared at δ 3.64 (d, *J* = 6.5 Hz, H-5′), 3.61 (d, *J* = 6.5 Hz, H-5″). The remaining sugar protons resonated between as multiplets at δ 3.53–3.44 for H-3′, 3″, 4′, 4″.

The ^13^C NMR spectrum of **2** displayed 38 carbon signals, with 28 attributed to the aglycone part and 10 to thesugar units. Important carbon signals appeared for anomeric carbons δ 107.55 (C-1′) and δ 105.61 (C-1″), and the other sugar carbons resonated between δ 89.82 to δ 62.97, carbonyl carbon (δ 170.15), and methyl carbons δ 20.78 (C-8), 20.68 (C-9) and 14.03 (C-18‴), and methylene carbons in monoterpene δ 24.68 (C-2), 21.19 (C-4), 25.67 (C-5), 21.89 (C-6). The ^1^H-^1^H COSY spectrum of **2** showed correlations of H-1 with H-2; H-7 with H-8 and H-6, H-9; H-1″ with H-2″; H-12‴ with H-11‴ and H-13″. The HSQC spectrum of **2** (Figure 3) showed important correlations of H_2_-1 at δ 3.02 with C-1 at δ 60.87; H-9‴ at δ 5.23 with C-9‴ at δ 137.99; H-12‴ at δ 6.01 with C-12‴ at δ126.07; H-1′ at δ 4.84 with C-1′ at δ 107.55; H-1″ at δ 4.80 with C-1″ at δ 105.61; H-5′ at δ 3.64 with C-5′ at δ 63.36; H_2_-6 at δ1.98 with C-6 at δ 21.89. The HMBC spectrum of **1** (Figure 4) exhibited interactions H-1′ with C-2′, C-3′ and C-1; H-1″ with C-2″, C-3″ and C-4″; H-4′ with C-2′, C-3′ and C-5′; H-4″ with C-2″, C-3″ and C-5″; H-8 with C-6, C-7 and C-9; H-1 with C-2, C-3 and C-10; H-18″ with C-16″ and C-17″. The ^1^H and ^13^C NMR data of the compound **2** was also compared through previous literature [12,17]. On the basis of these evidences, the structure of **2** was established as geran-3(10)-enyl-1-oxy-*O*-β-arabinopyranosyl-4′-*O*-β-arabinopyranosyl-2″-octadec-9‴, 12‴, 15‴-trienoate (**2**). This is a new compound, and is reported here the first time in nature.

Compound **3** was obtained as a white semi-solid. The IR absorption band at 3315 cm^−1^ indicated the presence of a hydroxyl group. Its molecular ion peak at *m*/*z* 551 [M + H]^+^ was determined on the basis of FAB mass and ^13^C NMR spectra consistent with the molecular ion peak of a monoterpene sugar derivatives, indicating five degrees of unsaturation. High resolution of ESIFTMS provided the exact mass of the protonated molecular ion *m*/*z* (551.4312), from which the molecular composition C_33_H_59_O_6_ was calculated. The fragmentation patterns of compound **3** are shown in Figure 3.

The ^1^H NMR spectrum of **3** shown one-proton multiplets at δ 5.82, 5.71, 5.49, 5.47, 5.36 and 5.24, and these were assigned to the H-13″, H-12″, H-10″, H-15″, H-9″ and H-16″, respectively. The sugar unit in **3** was identified as *α*-xylopyranose by theanalysis of coupling constants of the anomeric signals of the sugar protons as a one-proton doublet δ 4.37 (d, *J* = 6.5 Hz, H-1′). The remaining sugar protons appeared as one-proton multiplets at δ 4.15, 4.01, δ 3.88 for H-2′ to H-4′ and methylene protons in sugar appeared at δ 3.65 (d, *J* = 6.5 Hz, H_2_-5′). Another methylene proton between sugar and theacyclic skeleton appeared as a doublet at δ 3.62 (d, *J* = 6.5 Hz, H_2_-8). The other methylene protons in long-chain attached with a sugar unit appeared as multiplets which resonated between δ 2.07 to 1.78 (H_2_-8″, H_2_-11″, H_2_-14″ and H_2_-17″), and methylenes protons attached with the keto group appeared as a triplet at δ 2.34 (t, *J* = 7.0 Hz, H_2_-2″). The remaining methylene and methine protons resonated between the range δ 1.72 to 1.30, and the protons of the methyl resonated in the range of δ1.25 to 0.87. The ^13^C NMR spectrum of **3** displayed 33 carbon signals, with 28 attributed to the aglycone part, and five carbons of sugar unit. Important carbon signals appeared for anomeric carbons δ 80.59 (C-1′) and δ 80.06 (C-2″), and the other sugar carbons resonated between δ 70.27 to δ 62.99, carbonyl carbon (δ 174.12), and methyl carbons δ 17.65 (C-1), 20.45 (C-29) and 9.27 (C-10). The ^1^H-^1^H COSY spectrum of **3** showed correlations of H-1 with H-2 and H-3; H-7 with H-8 and H-6, H-9; H-1′ with H-2′; H-9″ with H-8″ and H-10″. The HSQC spectrum of **3** showed important correlations of H-1 at δ 0.90 with C-1 at δ 17.65; H-9″ at δ 5.36 with C-9″ at δ 121.98; H-12″ at δ 5.71 with C-12″ at δ 146.16; H-1′ at δ 4.37 with C-1′ at δ 85.59; H-5′ at δ 4.15 with C-5′ at δ 62.99; H_2_-6 at δ 1.57 with C-6 at δ 34.10. The HMBC spectrum of **3** (Figure 4) exhibited interactions H-1′ with C-2′, C-3′ and C-8; H-4′ with C-2′, C-3′ and C-5′; H-8 with C-6, C-7 and C-9; H-3 with C-1, C-2 and C-10; H-18″ with C-16″ and C-17″. The ^1^H and ^13^C NMR data of the compound **3** sugar was also compared through previous literature [12,17].

On the basis of these evidences, the structure of **3** was established as geranilan-8-oxy-*O*-*α*-d-xylopyranosyl-2′-*n*-octadec-9″, 12″, 15″-trienoate (**3**). This is a new compound, and was reported the first time here in nature.

Compound **4** was obtained as a white semi-solid. The IR absorption band at 3415 and 3359 cm^−1^ indicated the presence of hydroxyl group, and double bonds in themolecule appeared at 1635 cm^−1^. Its molecular ion peak at *m*/*z* 339 [M + H]^+^ was determined based on FAB mass and ^13^C NMR spectra consistent with the molecular ion peak of a compound indicating five degrees of unsaturation. High resolution of ESIFTMS provided the exact mass of the protonated molecular ion *m*/*z* (339.2171), from which the molecular composition C_19_H_31_O_5_ was calculated. The fragmentation patterns of compound **4** are shown in Figure 3.

The ^1^H NMR spectrum of **4** for vinylic protons showed double doublets appeared at δ 5.51 (*J* = 4.5, 6.0 Hz) and 5.90 (*J* = 4.5, 6.0 Hz) were assigned for H-2′ and H-6′ protons. Two multiplets at δ 5.16 and 5.38 and one doublet at δ 5.39 (*J* = 7.0 Hz) were also assigned for H-3′ and H-5′ and H-1‴ protons. The ^1^H NMR spectrum of **4** showed that for one and two protons, multiplets appeared at δ 4.80, 1.60, 4.83, 2.06, 1.53, 1.50, 1.37, and these protons were assigned for H_2_-5‴, H-4‴, H-4′, H_2_-2″, H_2_-6″, H_2_-5 ″ and H_2_-4′. The ^13^C NMR spectrum of compound **4** displayed 19 carbon signals, with 14 attributed to the aglycone part, and five carbons of sugar units. Important carbon signals appeared for anomeric carbon δ 76.13 (C-1′), and other sugar carbons δ 71.05 (C-2‴), 65.01 (C-3‴), 63.87 (C-4‴), and 63.03 (C-5‴), vinylic carbons δ 138.10 (C-2′), 123.52 (C-3′), 116.42 (C-5′), and 133.84 (C-6′). The ^1^H-^1^H COSY spectrum of **4** showed correlations of H-1′ with H-2′ and H-6′; H-2′ with H-3′; H-5′ with H-6′; H-1″ with H-2″ and H-16″; H-1‴ and H-2″.The HSQC spectrum of **4** showed important correlations of H-1′ at δ 3.03 with C-1′ at δ 18.07;H-1″ at δ 1.60 with C-1″ at δ 33.65;H-1‴ at δ 5.39 with C-1‴ at δ 63.03; H-2′ at δ 5.90 with C-2′ at δ 138.10; H-3′ at δ 5.38 with C-3′ at δ 123.52; H-5′ at δ 5.16 with C-5′ at δ 116.42; H-6′ at δ 5.51 with C-6′ at δ 133.84. The HMBC spectrum of **4** (Figure 4) exhibited interactions H-1′ with C-2′, C-3′ and C-1; H-1″ with C-2″, C-3″ and C-1; H-1‴ with C-2‴, C-3‴ and C-5‴.The ^1^H and ^13^C NMR data of the sugar was also compared through previous literature [12,17]. On the basis of these evidences, the structure of **4** was established as 1-cyclohex-2′,5′-dienyl 1-cyclohexylethanol-*O*-β-d-xylopyranoside (**4**). This is a new compound, herer eported for the first time in nature.

### 2.1. Antioxidant Activity

#### 2.1.1. Free Radical Scavenging Activity

The free radical scavenging activities of the mono- and disaccharide with monoterpene along with aliphatic chain-isolated compounds from *Dendropanax* were tested using the DPPH method [18]. Table 1 presents the antioxidant activity of four compounds at the concentration of 1.0 mg/mL as systematic by the DPPH scavenging assay. The IC_50_ values of the entire four constituents were **1**–**4** (10 µg/mL), (25 µg/mL), (50 µg/mL) and (100 µg/mL), respectively. Of the different compounds isolated from the acetone extract from the Dendropanax bark, compounds **1** and **2** exhibited the highest activity, which was more than about 65% and 50% at 100 µg/mL concentration, respectively, when compared with the compounds **3** and **4** (Table 1). The compounds **3** and **4** demonstrated moderate antioxidant activity. The DPPH activity of tocopherol showed a higher degree of free radical-scavenging activity (91%) than that of the compounds at a very low concentration point (100 µg/mL). Our finding for antioxidant activities in four different sugar containing compounds support our previous results obtained in fruits of *Lycium barbarum* and *Lycium chinense* [19,20]. This is similar to other studies, wherein they have reported that only 300 µg/mL tocopherol, 230 µg/mL BHT (Butylated hydroxytoluene) and 100 µg BHA (Butylated hydroxyanisole) exhibited a free radical scavenging activity equivalent to 390 µg/mL of red bean and 1000 µg/mL of sesame coat extract [20,21].

#### 2.1.2. Reducing Power

The antioxidant effect exponentially increases as a function of the development of the reducing power, indicating that the antioxidant properties concomitant with the development of reducing power have been reported [22,23], that the reducing power of such type of compounds from medicinal plants prevents liver injury by inhibiting formation of lipid peroxides. As seen in Table 1, the reducing power of the constituents **1**–**4** from the Dendropanax bark isolated compounds enhanced with escalating concentration from 10 to 100 µg/mL. Reducing power of the compounds **1**–**4** followed in the order **1** > **2** > **3** > **4**. The antioxidant potential of tocopherol was markedly greater than the test samples at a very low concentration point. This finding also supports the outcome of other researchers, where the reducing power of BHT and tocopherol [20,24] was higher than the isolated compounds.

#### 2.1.3. Nitric Oxide Scavenging Activity

Nitric oxide free radicals were improved and scavenged by compounds **1** and **2** in comparison with compounds **3** and **4** at low concentrations of 10–100 µg/mL. Scavenging of NO was well interacted with the existence of sugars in compounds, and the sequence was found to be 1 > 2> 3 >4 (Table 1).

#### 2.1.4. Antioxidant Capacity by Phosphomolybdenum Method

Antioxidant potential of compounds **1**–**4** was measured spectrophotometrically by the phosphomolybdenum method, which is based on the reduction of Mo (IV) to Mo (V) by the sample analyte, ensuing the formation of greenish phosphate/Mo (V) compounds with a maximum absorption at 695 nm.

The antioxidant potential of compounds **1**–**4** was established as 15.31, 13.20, 6.70 and 5.63 µg/mL, respectively. The antioxidant capacities of the compounds were found to be in the order of **1** > **2** > **3** > **4** (Table 2).

## 3. Materials and Methods

### 3.1. Chemical and Instruments

Melting points of the compounds were determined using a model IA9100 melting point apparatus (Electrochemical Engineering, Seoul, Korea). Optical rotations were measured with a model AA-10 polarimeter (Instrument Ltd., Seoul, Korea). Infrared (IR) spectra were recorded after compound mixing with potassium bromide (KBr) on a Thermo Scientific FT-IR model Nicolet 6700 (Thermo Fisher Scientific, Waltham, MA, USA) spectrophotometer at the Korea Institute of Science and Technology (KIST), Seoul, Korea. Both nuclear magnetic resonance (NMR) spectra ^1^H (600 MHz) and ^13^C NMR (150 MHz) were measured with a Bruker Avance-600 spectrometer (Bruker Corporation, Billerica, MA, USA) using deuterated solvents, and the machine was available at the National Instrumentation Centre for Environmental Management (NICEM), College of Agriculture and Life Science, Seoul National University (SNU), Seoul, Korea. NMR spectra were recorded in deuterated chloroform, and methanol-d_4_ using tetramethylsilane (TMS) as an internal standard, with chemical shifts expressed in parts per million (*d*) and coupling constants (*J*) in Hertz. High-resolution electrospray ionization Fourier transform (ESI/FT) mass spectra were recorded on a Thermo-Finnigan LTQ-Orbitrap instrument (Thermo Scientific, Bartlesville, OK, USA) equipped with a Dionex U 3000 HPLC system with UV-VIS detector (SPD-10A) (Dionex Corporation, Sunnyvale, CA, USA). A C_18_ (octadecylsilyl, or ODS) column was used with a mobile phase of 0.1% TFA (Trifluoroacetic acid) in acetonitrile: water (80:20), and a flow rate of 4 mL^−1^. All chemicals were of analytical grade. *n*-Hexane, ethyl acetate, methanol, ethanol, sulfuric acid (H_2_SO_4_) and vanillin were purchased from Daejung Chemicals and Metals (Seoul, Korea). Thin-layer chromatography (TLC) was performed on pre-coated silica gel 60 F_254_ plates (Merck, Darmstadt, Germany). Visualization of TLC plates was performed in a developing glass chamber, and after drying, they were dipped in solution of 5% vanillin and H_2_SO_4_ in an ethanol spray reagent (5:5:90). Column chromatography was performed using silica gel (70–230 mesh) and LiChroprep RP-18 (40–63 µm; Octadecyl silica (ODS) gel) from Merck. Standards were purchased from Sigma-Aldrich (St. Louis, MO, USA).

### 3.2. Plant Material

Dried bark (558 g) of *D. morbifera* was collected from Konkuk University Farm, Seoul, South Korea. A voucher specimen (No. DML-KU-2013) has been deposited at the Department of Applied Bioscience, Konkuk University, Seoul, South Korea.

### 3.3. Extraction and Isolation

The powdered bark of *D. morbifera* (558 g) was immersed in acetone (3 × 2.5 L) for three days at room temperature, and then the supernatant was concentrated under vacuum to yield 24.5 g of an extract.

The acetone extract was subjected to column chromatography on silica gel (70–230 mesh, 500 g, 4.5 × 95 cm) and eluted with a gradient of *n*-hexane/CHCl_3_/MeoHto yield 34 fractions (each of 250 mL): Frs. 1–4 with *n*-hexane, frs. 5–6 with *n*-hexane–CHCl_3_ (7.5:2.5), frs. 7–8 with *n*-hexane–CHCl_3_ (1:1), frs. 9–10 with *n*-hexane–CHCl_3_ (2.5:7.5), frs. 11–12 with CHCl_3_, frs. 13–14 with CHCl_3_–MeOH (99:1), frs. 15–16 with CHCl_3_–MeOH (98:2), frs. 17–18 with CHCl_3_–MeOH (97:3), frs. 19–20 with CHCl_3_–MeOH (96:4), frs. 21–22 with CHCl_3_–MeOH (95:5), frs. 23–24 with CHCl_3_–MeOH (94:6), frs. 25–26 with CHCl_3_–MeOH (93:7), frs. 27–28 with CHCl_3_–MeOH (92:8), frs. 29–30 with CHCl_3_–MeOH (9:1), frs. 31–32 with CHCl_3_–MeOH (8.8:1.2), frs. 33–34 with CHCl_3_–MeOH (8.5:1.5). Fractions 13–14 (2.4 g) were chromatographed over LiChroprep RP-18 (ODS silica gel; 40–63 μm: 100 g; 45 × 2 cm, each fraction 100 mL). Fractions 29–30 of the first column after additional rechromatography with elution sequentially performed with CH_3_OH–H_2_O, yielded 10 fractions: Frs. 1–2 with H_2_O–MeOH (1:1), frs. 3–4 with H_2_O–MeOH (2:8), frs. 5–6 with H_2_O-MeOH (1:9), frs. 7–10 with MeOH. Frs. 1–2 (0.8 g, 100 mL fraction) after rechromatography over silica gel withchloroform and methanol. The elution was sequentially performed with chloroform containing methanol 0.2%, 0.4%, 0.6%, 0.8% and 1.0% to yield four new compounds **1** (32 mg), **2** (28 mg), **3** (29 mg) and **4** (41 mg) and fractions 13–14 and from other fractions after rechromatography were isolated six more known compounds **5** (26 mg), **6** (30 mg), **7** (38 mg), **8** (21 mg), **9** (31 mg) and **10** (41 mg; Figure 2).

*Cis*-6-Oxogeran-4-enyl-10-oxy-*O*-β-arabinopyranosyl-4′-*O*-β-arabinopyranosyl-2″-octadec-9‴,12‴,15‴-trienoate (**1**)

White solid; mp 176–178 °C;, R_f_ 0.28 (CHCl_3_;MeOH; 9.4:0.6); [α]_D_^23^ -28.4 (*c* 0.1, MeOH); IR *ν_max_* (KBr): 3425, 3363, 3271, 2927, 2856, 1721, 1701, 1643, 1457, 1389, 1242, 1187, 1033, 891 cm^−1^; ^1^H NMR (MeOD; 600 MHz): δ 6.28 (1H, dd, *J* = 7.0, 6.8 Hz, H-4), 5.48 (1H, d, *J* = 7.0 Hz, H-5), 5.34 (1H, m, H-10‴), 5.32 (2H, m, H-12‴, H-13″), 5.30 (2H, m, H-10‴, H-15″), 5.24 (1H, m, H-16‴), 4.92 (1H, d, *J* = 8.5 Hz, H-1′), 3.46 (1H, m, H-2′), 4.01 (1H, m, H-3′), 3.93 (1H, m, H-4′), 3.83 (1H, d, *J* = 4.5 Hz, H_2_-5′a), 3.63 (1H, d, *J* = 4.0 Hz, H_2_-5′b), 4.90 (1H, d, *J* = 8.0 Hz, H-1″), 4.71 (1H, m, H-2″), 4.20 (1H, m, H-3″), 4.15 (1H, m, H-4″), 3.63 (1H, d, *J* = 6.5 Hz, H_2_-5″a), 3.61 (1H, d, *J* = 6.0 Hz, H_2_-5″b), 3.58 (1H, d, *J* = 6.0 Hz, H_2_-10a), 3.50 (1H, d, *J*=7.5 Hz, H_2_-10b), 3.04 (1H, m, H-3), 2.77 (2H, t, *J*=7.0 Hz, H_2_-2‴), 2.47 (1H, m, H-7), 2.34 (2H, m, H_2_-11‴), 2.21 ((2H, m, H_2_-14‴), 2.05 (2H, m, H_2_-8‴), 2.01 (2H, m, H_2_-14‴), 1.79 (2H, m, H_2_-2), 1.61 (2H, m, H_2_-3‴), 1.56 (2H, m, CH_2_), 1.35 (2H, m, CH_2_), 1.30 (4H, br s, 2 × CH_2_), 0.90 (3H, d, *J* = 7.5 Hz, Me-8), 0.88 (3H, t, *J* =7.1 Hz, Me-9), 0.86 (3H, t, *J* = 6.5 Hz, Me-1), 0.83 (3H, t, *J* = 6.3 Hz, Me-18‴); ^13^C NMR (MeOD; 150 MHz): δ 17.92 (C-1), 24.78 (C-2), 17.66 (C-3), 142.99 (C-4), 136.26 (C-5), 202.41 (C-6), 54.74 (C-7), 19.82 (C-8), 18.74 (C-9), 62.37 (C-10), 109.13 (C-1′), 78.92 (C-2′), 74.28 (C-3′), 70.26 (C-4′), 63.32 (C-5′), 107.36 (C-1″), 74.97 (C-2″), 72.59 (C-3″), 65.15 (C-4″), 63.03 (C-5″), 174.27 (C-1‴), 52.01 (C-2‴), 29.56 (C-3‴), 29.39 (C-4‴), 29.12 (C-5‴), 27.16 (C-6‴), 25.40 (C-7‴), 31.37 (C-8‴), 132.86 (C-9‴), 129.89 (C-10‴), 34.11 (C-11‴), 129.61 (C-12‴), 127.73 (C-13‴), 32.74 (C-14‴), 128.23 (C-15‴), 122.47 (C-16‴), 22.61 (C-17‴), 14.02 (C-18‴); FABMS *m*/*z* (rel.int.) 695 [M+H]^+^ (11.2); (2.1), 525 (3.2), 277 (15.6 ), 261 (5.8); HR-ESIMS *m*/*z* (rel. int.): 695.4368 [M + H]^+^ (calcd. 695.4371 for C_38_H_63_O_11_).

Geran-3(10)-enyl-1-oxy-*O*-β-arabinopyranosyl-4′-*O*-β-arabinopyranosyl-2″-octadec-9‴,12‴,15‴-trienoate (**2**)

White solid, mp. 180–182 °C; R_f_ 0.31 (CHCl_3_; MeOH; 9.4:0.6); [α]_D_^23^ -20.4 (*c* 0.1, MeOH); IR *ν_max_* (KBr): 3425, 3371, 3280, 2931, 2889, 1721, 1635, 1456, 1369, 1226, 1173, 1032, 973, 889, 755 cm^−1^; ^1^H NMR (MeOD; 600 MHz): δ 6.01 (1H, m, H-12‴), 5.97 (1H, m, H-13‴), 5.48 (1H, m, H-10‴), 5.43 (1H, m, H-15‴), 5.34 (1H, m, H-16‴), 5.23 (1H, m, H-9‴), 4.96 (1H, br s, H_2_-10a), 4.90 (1H, br s, H_2_-10b), 4.84 (1H, d, *J* = 8.0 Hz, H-1ꞌ), 3.76 (1H, dd, *J* = 6.5, 8.0 Hz, H-2′), 3.53 (1H, m, H-3′), 3.46 (1H, m, H-4′), 3.64 (2H, d, *J* = 6.5 Hz, H_2_-5′), 4.80 (1H, d, *J* = 8.0 Hz, H-1″), 4.10 (1H, dd, *J* =7.5, 8.0 Hz, H-2″), 3.49 (1H, m, H-3″), 3.44 (1H, m, H-4″), 3.61 (2H, d, *J* = 6.5 Hz, H_2_-5″), 3.02 (2H, dd, *J* = 6.0, 6.5 Hz, H_2_-1), 2.60 (2H, t, *J* =7.3 Hz, H_2_-2‴), 2.41 (2H, m, H_2_-4), 2.38 (2H, m, H_2_-11‴), 2.30 (2H, m, H_2_-14‴), 2.19 (2H, m, H_2_-8‴), 2.05 (2H, m, H_2_-17‴), 1.98 (2H, m, H_2_-2), 1.80 (1H, m, H-7), 1.68 (2H, m, CH_2_), 1.63 (2H m, CH_2_ ), 1.49 (2H, m, CH_2_ ), 1.30 (4H, m, 2 × CH_2_), 1.28 (4H, m, 2 × CH_2_), 0.93 (3H, t, *J* = 6.5 Hz, Me-18‴), 0.88 (3H, d, *J* = 6.5 Hz, Me-8), 0.85 (3H, d, *J* = 6.6 Hz. Me-9); ^13^C NMR (MeOD; 150 MHz): δ 60.87 (C-1), 24.68 (C-2), 153.38 (C-3), 21.19 (C-4), 25.67 (C-5), 21.89 (C-6), 57.76 (C-7), 20.78 (C-8), 20.68 (C-9), 110.55 (C-10), 107.55 (C-1′), 79.32 (C-2′), 72.51 (C-3′), 70.43 (C-4′), 63.36 (C-5′), 105.61 (C-1″), 89.92 (C-2″), 75.97 (C-3″), 66.28 (C-4″), 62.97 (C-5″) 170.15 (C-1‴), 52.46 (C-2‴), 29.57 (C-3‴), 29.75 (C-4‴), 29.91 (C-5‴), 26.41 (C-6‴), 27.47 (C-7‴), 31.83, (C-8‴), 137.99 (C-9‴), 129.60 (C-10‴), 36.18 (C-11‴), 126.07 (C-12‴), 125.82 (C-13‴), 34.47 (C-14‴), 121.78 (C-15‴), 114.45 (C-16‴), 22.57 (C-17‴), 14.03 (C-18‴); FABMS *m*/*z* (rel. int.) 681 [M + H]^+^ (7.2); HR-ESIMS *m*/*z* (rel. int.): 681.4571 [M + H]^+^ ((calcd. 681.4578 for C_38_H_65_O_10_).

Geranilan-8-oxy-*O*-α-d-xylopyranosyl-2′-n-octadec-9″,12″,15″-trienoate (**3**)

White semi-solid, R_f_ 0.35 (CHCl_3_;MeOH; 9.5:0.5); [α]_D_^23^ -21.4 (*c* 0.1, MeOH); IR *ν_max_* (KBr): 3355, 2928, 2857, 1721, 1635, 1457, 1370, 1226, 1123, 1041, 754 cm^−1^; ^1^H NMR (MeOD; 600 MHz): δ 5.82 (1H, m, H-13″), 5.71 (1H, m, H-12″), 5.49 (1H, m, H-10″), 5.47 (1H, m, H-15″), 5.36 (1H, m, H-9″), 5.24 (1H, m, H-16″), 4.37 (1H, d, *J* = 6.5 Hz, H-1), 4.15 (1H, m, H-2′), 4.01 (1H, m, H-3′), 3.88 (1H, m, H-4′), 3.65 (2H, d, *J* = 6.5 Hz, H_2_-5′), 3.62 (2H, d, *J* = 6.5 Hz, H_2_-8), 2.34 (2H, t, *J* =7.0 Hz, H_2_-2″), 2.07 (2H, m, H_2_-11″), 2.05 (2H, m, H_2_-14″), 1.80 (2H, m, H_2_-8″), 1.78 (2H, m, H_2_-17″), 1.72 (1H, m, H-7), 1.61 (1H, m, H-3), 1.57 (2H, m, H_2_-6), 1.54 (2H, m, H_2_-5), 1.30 (14 H, br s, 7 × CH_2_), 1.25 (3H, d, *J* = 6.2 Hz, Me-9), 1.06 (3H, d, *J* = 6.0 Hz, Me-10), 0.90 3H, t, *J* = 6.5 Hz, Me-1), 0.87 (3H, t, *J* = 6.3 Hz, Me-18″); ^13^C NMR (MeOD; 150 MHz): δ 17.65 (C-1), 25.01(C-2), 36.14 (C-3), 24.59 (C-4), 33.66 (C-5), 34.10 (C-6), 36.48 (C-7), 63.39 (C-8), 20.45 (C-9), 9.27 (C-10), 85.59 (C-1′), 80.06 (C-2′), 70.27 (C-3′), 65.42 (C-4′), 62.99 (C-5′), 174.12 (C-1″), 34.10 (C-2″), 29.33 (C-3″), 29.24 (C-4″), 29.16 (C-5″), 28.46 (C-6″), 27.14 (C-7″), 31.81 (C-8″), 121.98 (C-9″), 133.05 (C-10″), 32.64 (C-11″), 146.16 (C-12″), 137.93 (C-13″), 32.16 (C-14″), 129.58 (C-15″), 117.17 (C-16″), 22.60 (C-17″), 14.05 (C-18″); FABMS *m*/*z* (rel.int.) 551 [M + H]^+^ (4.2); (1.1), 277 (51.8), 157 (18.2), 141 (22.5); HR-ESIMS *m*/*z* (rel. int.): 551.4316 [M + H]^+^ (calcd. 551.4312 for C_33_H_59_O_6_).

1-cyclohex-2′, 5′-dienyl 1-cyclohexylethanol-*O*-β-d-xylopyranoside (**4**)

Semi-solid; R_f_ 0.28 (CHCl_3_; MeOH; 9.5:0.5); [α]_D_^23^ -31.4 (*c* 0.1, MeOH); IR *ν_max_* (KBr): δ 3425, 3359, 2927, 2855, 1635, 1594, 1459, 1419, 1226, 1122, 1027, 967 cm^−1^; ^1^H NMR (MeOD; 600 MHz): 5.90 (1H, dd, *J* = 4.5, 6.0 Hz, H-2′), 5.51 (1H, dd, *J* = 4.5 Hz, 6.0, 5.5 Hz, H-6′), 5.38 (1H, m, H-3′), 5.16 (1H, m, H-5′), 5.39 (1H, d, J=7.0 Hz, H-1‴), 4.89 (1H, m, H-2‴), 4.83 (2H, br s, H_2_-5‴), 4.80 (1H, m, H-4‴), 3.53 (1H, dd, *J*= 6.5, 6.5 Hz, H-3‴), 3.03 (1H, d, *J* = 6.0 Hz, H-1′), 2.06 (2H, m, H_2_-4′), 1.60 (1H, m, H-1″), 1.53 (2H, m, H_2_-2″), 1.50 (2H, m, H_2_-6″), 1.37 (4H, m, H_2_-3″, H_2_-5″), 1.32 (3H, br s, H_3_-2), 1.28 (2H, m, H_2_-4″); ^13^C NMR (MeOD; 150 MHz ): δ 80.32 (C-1), 18.03 (C-1′, 2), 138.10 (C-2′), 123.52 (C-3′), 33.65 (C-4′), 116.42 (C-5′), 133.84 (C-6′), 33.49 (C-1″), 30.85 (C-2″), 30.33 (C-3″), 28.03 (C-4″), 30.46 (C-5″), 30.20 (C-6″), 76.13 (C-1‴), 71.05 (C-2‴), 65.01 (C-3‴), 63.87 (C-4‴), 63.03 (C-5‴); ESIMS *m*/*z* (rel..int.): 339 [M + H]^+^ (C_19_H_31_O_5_) (1.3), 259 (16.3), 255 (5.8); HR-ESIMS *m*/*z* (rel. int.): 339.2167 (calcd. 339.2171 for C_19_H_31_O_5_).

Guaiacol-*O*-β-d-arabinopyranoside (**5**)

Semi-solid; IR *ν_max_* (KBr): 3415, 3380, 2934, 2847, 1560, 1506, 1455, 1419, 1334, 1226, 1120, 1065, 1039 cm^−1^; ^1^H NMR (DMSO-*d*_6_): δ 7.01 (1H, dd, *J* = 2.8, 9.3 Hz, H-3), 6.44 (1H, m, H-4), 6.35 (1H, m, H-5), 6.31 (1H, dd, *J* = 2.1, 10.0 Hz, H-6), 4.91 (1H, d, *J* = 7.0 Hz, H-1′), 4.26 ((1H, m, H-2′), 3.76 (1H, d, *J* = 8.0 Hz, H_2_-5′a), 3.73 (1H, d, *J* = 8.0 Hz, H_2_-5′b), 3.58 (1H, m, H-3′), 3.41 (1H, m, H-4′), 3.31 (3H, br s OMe); ^13^C NMR (DMSO-d_6_): δ154.84 (C-1), 154.39 (C-2), 134.46 (C-3), 129.87 (C-4), 131.66 (C-5), 123.79 (C-6), 57.05 (OMe), 105.74 (C-1′), 78.89 (C-2′), 75.70 (C-3′), 72.14 (C-4′), 63.27 (C-5′); ESIMS *m*/*z* (rel. int.): 257 [M + H]^+^ (C_12_H_17_O_6_). (Compare the data with literature [25]).

*n*-Tetradecanyl oleate (**6**)

Semi-solid; IR *ν_max_* (KBr): 2924, 2854, 1721, 1635, 1450, 1376, 1231, 1178, 1049, 985, 895, 721 cm^−1^; ^1^H NMR (CDCl_3_): δ 5.36 (1H, m, H-9), 5.32 (1H, m, H-10), 3.64 (2H, t, *J* = 7.0 Hz, H_2_-1′), 2.77 (2H, t, *J* = 7.0 Hz, H_2_-2), 2.34 (2H, m, H_2_-8), 2.05 (2H, m, H_2_-11), 2.02 (2H, m, H_2_-3), 1.61 (2H, m, CH_2_), 1.55 (2H, m, CH_2_), 1.36 (20 H, m, 10 × CH_2_), 1.30 (12H, br s, 6 × CH_2_), 1.27 (8H, br s 4 × CH_2_), 0.86 (3H, t, *J* = 6.5 Hz, Me-18), 0.82 (3H, t, *J* = 6.3 Hz, Me-14′); ^13^C NMR (CDCl_3_): δ 178.59 (C-1), 130.09 (C-9), 127.99 (C-10), 63.32 (C-1′), 33.84 (C-2), 32.73 (C-8), 32.54 (C-11), 31.88 (CH_2_ ), 31.50 (CH_2_ ), 29.74 (CH_2_ ), 29.63 (CH_2_ ), 29.56 (CH_2_ ), 29.47 (CH_2_ ), 29.37 (CH_2_ ), 29.32 (CH_2_ ), 29.22 (4 × CH_2_ ), 29.12 (2 × CH_2_ ), 29.06 (CH_2_ ), 29.02 (CH_2_ ), 27.18 (CH_2_ ), 25.71 (CH_2_ ), 25.71(CH_2_ ), 25.62 (CH_2_ ), 24.87 (CH_2_ ), 24.61(CH_2_ ), 22.64 (CH_2_ ), 22.54 (CH_2_ ), 14.08 (C-18), 14.03 (C-14′); ESIMS *m/z* (re. int.): 479 [M + H]^+^ (C_32_H_63_O_2_) (27.2), 281 (10.3), 265 (11.6); (Compare the data with literature [26]).

Oleiyl-*O*-β-d-xyloside (**7**)

Semi-solid; IR *ν_max_* (KBr): 3410, 3350, 2916, 2849, 1736, 1648, 1461, 1376, 1242, 1167, 1062, 867 cm^−1^; ^1^H NMR (CDCl_3_): δ 5.37 (1H, m, H-9), 5.32 (1H, m, H-10), 5.09 (1H, d, *J* = 7.5 Hz, H-1′), 4.33 (1H, m, H-3′), 4.26 (1H, d, *J* = 5.5 Hz, H_2_-5′a), 4.15 (1H, d, *J* = 6.0 Hz, H_2_-5′b), 4.05 (1H, dd, *J* = 7.5, 6.5 Hz, H-2′), 3.73 (1H, m, H-4′), 2.76 (1H, t, *J* = 6.5 Hz, H_2_-2), 2.33 (2H, m, H_2_-8), 2.04 (1H, m, H_2_-10), 1.68 (2H, m, CH_2_), 1.34 (6H, br s, 3 × CH_2_), 1.25 (14H, br s, 7 × CH_3_), 0.88 (3H, t, *J* = 6.5 Hz, Me-18); ^13^C NMR (CDCl_3_): δ 173.37 (C-1), 34.06 (C-2), 29.66 (C-3), 29.62 (C-4), 29.58 (C-5), 29.44 (C-6), 29.32 (C-7), 31.89 (C-8), 129.96 (C-9), 128.06 (C-10), 31.50 (C-11), 29.32 (C-12), 29.28 (C-13), 29.07 (C-14), 27.17 (C-15), 24.86 (C-16), 22.66 (C-17), 14.06 (C-18), 103.14 (C-1′), 65.02 (C-2′), 73.13 (C-3′), 61.49 (C-4′), 62.04 (C-5′); ESIMS *m*/*z* (rel. int.) 415 [M + H]^+^ (C_23_H_43_O_6_), (7.1), 281 (9.3), 265 (5.1). (Compare the data with literature [27]).

*n*-Octadec-9,12-dienoyl-*O*-β-d-arabinopyranoside (**8**)

Gummy; IR *ν_max_* (KBr): 3415, 3381, 3277, 2928, 2866, 1721, 1635, 1453, 1388, 1227, 1123, 831, 721 cm^−1^; ^1^H NMR (MeOD): δ 6.29 (1H, m, H-10), 5.89 (1H, m, H-12), 5.40 (1H, m, H-9), 5.13 (1H, m, H-13), 5.77 (1H, d, *J* = 7.2 Hz, H-1′), 4.28 (1H, m, H-4′), 4.13 (1H, m, H-3′), 3.85 (1H, m, H-2′), 3.52 (2H, dd, *J* = 6.5, 7.0 Hz, H_2_-5′), 2.26 (2H, t, *J* = 7.5, Hz, H_2_-2), 2.10 (2H, m, H_-_11), 2.06 (2H, m, H_2_-8), 1.98 (2H, m, H_2_-14), 1.60 (2H, m, CH_2_), 1.51 (2H, m, CH_2_), 1.32 (10H, br s, 5 × CH_2_), 1.28 (2H, m, CH_2_),0.89 (3H, t, *J* = 6.2 Hz, Me-18); ^13^C NMR (MeOD): δ 171.06 (C-1), 37.85, 30.68 (C-2), 30.57 (C-3), 30.52 (C-4), 30.37 (C-5), 30.17 (C-6), 28.49 (C-7), 34.94 (C-8), 129.86 (C-9), 151.86 (C-10), 36.43 (C-11), 134.06 (C-12), 116.61 (C-13), 33.68 (C-14), 26.91 (C-15), 24.98 (C-16), 23.17 (C-17), 14.40 (C-18), 108.40 (C-1′), 72.48 (C-2′), 73.93 (C-3′), 64.02 (C-4′), 63.03 (C-5′); ESIMS *m/z* (rel. int.): 412 [M + H]^+^ (C_23_H_40_O_6_) (5.8), 279 (25.3). (Compare the data with literature [28]).

Linolenyl-*O*-β-d-arabinofuranoside (**9**)

Semisolid; IR *ν_max_* (KBr):3421, 3375, 2928, 2857, 1721, 1645, 1453, 1368, 1247, 1053, 721 cm^−1^; ^1^H NMR (CDCl_3_): δ 5.51 (1H, d, J=7.5 Hz, H-1′), 4.18 (1H, m, H-4′), 3.88 (1H, m, H-3′), 3.62 (1H, m, H-2′), 3.63 (2H, dd, *J*=6.5, 6.5 Hz, H2-5′), 5.49 (1H, m, H-12), 5.47 (1H, m, H-13), 5.41 ((1H, m, H-10), 5.39 (1H, m, H-15), 5.36 (1H, m, H-9), 5.33 (1H, m, H-16), 2.33 (2H, t, *J* = 7.5 Hz, H_2_-2), 2.05 (2H, m, H_2_-11), 2.03 ((2H, m, H_2_-14), 2.01 (2H, m, H_2_-8), 1.80 (2H, m, H_2_-17), 1.65 (2H, m, CH_2_), 1.55 (2H, m, CH_2_), 1.30 (6H, br s 3 CH_2_), 0.89 (3H, t, *J*=6.0 Hz, Me-18); ^13^C NMR (CDCl_3_):δ 170.16 (C-1), 36.80 (C-2), 29.57 (C-3), 29.33 (C-4), 29.13 (C-5), 29.07(C-6), 22.18 (C-7), 25.63 (C-8), 128.08 (C-9), 31.50 (C-10), 34.12 (C-11), 143.02 (C-12), 130.22 (C-13), 33.65(C-14), 122.47 (C-15), 117.13 (C-16),22.65 (C-17), 14.04 (C-18), 109.89 (C-1′), 70.45 (C-2′), 65.16 (C-3′), 85.06 (C-4′), 62.99 (C-1′); ESIMS *m*/*z* (re lint.): 411 [M+H]^+^ (C_23_H_38_O_6_), (15.2), 277 (29.3), 261(22.8). (Compare the data with literature [29]).

Glyceryl-1,3-dipalmito-2-olein (**10**)

Liquid, R_f_ 0.36 (CHCl_3_:MeOH); IRν_max_ (KBr): 2923, 2853, 1735, 1722, 1635, 1460, 1373, 1241, 1166, 1070; ^1^H NMR (CDCl_3_): δ 5.35 (1H, m, H-9″), 5.31 (1H, m, H-10″), 4.28 (1H, m, H-2), 4.13 (2H, m, H_2_-1), 4.05 (2H, m, H_2_-3), 2.79 (2H, m, H_2_-11′), 2.32 (2H, t, *J* = 7.0 Hz, H_2_-2′), 2.29 (2H, t, *J* = 7.0 Hz, H_2_-2″), 2.05 (2H, m, H_2_-2‴), 2.03 (2H, m, H_2_-8″), 1.61 (2H, m, CH_2_), 1.51 (6H, m, 3 × CH_2_), 1.29 (40 H, br s, 20 × CH_2_), 1.25 (26H, m, 13 × CH_2_), 0.90 (3H, t, *J* = 6.1 Hz, Me-18″), 0.87 (3H, t, *J* = 6.3 Hz, Me-16′), 0.84 (3H, t, *J* = 6.1 Hz, Me-16‴); ^13^C NMR (CDCl_3_): δ 173.51 (C-1′), 173.30 (C-1″), 173.06 (C-1‴), 128.20 (C-10″), 127.76 (C-9″), 39.36 (C-2′), 37.42 (C-2″), 37.27 (C-2‴), 34.27 (CH_2_), 34.09 (CH_2_), 34.03 (CH_2_), 33.66 (CH_2_), 31.89 (CH_2_), 31.50 (CH_2_), 30.69 (CH_2_), 29.67 (8 × CH_2_), 29.63 (7 × CH_2_), 29.44 (CH_2_), 29.32 (CH_2_), 29.23 (CH_2_), 29.15 (CH_2_), 29.10 (CH_2_), 28.62 (CH_2_), 27.95 (CH_2_), 27.72 (CH_2_), 27.18 (CH_2_), 25.61 (CH_2_), 24.64 (CH_2_), 24.77 (CH_2_), 24.46 (CH_2_), 23.07 (CH_2_), 22.66 (CH_2_), 22.54 (CH_2_), 21.96 (CH_2_), 14.08 (Me-16′), 14.03 (Me-18″), 11.95 (Me-16‴); ESIMS *m*/*z* (rel. int): 833 [M + H]^+^ (C_53_H_100_O_6_) (1.5), 282 (100), 256 (1.8). (Compare the data with literature [30]).

^1^H and ^13^CNMR spectra of these compounds are available in the Supplementary Materials.

### 3.4. Antioxidant Activity

#### 3.4.1. Chemicals and Instruments in Antioxidant Activity

All the chemicals, reagents and the solvents used in the assay protocols were of analytical grade. Ascorbic acid, tocopherol, BHT, BHA, water, phosphate buffer, potassium ferricyanide, trichloroacetic acid, ferric chloride DPPH, DMSO, sulfanilamide and naphthylethylenediamine for Griess reagent, sodium hydroxideand sodium citrate were obtained from Sigma Aldrich, (St. Louis, MO, USA). Also, all other chemicals were purchased from Sigma Aldrich, USA. Spectrophotometer was used from Thermo Scientific, Multiskan GO Spectrophotometer (Sl. No. 1510033456).

#### 3.4.2. Free Radical Scavenging Activity

Antioxidant activity of the different constituents (**1**–**4**), based on the scavenging activity of the stable 1,1-diphenyl-2-picrylhydrazyl (DPPH) free radical, was examined by the method described by Katerere and Eloff, [18]. A wide range of concentrations (10, 25, 50 and 100 µg/mL) of the compounds to be tested (0.2 mL; tocopherol) were taken in different test tubes with 4 mL of a 0.006% MeOH solution of DPPH. Water (0.2 mL) was taken as a standard. Absorbance at 517 nm was determined after 30 min. Radical scavenging activity was measured in terms of the inhibition percentage, and was calculated using Equation (1).

% Radical scavenging activity = [*A*_0_ − *A*_1_] × 100 (1)

where *A*_0_ is the absorbance (control) and *A*_1_ is the absorbance (compounds/standard).

#### 3.4.3. Reducing Power

The reducing power of the ginseng compounds was determined according to the method of Oyaizu [31]. Different compounds of concentrations 10, 25, 50 and 100 µg were dissolved in 1 mL of distilled water and mixed with phosphate buffer (2.5 mL, 0.2 M/L, pH 6.6) and potassium ferricyanide [K3Fe (CN)6] (2.5 mL, 1%). The mixture was kept for incubation at 50 °C for 20 min. Trichloroacetic acid (10%) was added (2.5 mL) to the mixture, which was then centrifuged at 1000 rpm for 10 min. The upper layer of the centrifuged solution (2.5 mL) was mixed with distilled water (2.5 mL) and FeCl_3_ (0.5 mL, 0.1%) and the absorbance was optimized at 700 nm. The enhanced absorbance of the reaction mixture indicated improved reducing power. All analyses were done in triplicate, and averaged. The reducing power was calculated using Equation (2).

(2) $$\mathrm{FRAP}\;\mathrm{value}\;\mathrm{of}\;\mathrm{sample}\;(µM)\;=\;\frac{\mathrm{Abs}\left(\mathrm{sample}\right)\;\times \;\mathrm{FRAP}\;\mathrm{value}\;\mathrm{of}\;\mathrm{Std}\;(µM)}{\mathrm{Abs}\;\left(\mathrm{Std}\right)}$$

#### 3.4.4. Nitric Oxide Scavenging Activity

Sodium nitroprusside in an aqueous solution at a physiological pH generates nitric oxide, which interacts with oxygen to produce nitric ions that can be estimated using Griess reagent. The complex formed during the diazotization of nitrite with sulphanilamide and the subsequent coupling with naphthylethylenediamine (Griess reagent) was read at 546 nm and the methods of Marcocci [32]. Different concentrations of samples (10, 25, 50 and 100 µg/mL) were prepared and added in sodium nitroprusside with a phosphate buffer. The above prepared reaction mixture was incubated at room temperature for 30 min. Then 50 µL of incubated reaction mixture was transferred to another microplate followed by the addition of Griess reagent, and absorbance was recorded at 546 nm. The percentage nitrite radical scavenging activity of the ethanol extracts and gallic acid were calculated using Equation (3).


Nitic Oxide Scavenge (%) = A _control_ − A _test_/A _control_ × 100 (3)

Percentage nitrite radical scavenging activity: Where = absorbance of control sample and = absorbance in the presence of the samples of extracts or standards.

#### 3.4.5. Evaluation of Antioxidant Capacity by Phosphomolybdenum Method

The total antioxidant capacity of the compounds (**1**–**4**) was evaluated by the method of Prieto [33] based on reduction of Mo (VI) to Mo (V) in acidic conditions resulting into development of a greenish complex of phosphate and Mo (V). The details of sample, reagents and standard were given in the literature [34].

## 4. Conclusions

*Dendrodropanax morbifera* is an endemic species in Korea, and is found in the southern parts of Korea. The *D. morbifera* have been reported as several classes of compounds, and many biological activities were also found in this plant’s constituents and extracts. Four new compounds (**1**–**4**) have been isolated in this study, and we evaluated its antioxidant exercise as a radical scavenging effect, reducing power, phosphomolybdenum and the nitric oxide activity. The results showed the compounds (**1** and **2**) have good antioxidant activity in comparison with compounds (**3** and **4**). The developed path has been verified, and found to be useful in the investigation of active constituents in natural medicines. Further studies are needed to investigate more isolation work of novel constituents from other Dendropanax species that show strong activities as above, and also other activities.

## Figures and Tables

**Table 1:** Antioxidant activities of the compounds (C-1 to C-4) as measured by Nitric Oxide scavenging power, Ferric reducing antioxidant power assay (FRAP) and 1,1-diphenyl-2-picrylhydrazyl (DPPH) radical scavenging assay. Compound **1** and **2** exhibited significant activities at all the concentrations.

Parameter	Compound	10 µg	25 µg	50 µg	100 µg
**Nitric Oxide**	C-1	17.62	18.33	38.55	40.38
	C-2	13.52	14.28	36.82	37.64
	C-3	9.19	10.62	20.61	28.34
	C-4	8.46	10.11	18.55	25.39
**FRAP**	C-1	22.36	25.34	78.42	79.68
	C-2	19.55	20.39	65.43	69.77
	C-3	10.08	10.34	42.11	54.33
	C-4	8.67	8.33	39.33	50.22
**DPPH**	C-1	11.97	13.24	65.73	69.84
	C-2	8.72	10.02	11.32	12.62
	C-3	4.46	5.64	22.15	28.25
	C-4	3.54	3.51	21.15	24.67

**Table 2:** Antioxidant capacity of compounds C-1 to C-4 by the phosphomolybdenum method.

Compounds	Antioxidant Capacity (%) as Equivalent to α-Tocopherol (mg/g)
C-1	15.31
C-2	13.20
C-3	6.70
C-4	5.63

## Supplementary Materials

The supplementary materials are available online.

## References

[1] Han S.H., Jung Y.H., Oh M.H., Ko M.H., Oh Y.S., Koh S.C., Kim M.H., Oh M.Y. (1998). Phytogenetic relationship of the *Dendropanax morfera* and *D. trifidus* based on PCR-RAPD. Korean J. Genet..

[2] Bae K.H. (2000). The Medicinal Plants of Korea.

[3] Park B.Y., Min B.S., Oh S.R., Kim J.H., Kim T.J., Kim D.H., Bae K.H., Lee H.Y. (2004). Isolation and anticomplement activity of compounds from Dendropanx morbifera. J. Ethnopharmacol..

[4] Setzer W.N., Green T.J., Whitaker K.W., Moriarity D.M., Yancey C.A., Lawton R.O., Bates R.B. (1995). A cytotoxic diacetylene from Dendropanax arboreus. Planta Med..

[5] Bernart M.W., Balacschak M.S., Alexander M.R., Shoemaker R.H., Boyd M.R. (1996). Cytotoxic falcarinol oxylipins from Dendropanax arboreus. J. Nat. Prod..

[6] Kawazu K., Noguchi H., Fujishita K., Iwasa J. (1973). Two new antifungal compounds from Dendropanax trifidus. Tetrahedron Lett..

[7] Oka K., Saito F., Yasuhara T., Sugimoto A. (1999). The allergens of Dendropanax trifidus Makino and Fatsis japonica Decne. Et Planch and evaluation of cross-reactions with other plants of the Araliaceae family. Contact Dermat..

[8] Oka K., Fumio S., Tahashi Y., Akiko S. (1997). The major allergen of Dendropanax trifidus Makino. Contact Dermat..

[9] Kobayashi A., Tagawa K., Yamashita K. (1977). Synthesis of dendrotrifidol and its naturally occurring analogs. Agric. Biol. Chem..

[10] Tori M., Tori T., Tachibna K., Yamada S., Tsuyuki T., Takahashi T. (1977). The backbone rearrangement of 3β, 4β-epoxyfriedelanr and thje synthesis of dendropanaxide. Bull Chem. Soc. Jpn..

[11] Gil R.R., Ulubelen A., Chai H.B., Pezzuto J.M., Cordell G.A. (1994). Biologically active compounds from the Euphorbiaceae; 2. Two triterpenoids of Euphobia cyparissias. Planta Med..

[12] Chung I.M., Kim Y.C., Ali M., Kim S.H., Park I., Kim E.H., Yang Y.S., Park H.Y., Son E.U., Ahmad A. (2014). Triterpene glycosides from red ginseng marc and their anti-inflammatory activities. Bioorganic Med. Chem. Lett..

[13] Ahmad A., Kim S.H., Ali A., Park I., Kim J.S., Kim E.H., Lim J.L., Kim S.K., Chung I.M. (2013). New chemical constituents from *Oryza sativa* and their algicidal activities against blue-green algae. J. Agric. Food Chem..

[14] Chung I.M., Song H.K., Kim S.J., Moon H. (2011). Anticomplement activity of polyacetylenes from leaves of Dendropanax morboifera Leveille. Phytothe. Res..

[15] Moon H.I. (2011). Antidiabetic effects of dendropanoxide from leaves of Dendropanax morbifera Leveille in normal and streptozotocin-induced diabetic rats. Hum. Exp. Toxicol..

[16] Hyun T.K., Ko Y.J., Kim E.H., Chung I.M., Kim J.S. (2015). Anti-inflammatory activity and phenolic composition of Dendropanax morbifera leaf extracts. Ind. Crops Prod..

[17] Choi J.U., Kim D.W., Park S.E., Lee H.J., Kim K.M., Kim K.J., Kim M.K., Kim S.J., Kim S. (2015). Anti-thrombotic effect of rutin isolated from Dendropanax morbifera Leveille. J. Biosci. Bioeng..

[18] Katerere D.R., Eloff J.N. (2005). Antibacterial and antioxidant activity of Sutherlandia frutescens (Fabaceae), a reputed Anti-HIV/AIDS phytomedicine. Phytother. Res..

[19] Li X.M., Li X.L., Zhou A.G. (2007). Evaluation of antioxidant activity of the polysaccharides extracted from Lycium barbarum fruits in vitro. Eur. Polym. J..

[20] Chang L.W., Yen W.J., Huang S.C., Duh P.D. (2002). Antioxidant activity of sesame coat. Food Chem..

[21] Chung Y.C., Chang C.T., Chao W.W., Lin C.F., Chu S.T. (2002). Antioxidative activity and safety of the 50 ethanolic extract from red bean fermented by Bacillus subtilis IMR-NK1. J. Agric. Food Chem..

[22] Okuda T., Kimura Y., Yoshida T., Hatano T., Okuda H., Arichi S. (1983). Studies on the activities of tannins and related compounds from medicinal plants and drugs. I Inhibitory effects on lipid peroxidation in mitochondria and microsomes of liver. Chem. Pharm. Bull..

[23] Tanaka M., Kuie C.W., Nagashima Y., Taguchi T. (1988). Application of antioxidative maillard reaction products from histidine and glucose to sardine products. Nippon Suisan Gakkaishi.

[24] Oktay M., Culcin I., Kufrevioglu O.I. (2003). Determination of in vitro antioxidant activity of fennel (Foeniculum vulgare) seed extracts. Lebensm. Wiss. Technol..

[25] Francisco A.T.B., Juan C.E. (2001). Phenolic compounds and related enzymes as determinants of quality in fruits and vegetables. J. Sci. Food Agric..

[26] Ahmad A., Yoon J.Y., Chung I.M. (2013). Chemical constituents from the straw of Oryza sativa. Asian J. Chem..

[27] Hori R. (1959). Studies on carbohydrate derivatives VI. Studies on the surface tension of their aqueous solution. Yakugaku Zasshi.

[28] Priyanka B., Vidhu A., Mohd A. (2017). New fatty acid glycosides from the seeds of Cicer arietinum. J. Pharmacogn. Phytochem..

[29] Yamada H., Ra K.S., Kiyohara H., Cyong J.C., Yang Y.C., Otsuka Y. (1988). Characterization of anti-complementary neutral polysaccharides from the roots of Bupleurum falcatum. Phytochemistry.

[30] Chung I.M., Kim H.J., Yhn Y.S., Ahmad A. (2013). Glycerol derivatives of fatty acids from the fruits of Lycium chinense. Asian J. Chem..

[31] Oyaizu M. (1986). Studies on product of browning reaction prepared from glucose amine. Jpn. J. Nutr..

[32] Marcocci L., Maguire J.J., Droy-Lafix M.T., Packer L. (1994). The nitric oxide scavenging property of Ginkgo biloba extracts EGb 761. Biochem. Biophys. Res. Commun..

[33] Prieto P., Pineda M., Aguilar M. (1999). Spectrophotometric quantification of antioxidant capacity through the formation of a phosphomolybdenum complex: Specific application to the determination of vitamin E. Anal. Biochem..

[34] Chung I.M., Ali M., Nagella P., Yu B.R., Kim S.H., Ahmad A. (2014). New polyglucopyranosyl and polyarabinopyranosyl of fatty acid derivatives from the fruits of Lycium chinense and itsantioxidant activity. Food Chem..

