# Supplementary material for: New Chemical Constituents from the Bark of Dendropanax morbifera Leveille and Their Evaluation of Antioxidant Activities
Source: Molecules. 2019 Nov 1;24(21):3967. doi: 10.3390/molecules24213967 (PMC6865017; doi:10.3390/molecules24213967)
Supplement: Supplementary file 1 [file molecules-24-03967-s001.pdf]

Compound 1

^{1}H NMR

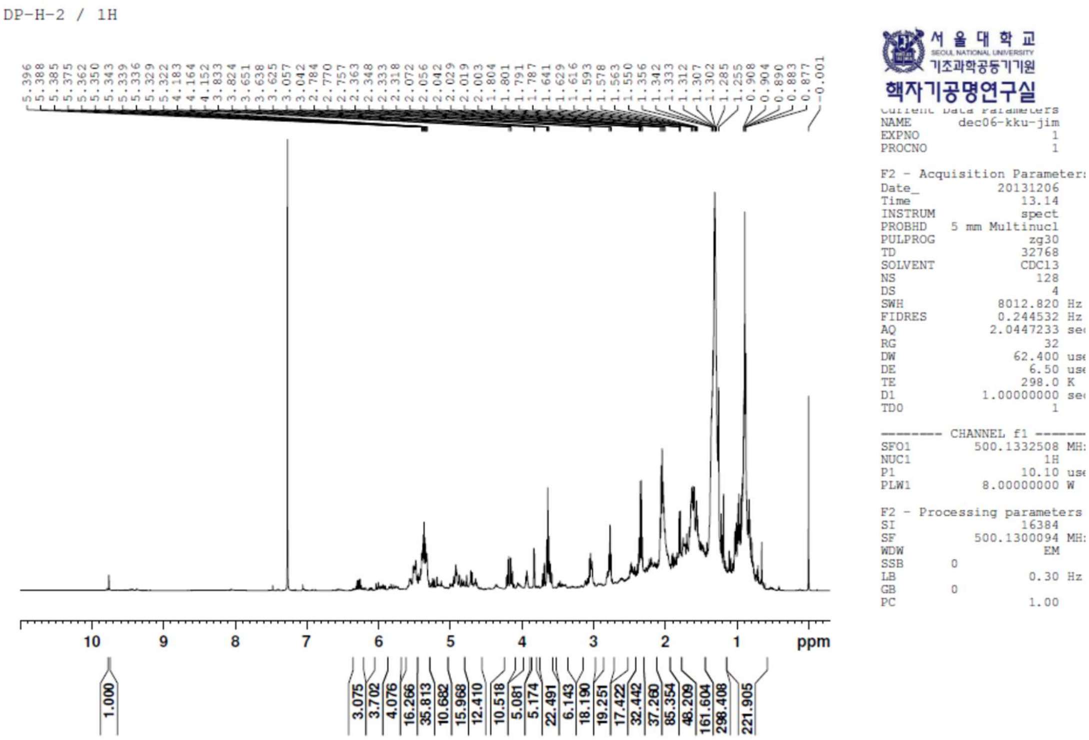

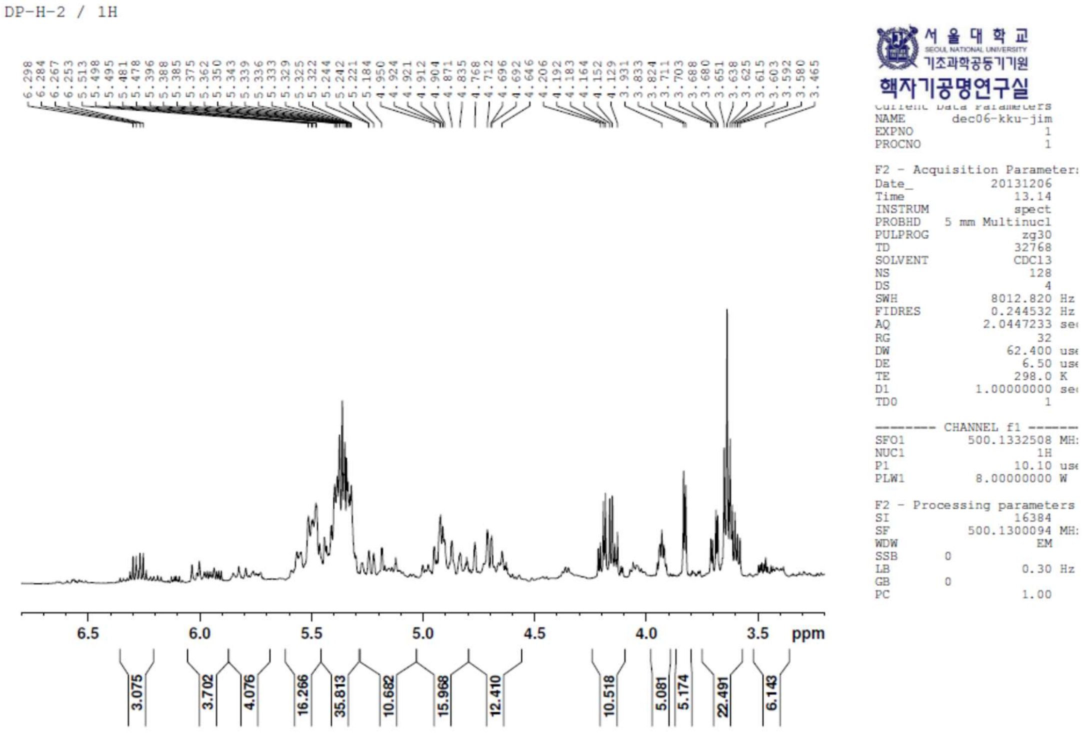
DP-H-2 / 1H
서울대학교
SEOUL NATIONAL UNIVERSITY
기초과학공동기기원
핵자기공명연구실
Current Data Parameters
NAME dec06-kku-jim
EXPNO 1
PROCNO 1
F2 - Acquisition Parameters
Date_ 20131206
Time 13.14
INSTRUM spect
PROBHD 5 mm Multinucl
PULPROG zg30
TD 32768
SOLVENT CDCl3
NS 128
DS 4
SWH 8012.820 Hz
FIDRES 0.244532 Hz
AQ 2.0447233 sec
RG 32
DW 62.400 usec
DE 6.50 usec
TE 298.0 K
D1 1.00000000 sec
TD0 1
CHANNEL f1
SFO1 500.1332508 MHz
NUC1 1H
P1 10.10 usec
PLW1 8.00000000 W
F2 - Processing parameters
SI 16384
SF 500.1300094 MHz
WDW EM
SSB 0
LB 0.30 Hz
GB 0
PC 1.00
6.5 6.0 5.5 5.0 4.5 4.0 3.5 ppm

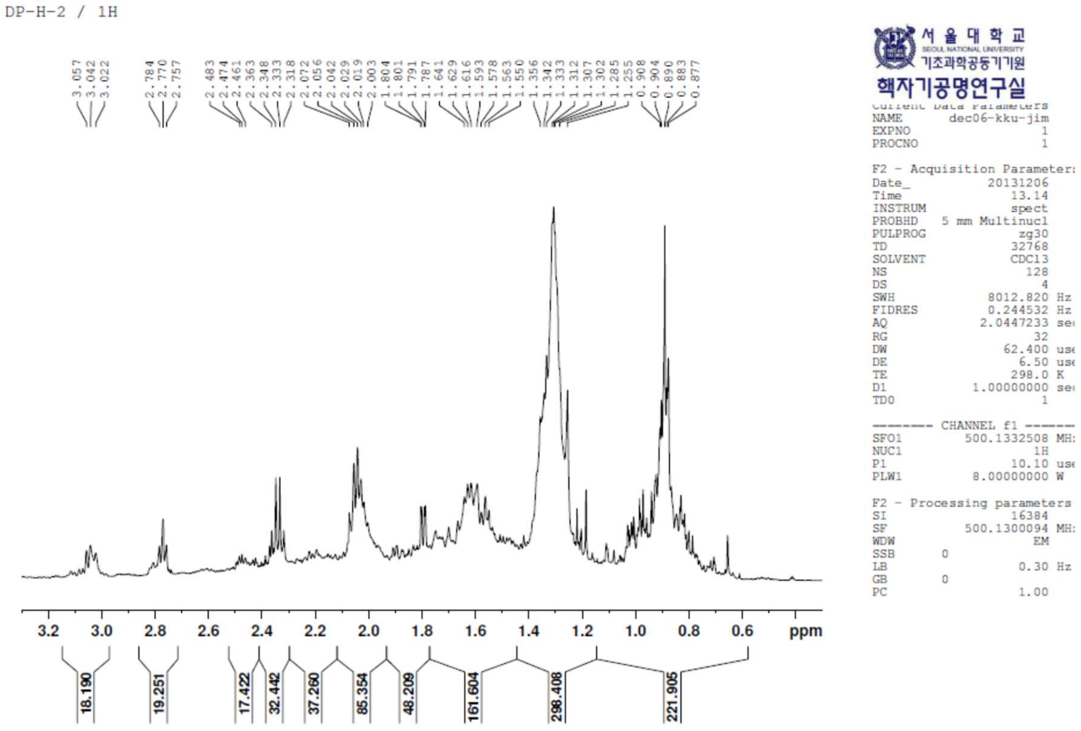
DP-H-2 / 1H
서울대학교
SEOUL NATIONAL UNIVERSITY
기초과학공동기기원
핵자기공명연구실
Current Data Parameters
NAME dec06-kku-jim
EXPNO 1
PROCNO 1
F2 - Acquisition Parameters
Date_ 20131206
Time 13.14
INSTRUM spect
PROBHD 5 mm Multinucl
PULPROG zg30
TD 32768
SOLVENT CDCl3
NS 128
DS 4
SWH 8012.820 Hz
FIDRES 0.244532 Hz
AQ 2.0447233 sec
RG 32
DW 62.400 usec
DE 6.50 usec
TE 298.0 K
D1 1.00000000 sec
TD0 1
CHANNEL f1
SFO1 500.1332508 MHz
NUC1 1H
P1 10.10 usec
PLW1 8.00000000 W
F2 - Processing parameters
SI 16384
SF 500.1300094 MHz
WDW EM
SSB 0
LB 0.30 Hz
GB 0
PC 1.00
3.2 3.0 2.8 2.6 2.4 2.2 2.0 1.8 1.6 1.4 1.2 1.0 0.8 0.6 ppm

^{13}C NMR

DP-H-2 / 13C

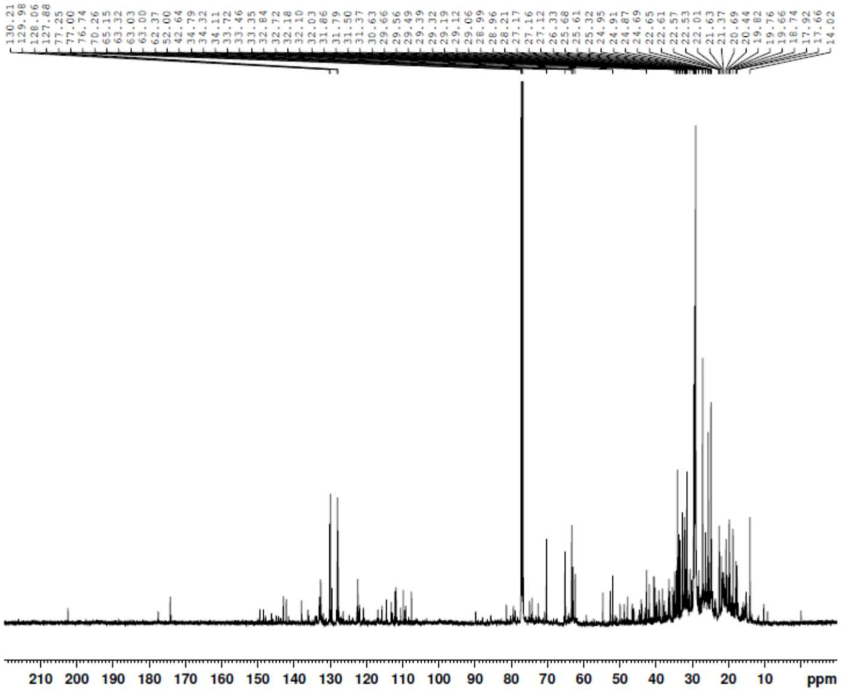

서 울 대 학 교
SEOUL NATIONAL UNIVERSITY
기초과학공동기기원
핵자기공명연구실

```
Current Data Parameters
NAME        dec06-kku-jim
EXPNO                   5
PROCNO                  1

F2 - Acquisition Parameters
Date_            20131209
Time                 5.16
INSTRUM             spect
PROBHD   5 mm Multinucl
PULPROG              zgdc
TD                  32768
SOLVENT             CDCl3
NS                  51200
DS                      4
SWH             29761.904 Hz
FIDRES           0.908261 Hz
AQ              0.5505024 sec
RG                    912
DW                 16.800 usec
DE                   6.50 usec
TE                  298.0 K
D1             2.00000000 sec
D11            0.03000000 sec
TD0                     1

======== CHANNEL f1 ========
SFO1          125.7709936 MHz
NUC1                  13C
P1                  12.00 usec
PLW1         180.00000000 W

======== CHANNEL f2 ========
SFO2          500.1320005 MHz
NUC2                   1H
CPDPRG[2          waltz16
PCPD2               80.00 usec
PLW2           8.00000000 W
PLW12          0.35066000 W

F2 - Processing parameters
SI                  16384
SF            125.7577907 MHz
WDW                    EM
SSB      0
LB                   1.00 Hz
GB       0
PC                   1.40
```

DP-H-2 / 13C

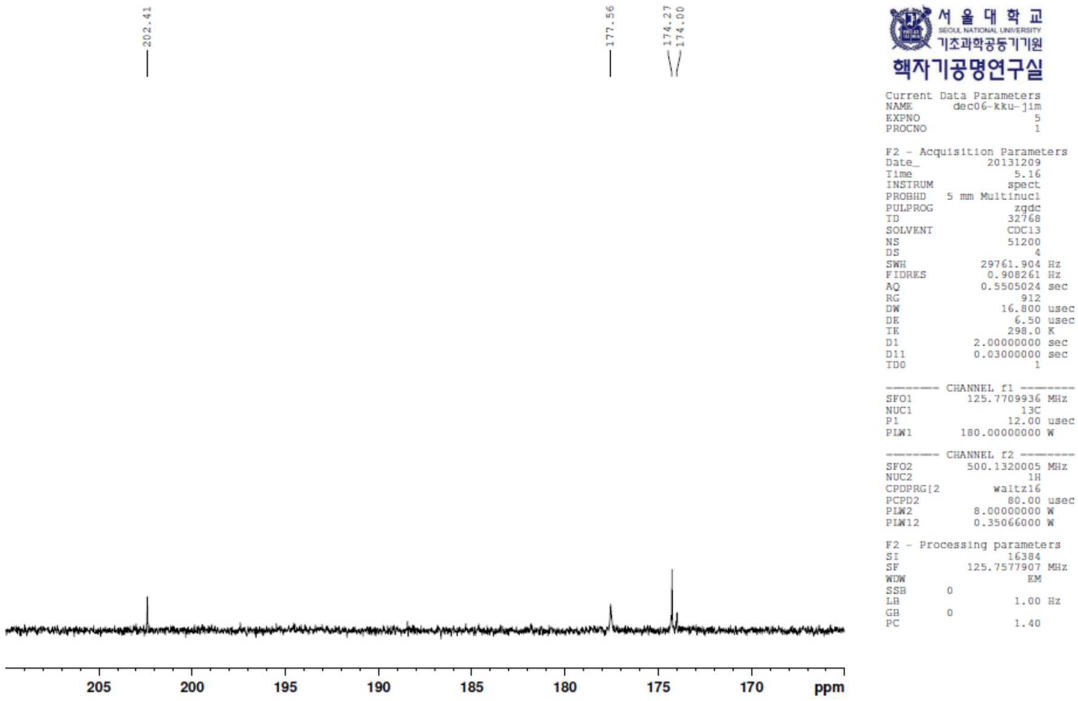

DP-H-2 / 13C

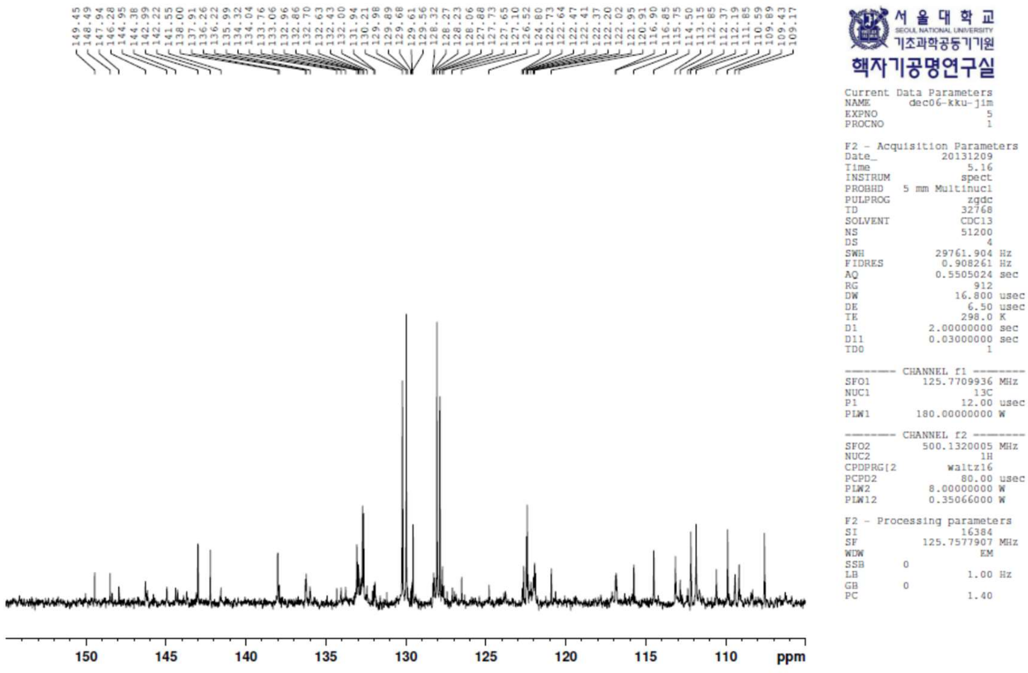

DP-H-2 / 13C

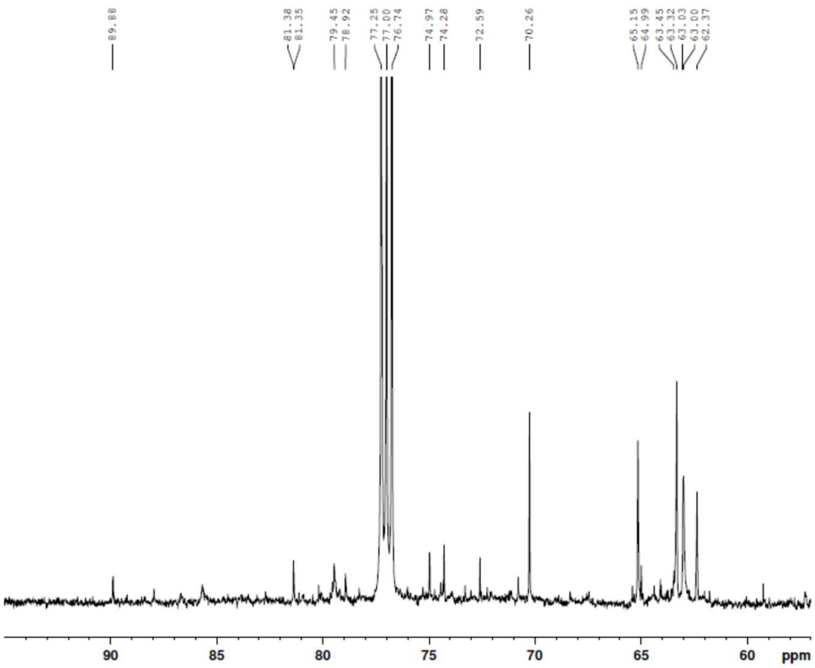

서울대학교
SEOUL NATIONAL UNIVERSITY
기초과학공동기기원
핵자기공명연구실

```
Current Data Parameters
NAME        dec06-kku-jim
EXPNO                   5
PROCNO                  1

F2 - Acquisition Parameters
Date_            20131209
Time                 5.16
INSTRUM             spect
PROBHD   5 mm Multinucl
PULPROG              zgdc
TD                  32768
SOLVENT             CDCl3
NS                  51200
DS                      4
SWH             29761.904 Hz
FIDRES           0.908261 Hz
AQ              0.5505024 sec
RG                    912
DW                 16.800 usec
DE                   6.50 usec
TE                  298.0 K
D1             2.00000000 sec
D11            0.03000000 sec
TD0                     1

======== CHANNEL f1 ========
SFO1          125.7709936 MHz
NUC1                  13C
P1                  12.00 usec
PLW1         180.00000000 W

======== CHANNEL f2 ========
SFO2          500.1320005 MHz
NUC2                   1H
CPDPRG[2          waltz16
PCPD2               80.00 usec
PLW2           8.00000000 W
PLW12          0.35066000 W

F2 - Processing parameters
SI                  16384
SF            125.7577907 MHz
WDW                    EM
SSB      0
LB                   1.00 Hz
GB       0
PC                   1.40
```

DP-H-2 / 13C

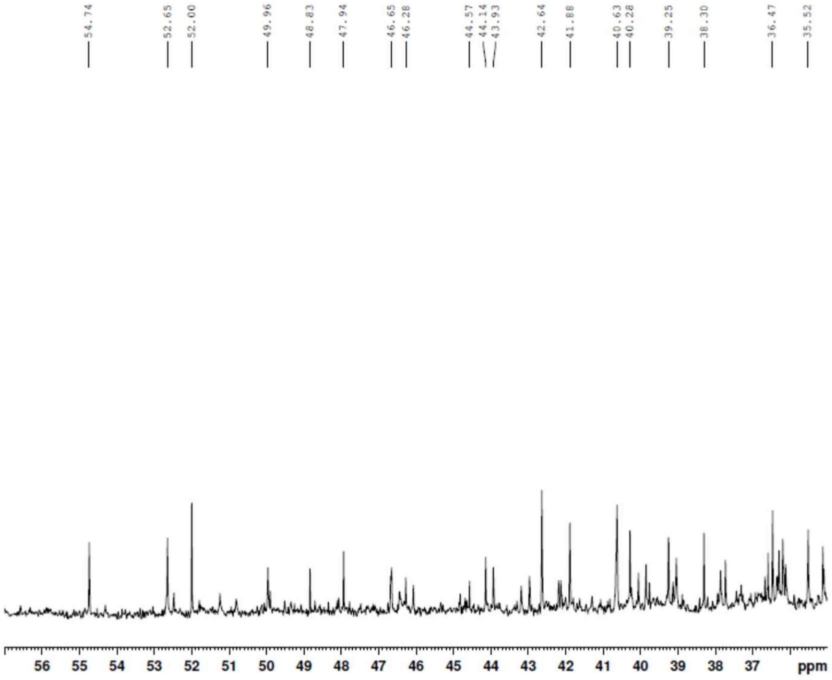

서울대학교
SEOUL NATIONAL UNIVERSITY
기초과학공동기기원
핵자기공명연구실

```
Current Data Parameters
NAME        dec06-kku-jim
EXPNO                   5
PROCNO                  1

F2 - Acquisition Parameters
Date_            20131209
Time                 5.16
INSTRUM             spect
PROBHD   5 mm Multinucl
PULPROG              zgdc
TD                  32768
SOLVENT             CDCl3
NS                  51200
DS                      4
SWH             29761.904 Hz
FIDRES           0.908261 Hz
AQ              0.5505024 sec
RG                    912
DW                 16.800 usec
DE                   6.50 usec
TE                  298.0 K
D1             2.00000000 sec
D11            0.03000000 sec
TD0                     1

======== CHANNEL f1 ========
SFO1          125.7709936 MHz
NUC1                  13C
P1                  12.00 usec
PLW1         180.00000000 W

======== CHANNEL f2 ========
SFO2          500.1320005 MHz
NUC2                   1H
CPDPRG[2          waltz16
PCPD2               80.00 usec
PLW2           8.00000000 W
PLW12          0.35066000 W

F2 - Processing parameters
SI                  16384
SF            125.7577907 MHz
WDW                    EM
SSB      0
LB                   1.00 Hz
GB       0
PC                   1.40
```

DP-H-2 / 13C

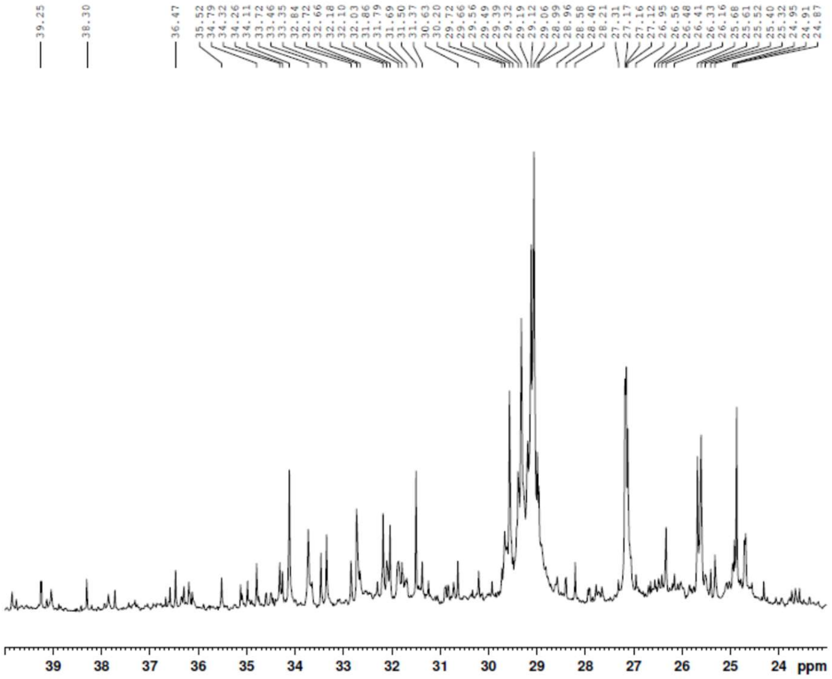

서울대학교
SEOUL NATIONAL UNIVERSITY
기초과학공동기기원
핵자기공명연구실

```
Current Data Parameters
NAME       dec06-kku-jim
EXPNO                  5
PROCNO                 1

F2 - Acquisition Parameters
Date_           20131209
Time                5.16
INSTRUM            spect
PROBHD   5 mm Multinucl
PULPROG             zgdc
TD                 32768
SOLVENT            CDCl3
NS                 51200
DS                     4
SWH            29761.904 Hz
FIDRES          0.908261 Hz
AQ             0.5505024 sec
RG                   912
DW                16.800 usec
DE                  6.50 usec
TE                 298.0 K
D1            2.00000000 sec
D11           0.03000000 sec
TD0                    1

======== CHANNEL f1 ========
SFO1         125.7709936 MHz
NUC1                 13C
P1                 12.00 usec
PLW1        180.00000000 W

======== CHANNEL f2 ========
SFO2         500.1320005 MHz
NUC2                  1H
CPDPRG[2         waltz16
PCPD2              80.00 usec
PLW2          8.00000000 W
PLW12         0.35066000 W

F2 - Processing parameters
SI                 16384
SF           125.7577907 MHz
WDW                   EM
SSB      0
LB                  1.00 Hz
GB       0
PC                  1.40
```

DP-H-2 / 13C

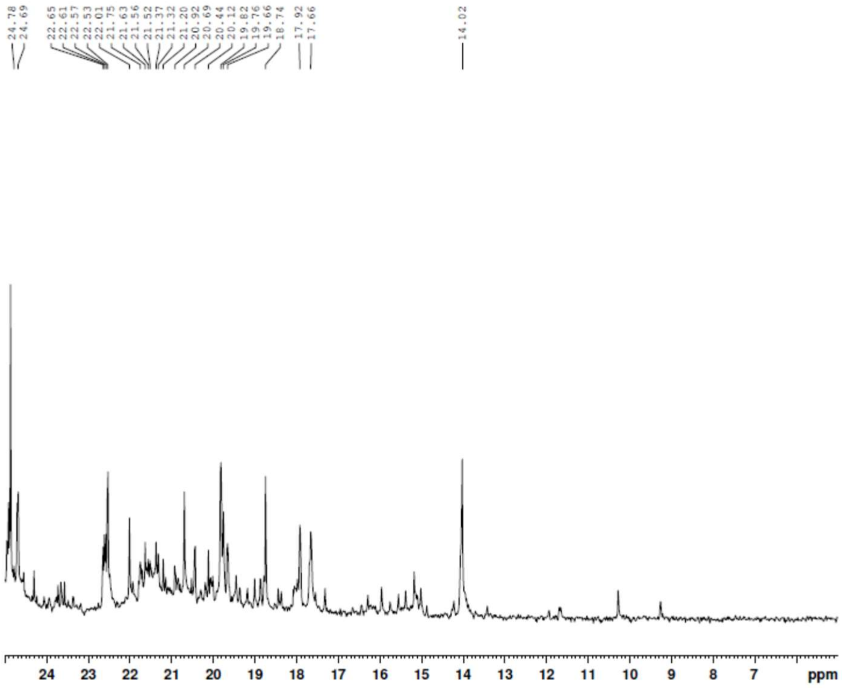

서울대학교
SEOUL NATIONAL UNIVERSITY
기초과학공동기기원
핵자기공명연구실

```
Current Data Parameters
NAME       dec06-kku-jim
EXPNO                  5
PROCNO                 1

F2 - Acquisition Parameters
Date_           20131209
Time                5.16
INSTRUM            spect
PROBHD   5 mm Multinucl
PULPROG             zgdc
TD                 32768
SOLVENT            CDCl3
NS                 51200
DS                     4
SWH            29761.904 Hz
FIDRES          0.908261 Hz
AQ             0.5505024 sec
RG                   912
DW                16.800 usec
DE                  6.50 usec
TE                 298.0 K
D1            2.00000000 sec
D11           0.03000000 sec
TD0                    1

======== CHANNEL f1 ========
SFO1         125.7709936 MHz
NUC1                 13C
P1                 12.00 usec
PLW1        180.00000000 W

======== CHANNEL f2 ========
SFO2         500.1320005 MHz
NUC2                  1H
CPDPRG[2         waltz16
PCPD2              80.00 usec
PLW2          8.00000000 W
PLW12         0.35066000 W

F2 - Processing parameters
SI                 16384
SF           125.7577907 MHz
WDW                   EM
SSB      0
LB                  1.00 Hz
GB       0
PC                  1.40
```

DP-H-2 / HMBC

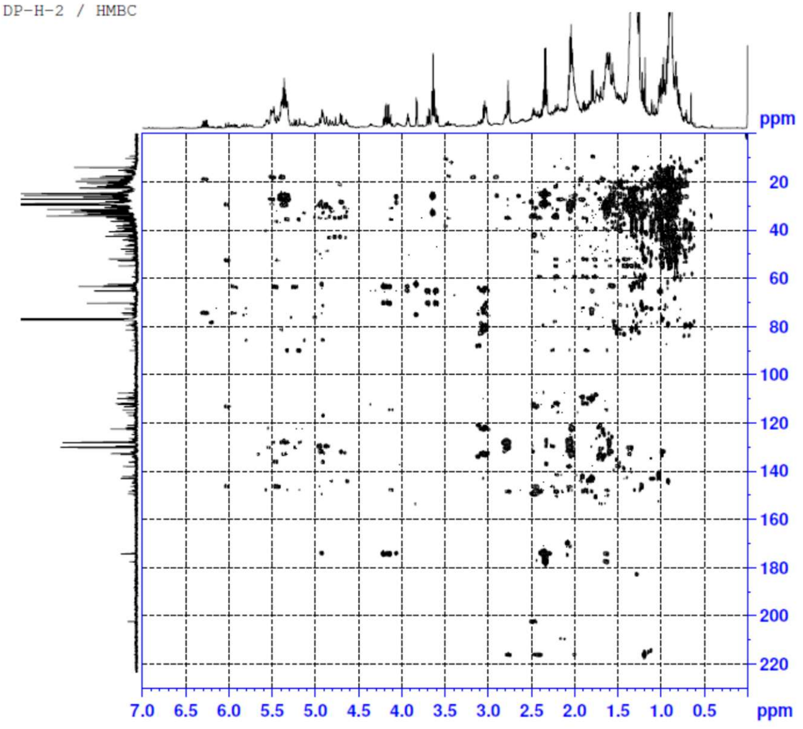

서 울 대 학 교
SEOUL NATIONAL UNIVERSITY
기초과학공동기기원
핵자기공명연구실

Current Data Parameters
NAME dec06-kku-jin
EXPNO 2
PROCNO 1

F2 - Acquisition Parameters
Date_ 20131206
Time 13.16
INSTRUM spect
PROBHD 5 mm Multinucl
PULPROG hmbcgplpndqf
TD 4096
SOLVENT CDCl3
NS 64
DS 16
SWH 5498.534 Hz
FIDRES 1.342415 Hz
AQ 0.3724629 sec
RG 2050
DW 90.933 usec
DE 6.50 usec
TE 298.0 K
CNST2 145.0000000
CNST13 10.0000000
D0 0.00000300 sec
D1 1.50000000 sec
D2 0.00344828 sec
D6 0.05000000 sec
D16 0.00020000 sec
IN0 0.00001730 sec

===== CHANNEL f1 =====
SFO1 500.1325007 MHz
NUC1 1H
P1 10.30 usec
P2 20.60 usec
PLW1 7.00000000 W

===== CHANNEL f2 =====
SFO2 125.7709936 MHz
NUC2 13C
P3 12.00 usec
PLW2 180.00000000 W

===== GRADIENT CHANNEL =====
GPNAM[1] SMSQ10.100
GPNAM[2] SMSQ10.100
GPNAM[3] SMSQ10.100
GPZ1 50.00 %
GPZ2 30.00 %
GPZ3 40.10 %
P16 1000.00 usec

F1 - Acquisition parameters
TD 256
SFO1 125.771 MHz
FIDRES 112.897400 Hz
SW 229.796 ppm
FnMODE QF

F2 - Processing parameters
SI 2048
SF 500.1300080 MHz
WDW SINE
SSB 0
LB 0 Hz
GB 0
PC 1.40

F1 - Processing parameters
SI 1024
MC2 QF
SF 125.7577748 MHz
WDW SINE
SSB 0
LB 0 Hz
GB 0

DP-H-2 / HMBC

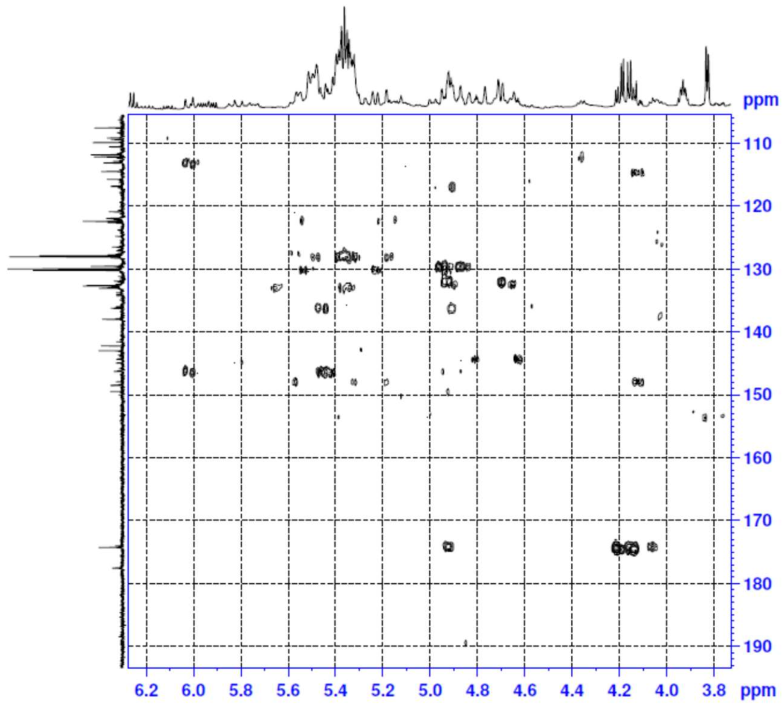

서 울 대 학 교
SEOUL NATIONAL UNIVERSITY
기초과학공동기기원
핵자기공명연구실

Current Data Parameters
NAME dec06-kku-jin
EXPNO 2
PROCNO 1

F2 - Acquisition Parameters
Date_ 20131206
Time 13.16
INSTRUM spect
PROBHD 5 mm Multinucl
PULPROG hmbcgplpndqf
TD 4096
SOLVENT CDCl3
NS 64
DS 16
SWH 5498.534 Hz
FIDRES 1.342415 Hz
AQ 0.3724629 sec
RG 2050
DW 90.933 usec
DE 6.50 usec
TE 298.0 K
CNST2 145.0000000
CNST13 10.0000000
D0 0.00000300 sec
D1 1.50000000 sec
D2 0.00344828 sec
D6 0.05000000 sec
D16 0.00020000 sec
IN0 0.00001730 sec

===== CHANNEL f1 =====
SFO1 500.1325007 MHz
NUC1 1H
P1 10.30 usec
P2 20.60 usec
PLW1 7.00000000 W

===== CHANNEL f2 =====
SFO2 125.7709936 MHz
NUC2 13C
P3 12.00 usec
PLW2 180.00000000 W

===== GRADIENT CHANNEL =====
GPNAM[1] SMSQ10.100
GPNAM[2] SMSQ10.100
GPNAM[3] SMSQ10.100
GPZ1 50.00 %
GPZ2 30.00 %
GPZ3 40.10 %
P16 1000.00 usec

F1 - Acquisition parameters
TD 256
SFO1 125.771 MHz
FIDRES 112.897400 Hz
SW 229.796 ppm
FnMODE QF

F2 - Processing parameters
SI 2048
SF 500.1300080 MHz
WDW SINE
SSB 0
LB 0 Hz
GB 0
PC 1.40

F1 - Processing parameters
SI 1024
MC2 QF
SF 125.7577748 MHz
WDW SINE
SSB 0
LB 0 Hz
GB 0

DP-H-2 / HMBC

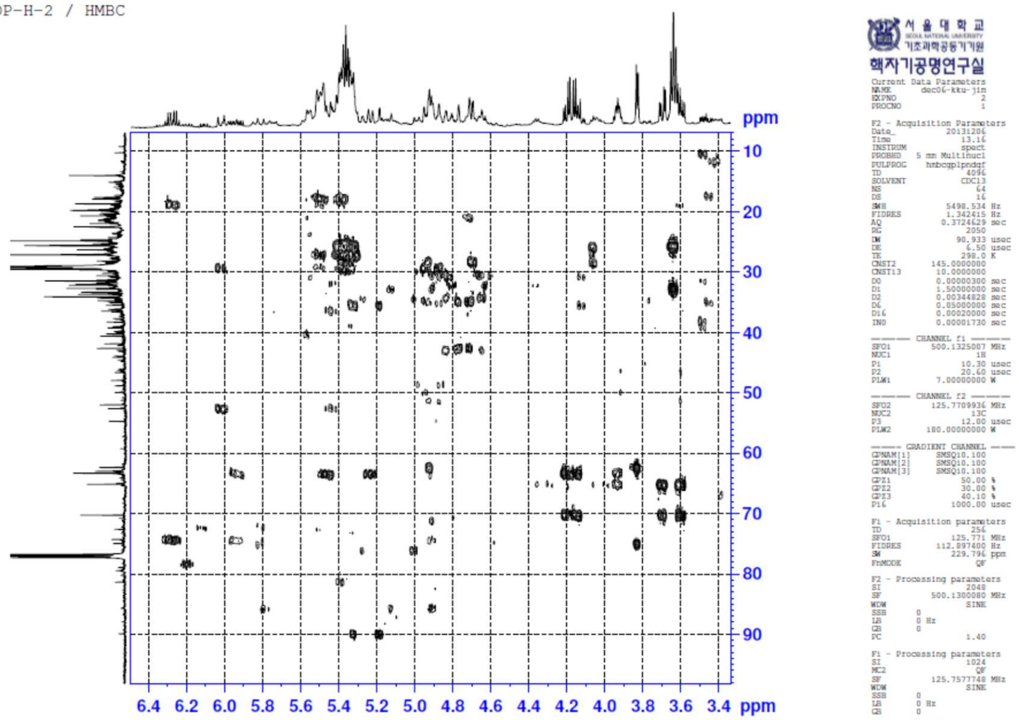

DP-H-2 / HMBC

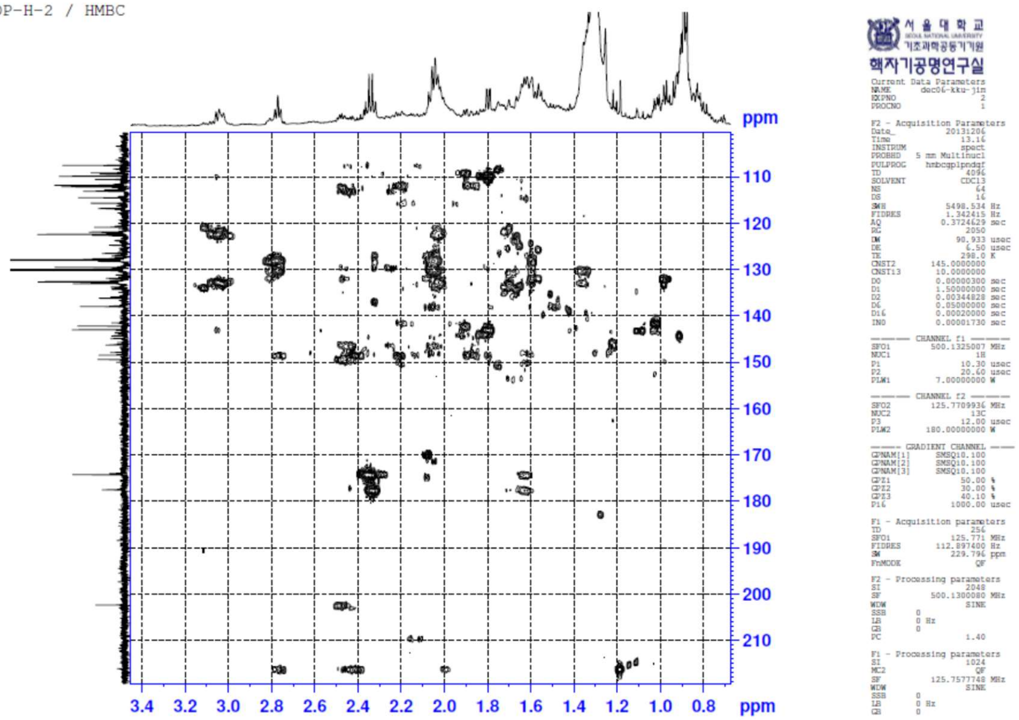

DP-H-2 / HMBC

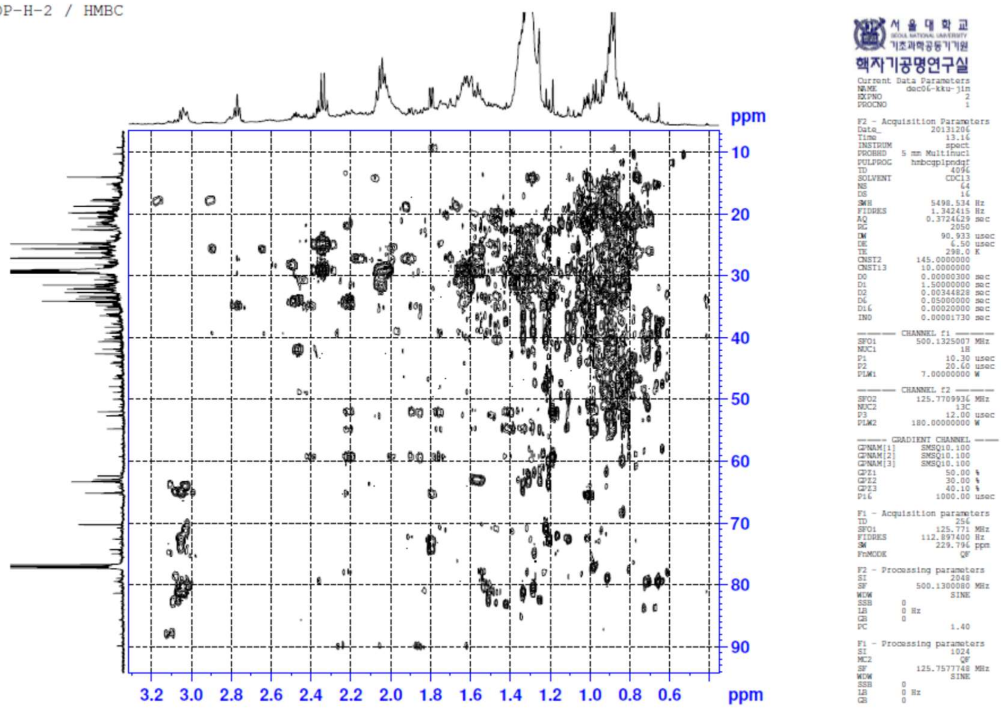

DP-H-2 / HSQC

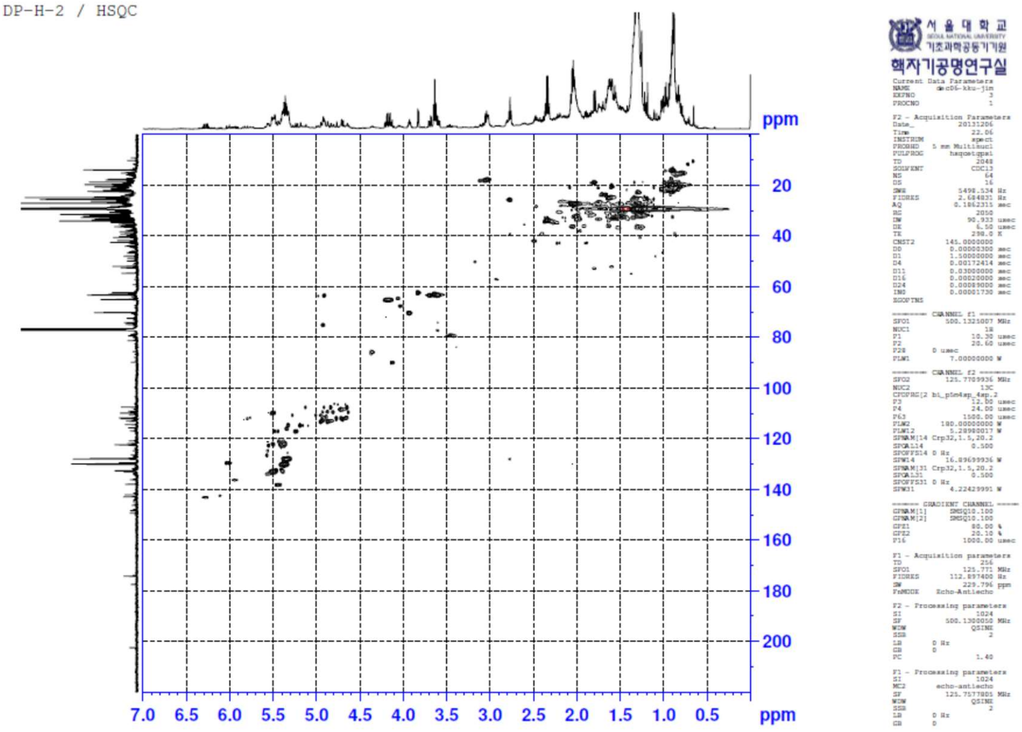

DP-H-2 / HSQC

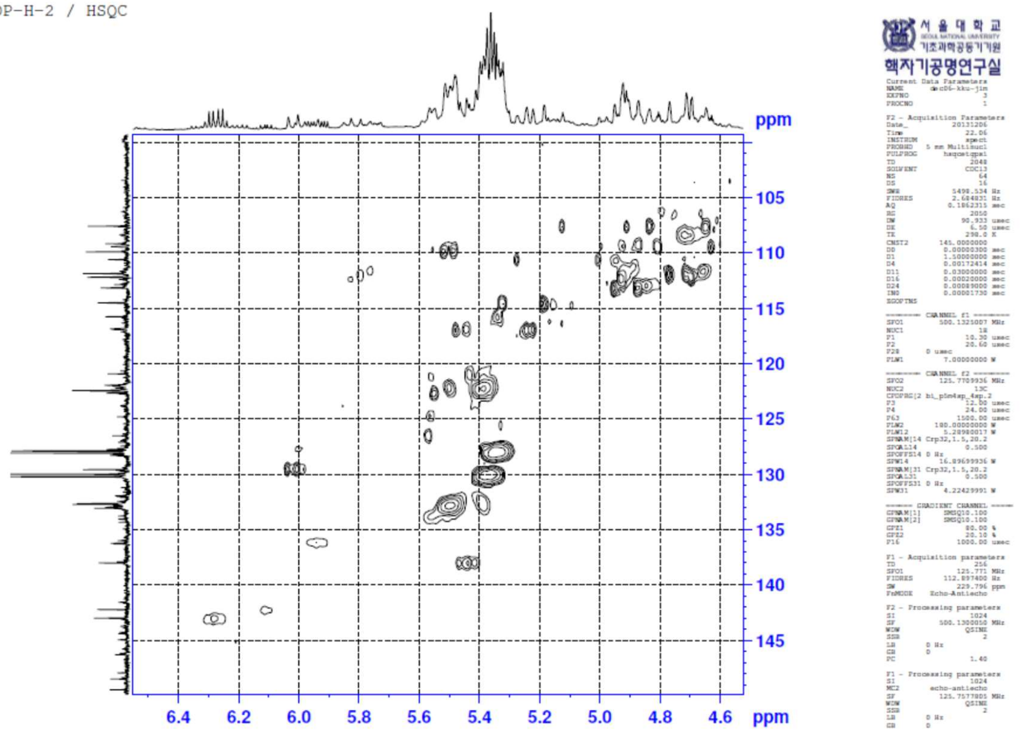

DP-H-2 / HSQC

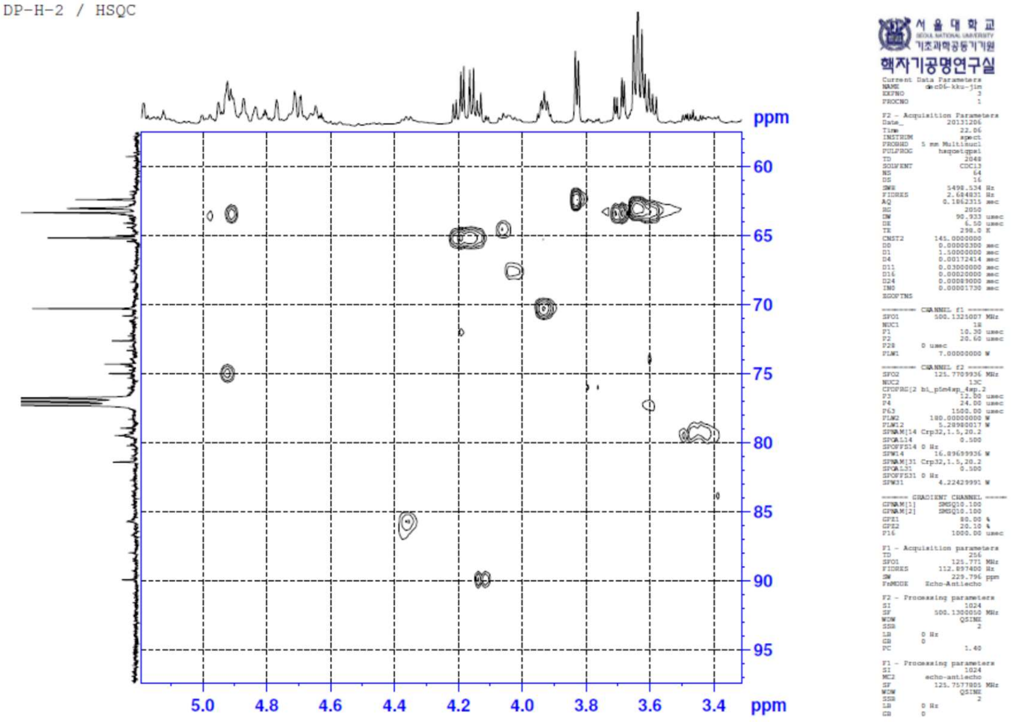

DP-H-2 / HSQC

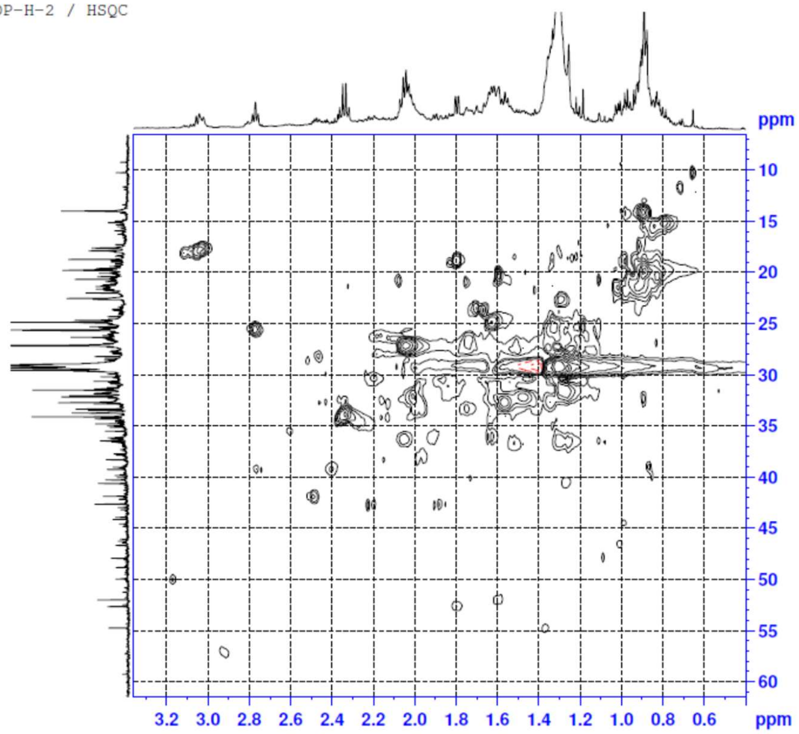

서 울 대 학 교
SEOUL NATIONAL UNIVERSITY
기초과학공동기기원
핵자기공명연구실

```
Current Data Parameters
NAME        dec06-kku-jin
EXPNO                   3
PROCNO                  1

F2 - Acquisition Parameters
Date_            20131206
Time                22.06
INSTRUM             spect
PROBHD   5 mm Multinucl
PULPROG        hsqcetgpsi
TD                   2048
SOLVENT             CDCl3
NS                     64
DS                     16
SWH              5498.534 Hz
FIDRES           2.684831 Hz
AQ              0.1862315 sec
RG                   2050
DW                 90.933 usec
DE                   6.50 usec
TE                  298.0 K
CNST2         145.0000000
D0             0.00000300 sec
D1             1.50000000 sec
D4             0.00172414 sec
D11            0.03000000 sec
D16            0.00020000 sec
D24            0.00089000 sec
IN0            0.00001730 sec
ZGOPTNS

======== CHANNEL f1 ========
SFO1          500.1325007 MHz
NUC1                   1H
P1                  10.30 usec
P2                  20.60 usec
P28      0 usec
PLW1           7.00000000 W

======== CHANNEL f2 ========
SFO2          125.7709936 MHz
NUC2                  13C
CPDPRG[2 bi_p5m4sp_4sp.2
P3                  12.00 usec
P4                  24.00 usec
P63               1500.00 usec
PLW2         180.00000000 W
PLW12          5.28980017 W
SPNAM[14 Crp32,1.5,20.2
SPOAL14             0.500
SPOFFS14 0 Hz
SPW14         16.89699936 W
SPNAM[31 Crp32,1.5,20.2
SPOAL31             0.500
SPOFFS31 0 Hz
SPW31          4.22429991 W

====== GRADIENT CHANNEL =====
GPNAM[1]       SMSQ10.100
GPNAM[2]       SMSQ10.100
GPZ1                80.00 %
GPZ2                20.10 %
P16               1000.00 usec

F1 - Acquisition parameters
TD                    256
SFO1              125.771 MHz
FIDRES         112.897400 Hz
SW                229.796 ppm
FnMODE    Echo-Antiecho

F2 - Processing parameters
SI                   1024
SF            500.1300050 MHz
WDW                 QSINE
SSB                     2
LB       0 Hz
GB       0
PC                   1.40

F1 - Processing parameters
SI                   1024
MC2         echo-antiecho
SF            125.7577805 MHz
WDW                 QSINE
SSB                     2
LB       0 Hz
GB       0
```

DP-H-2 / COSY

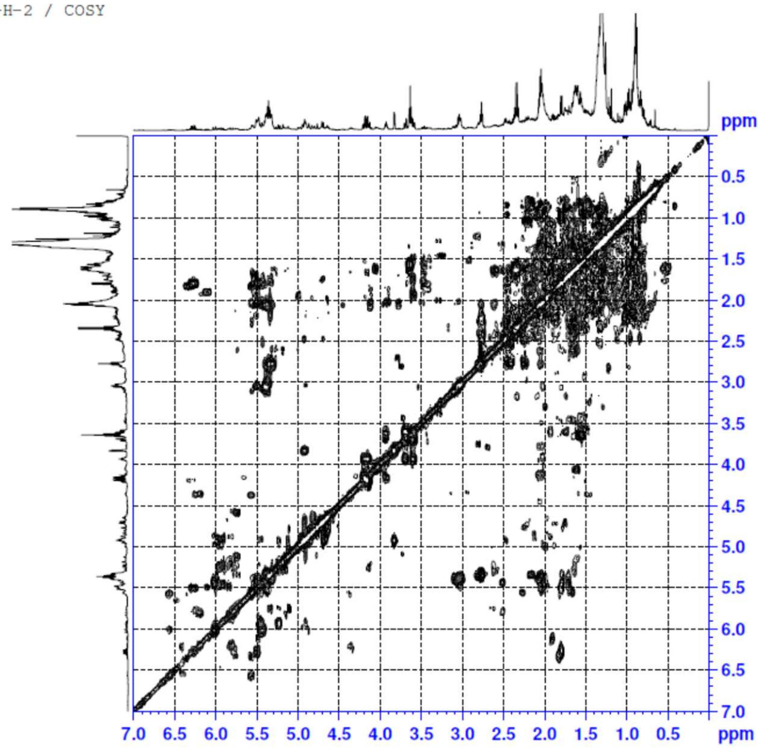

서 울 대 학 교
SEOUL NATIONAL UNIVERSITY
기초과학공동기기원
핵자기공명연구실

```
Current Data Parameters
NAME        dec06-kku-jin
EXPNO                   4
PROCNO                  1

F2 - Acquisition Parameters
Date_            20131207
Time                 5.52
INSTRUM             spect
PROBHD   5 mm Multinucl
PULPROG         cosygpppqf
TD                   2048
SOLVENT             CDCl3
NS                     64
DS                      8
SWH              5498.534 Hz
FIDRES           2.684831 Hz
AQ              0.1862315 sec
RG                   25.4
DW                 90.933 usec
DE                   6.50 usec
TE                  298.0 K
D0             0.00000300 sec
D1             2.00000000 sec
D11            0.03000000 sec
D12            0.00002000 sec
D13            0.00000400 sec
D16            0.00020000 sec
IN0            0.00018180 sec

======== CHANNEL f1 ========
SFO1          500.1325007 MHz
NUC1                   1H
P0                  10.30 usec
P1                  10.30 usec
P17               2500.00 usec
PLW1           7.00000000 W
PLW10          1.05630004 W

====== GRADIENT CHANNEL =====
GPNAM[1]       SMSQ10.100
GPZ1                10.00 %
P16               1000.00 usec

F1 - Acquisition parameters
TD                    256
SFO1             500.1325 MHz
FIDRES          21.486525 Hz
SW                 10.998 ppm
FnMODE                 QF

F2 - Processing parameters
SI                   1024
SF            500.1300085 MHz
WDW                 QSINE
SSB      0
LB       0 Hz
GB       0
PC                   1.40

F1 - Processing parameters
SI                   1024
MC2                    QF
SF            500.1300079 MHz
WDW                 QSINE
SSB      0
LB       0 Hz
GB       0
```

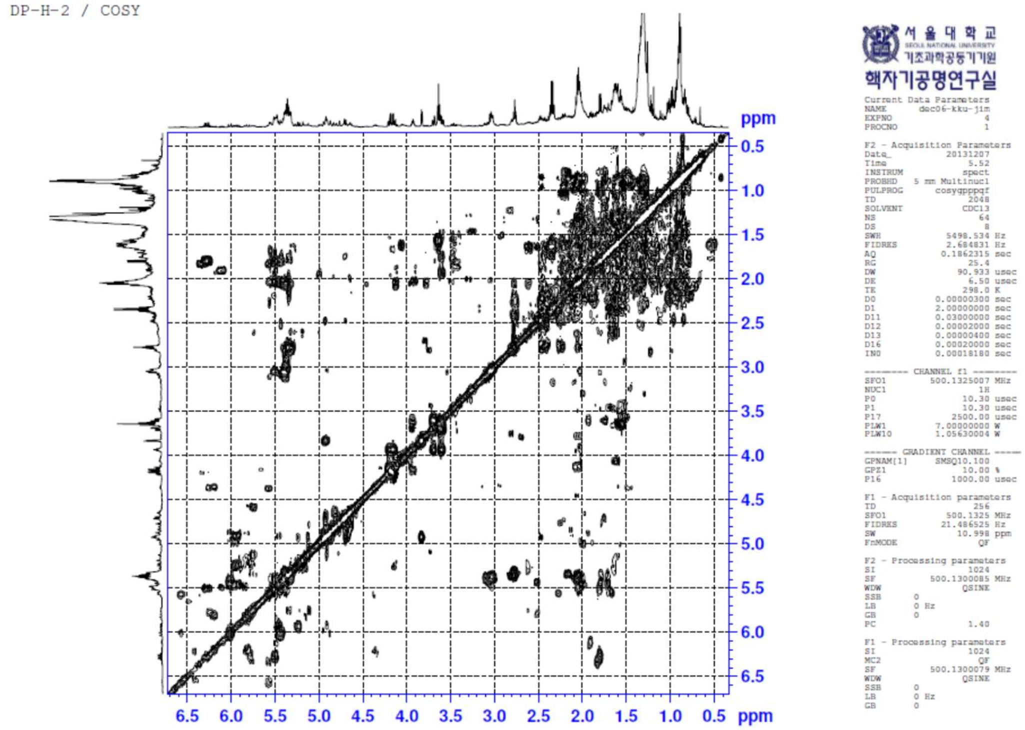
DP-H-2 / COSY
ppm
6.5 6.0 5.5 5.0 4.5 4.0 3.5 3.0 2.5 2.0 1.5 1.0 0.5 ppm
0.5 1.0 1.5 2.0 2.5 3.0 3.5 4.0 4.5 5.0 5.5 6.0 6.5
서 울 대 학 교
SEOUL NATIONAL UNIVERSITY
기초과학공동기기원
핵자기공명연구실
Current Data Parameters
NAME dec06-kku-jim
EXPNO 4
PROCNO 1
F2 - Acquisition Parameters
Date_ 20131207
Time 5.52
INSTRUM spect
PROBHD 5 mm Multinucl
PULPROG cosygpppqf
TD 2048
SOLVENT CDCl3
NS 64
DS 8
SWH 5498.534 Hz
FIDRES 2.684831 Hz
AQ 0.1862315 sec
RG 25.4
DW 90.933 usec
DE 6.50 usec
TE 298.0 K
D0 0.00000300 sec
D1 2.00000000 sec
D11 0.03000000 sec
D12 0.00002000 sec
D13 0.00000400 sec
D16 0.00020000 sec
IN0 0.00018180 sec
======== CHANNEL f1 ========
SFO1 500.1325007 MHz
NUC1 1H
P0 10.30 usec
P1 10.30 usec
P17 2500.00 usec
PLW1 7.00000000 W
PLW10 1.05630004 W
====== GRADIENT CHANNEL =====
GPNAM[1] SMSQ10.100
GPZ1 10.00 %
P16 1000.00 usec
F1 - Acquisition parameters
TD 256
SFO1 500.1325 MHz
FIDRES 21.486525 Hz
SW 10.998 ppm
FnMODE QF
F2 - Processing parameters
SI 1024
SF 500.1300085 MHz
WDW QSINE
SSB 0
LB 0 Hz
GB 0
PC 1.40
F1 - Processing parameters
SI 1024
MC2 QF
SF 500.1300079 MHz
WDW QSINE
SSB 0
LB 0 Hz
GB 0

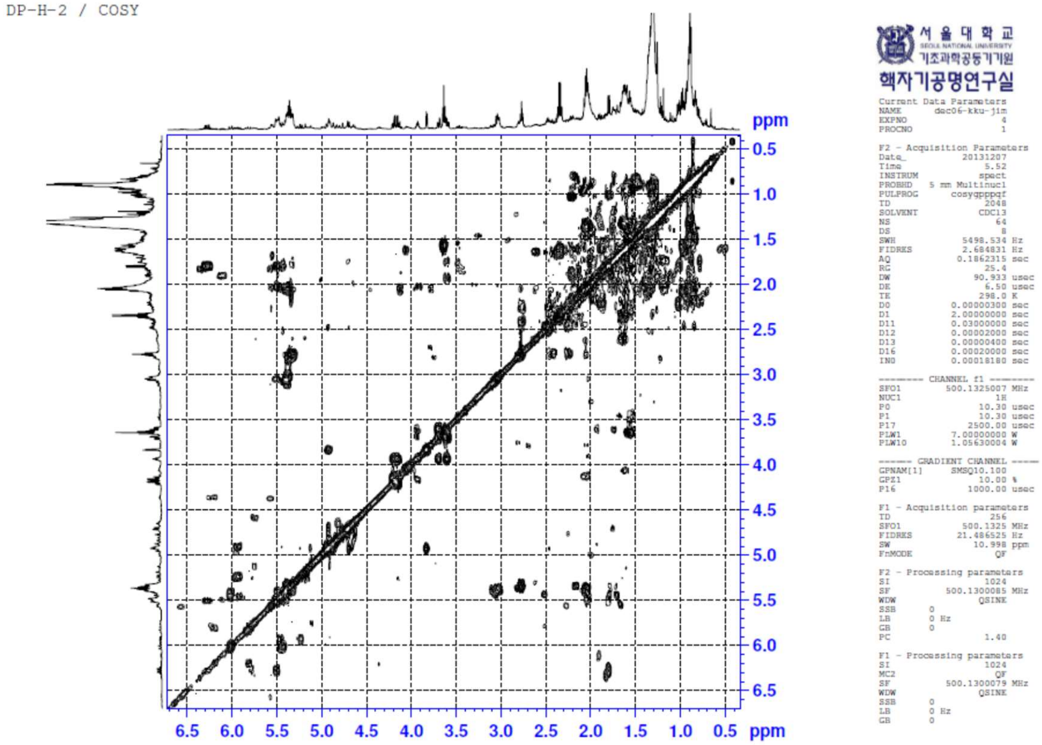
DP-H-2 / COSY
ppm
6.5 6.0 5.5 5.0 4.5 4.0 3.5 3.0 2.5 2.0 1.5 1.0 0.5 ppm
0.5 1.0 1.5 2.0 2.5 3.0 3.5 4.0 4.5 5.0 5.5 6.0 6.5
서 울 대 학 교
SEOUL NATIONAL UNIVERSITY
기초과학공동기기원
핵자기공명연구실
Current Data Parameters
NAME dec06-kku-jim
EXPNO 4
PROCNO 1
F2 - Acquisition Parameters
Date_ 20131207
Time 5.52
INSTRUM spect
PROBHD 5 mm Multinucl
PULPROG cosygpppqf
TD 2048
SOLVENT CDCl3
NS 64
DS 8
SWH 5498.534 Hz
FIDRES 2.684831 Hz
AQ 0.1862315 sec
RG 25.4
DW 90.933 usec
DE 6.50 usec
TE 298.0 K
D0 0.00000300 sec
D1 2.00000000 sec
D11 0.03000000 sec
D12 0.00002000 sec
D13 0.00000400 sec
D16 0.00020000 sec
IN0 0.00018180 sec
======== CHANNEL f1 ========
SFO1 500.1325007 MHz
NUC1 1H
P0 10.30 usec
P1 10.30 usec
P17 2500.00 usec
PLW1 7.00000000 W
PLW10 1.05630004 W
====== GRADIENT CHANNEL =====
GPNAM[1] SMSQ10.100
GPZ1 10.00 %
P16 1000.00 usec
F1 - Acquisition parameters
TD 256
SFO1 500.1325 MHz
FIDRES 21.486525 Hz
SW 10.998 ppm
FnMODE QF
F2 - Processing parameters
SI 1024
SF 500.1300085 MHz
WDW QSINE
SSB 0
LB 0 Hz
GB 0
PC 1.40
F1 - Processing parameters
SI 1024
MC2 QF
SF 500.1300079 MHz
WDW QSINE
SSB 0
LB 0 Hz
GB 0

FAB MS

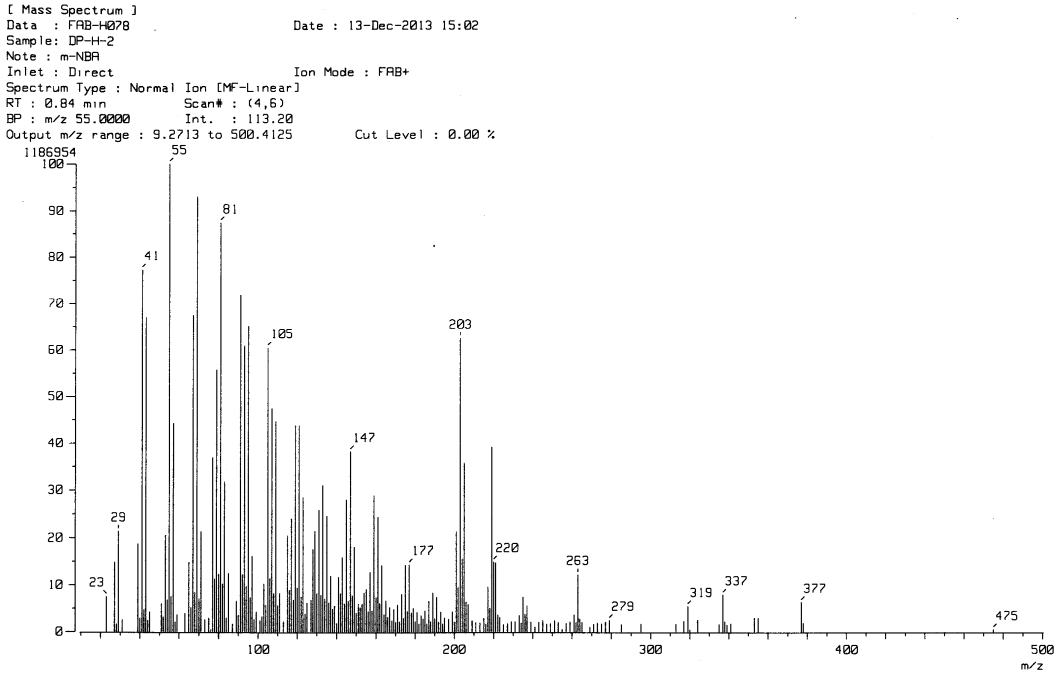
[Mass Spectrum]
Data : FAB-H078
Date : 13-Dec-2013 15:02
Sample: DP-H-2
Note : m-NBA
Inlet : Direct
Ion Mode : FAB+
Spectrum Type : Normal Ion [MF-Linear]
RT : 0.84 min
Scan# : (4,6)
BP : m/z 55.0000
Int. : 113.20
Output m/z range : 9.2713 to 500.4125
Cut Level : 0.00 %
1186954
23
29
41
55
81
105
147
177
203
220
263
279
319
337
377
475
m/z

HRFTMS

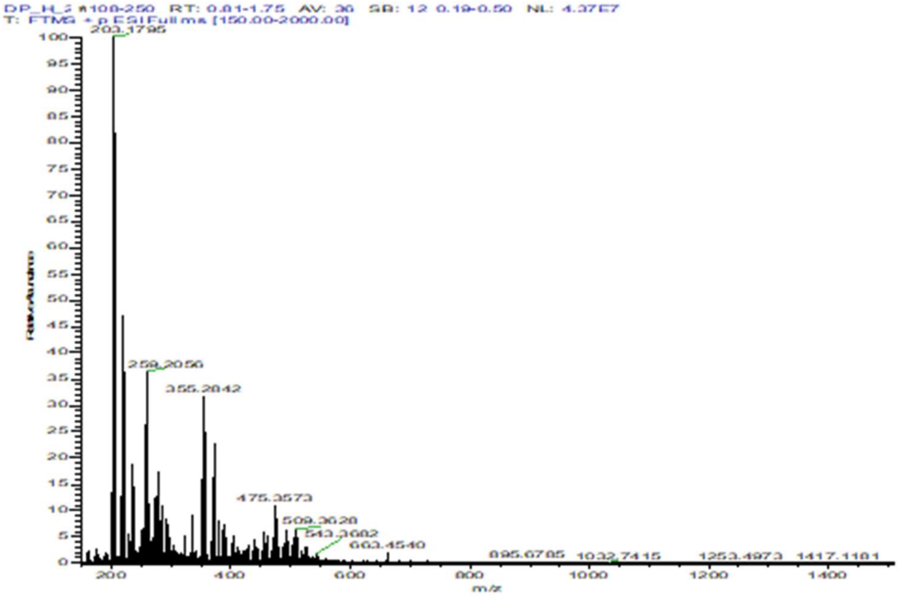
DP_H_2 #108-250 RT: 0.81-1.75 AV: 36 SB: 12 0.19-0.50 NL: 4.37E7
T: FTMS + p ESI Full ms [150.00-2000.00]
203.1795
259.2056
355.2842
475.3573
509.3628
543.3682
663.4540
895.6785
1032.7415
1253.4973
1417.1181
m/z

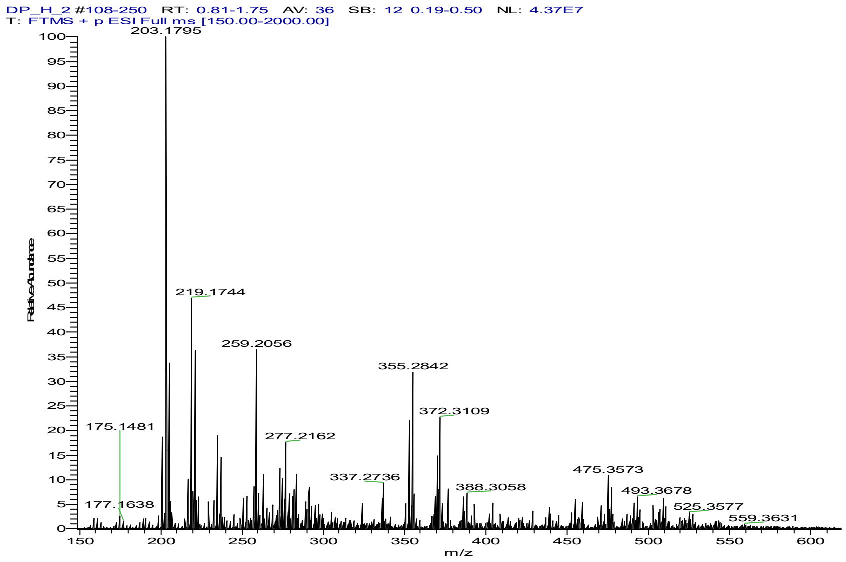
DP_H_2 #108-250 RT: 0.81-1.75 AV: 36 SB: 12 0.19-0.50 NL: 4.37E7
T: FTMS + p ESI Full ms [150.00-2000.00]
203.1795
219.1744
259.2056
355.2842
372.3109
175.1481
277.2162
337.2736
475.3573
388.3058
493.3678
177.1638
525.3577
559.3631
Relative Abundance
m/z

Compound 2

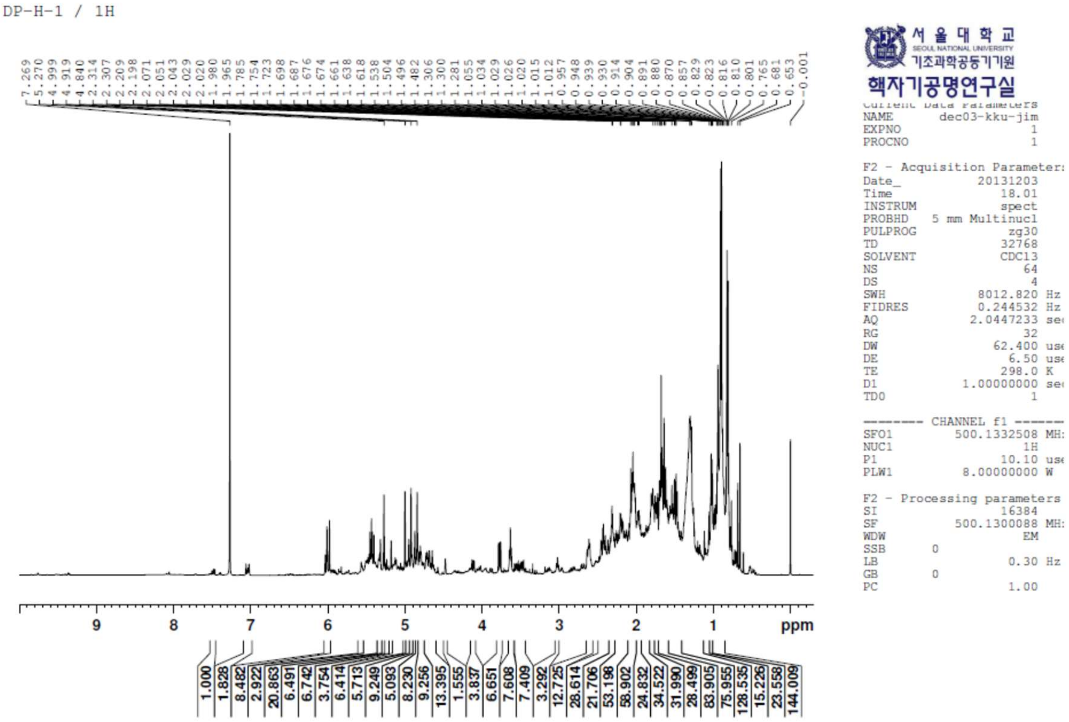
DP-H-1 / 1H
서울대학교
SEOUL NATIONAL UNIVERSITY
기초과학공동기기원
핵자기공명연구실
NAME dec03-kku-jim
EXPNO 1
PROCNO 1
F2 - Acquisition Parameters
Date_ 20131203
Time 18.01
INSTRUM spect
PROBHD 5 mm Multinucl
PULPROG zg30
TD 32768
SOLVENT CDCl3
NS 64
DS 4
SWH 8012.820 Hz
FIDRES 0.244532 Hz
AQ 2.0447233 sec
RG 32
DW 62.400 usec
DE 6.50 usec
TE 298.0 K
D1 1.00000000 sec
TD0 1
CHANNEL f1
SFO1 500.1332508 MHz
NUC1 1H
P1 10.10 usec
PLW1 8.00000000 W
F2 - Processing parameters
SI 16384
SF 500.1300088 MHz
WDW EM
SSB 0
LB 0.30 Hz
GB 0
PC 1.00
ppm

DP-H-1 / 1H

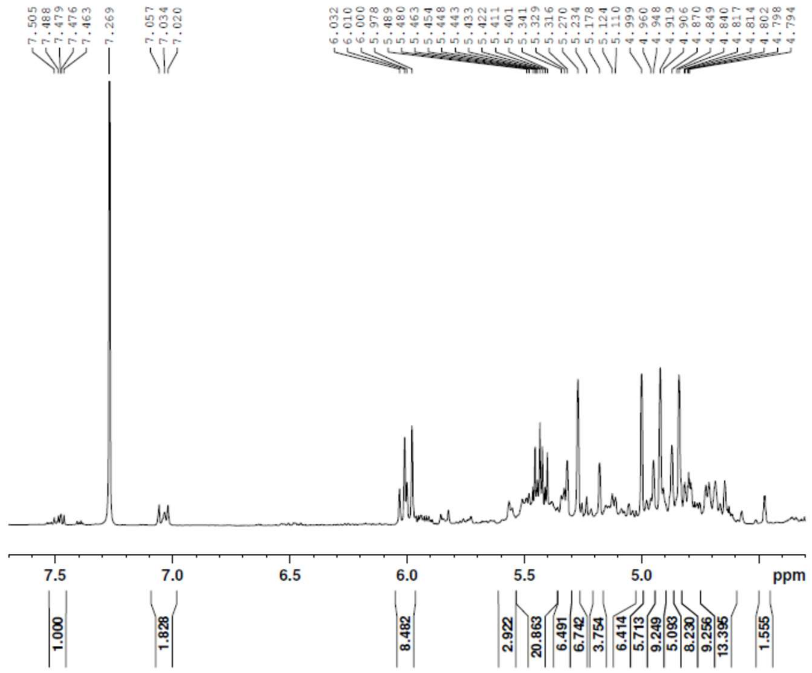

서 울 대 학 교
SEOUL NATIONAL UNIVERSITY
기초과학공동기기원
핵자기공명연구실

```
Current Data Parameters
NAME        dec03-kku-jim
EXPNO                   1
PROCNO                  1

F2 - Acquisition Parameters
Date_            20131203
Time                18.01
INSTRUM             spect
PROBHD   5 mm Multinucl
PULPROG              zg30
TD                  32768
SOLVENT             CDCl3
NS                     64
DS                      4
SWH             8012.820 Hz
FIDRES          0.244532 Hz
AQ             2.0447233 sec
RG                     32
DW                 62.400 usec
DE                   6.50 usec
TE                  298.0 K
D1            1.00000000 sec
TD0                     1

======== CHANNEL f1 ========
SFO1         500.1332508 MHz
NUC1                   1H
P1                  10.10 usec
PLW1           8.00000000 W

F2 - Processing parameters
SI                  16384
SF           500.1300088 MHz
WDW                    EM
SSB      0
LB                   0.30 Hz
GB       0
PC                   1.00
```

DP-H-1 / 1H

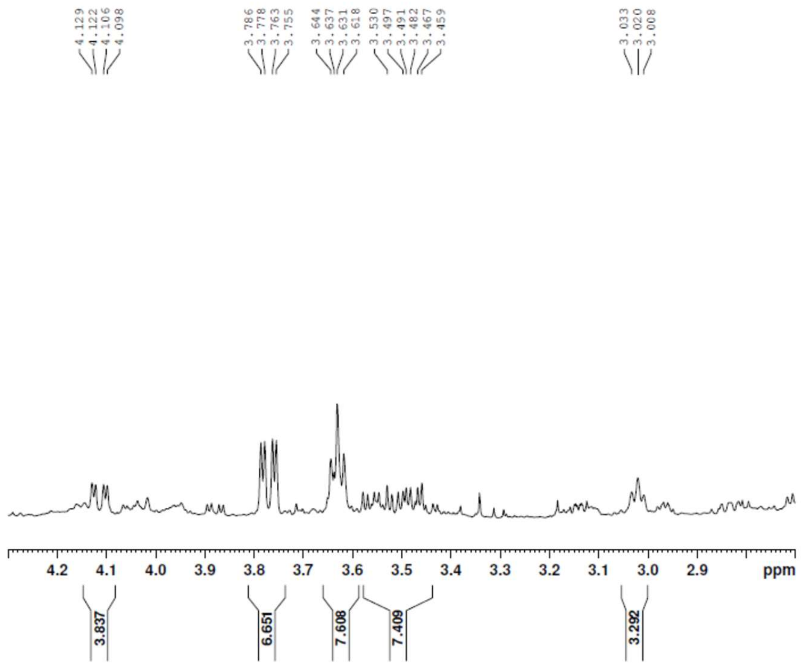

서 울 대 학 교
SEOUL NATIONAL UNIVERSITY
기초과학공동기기원
핵자기공명연구실


```
Current Data Parameters
NAME        dec03-kku-jim
EXPNO                   1
PROCNO                  1

F2 - Acquisition Parameters
Date_            20131203
Time                18.01
INSTRUM             spect
PROBHD   5 mm Multinucl
PULPROG              zg30
TD                  32768
SOLVENT             CDCl3
NS                     64
DS                      4
SWH             8012.820 Hz
FIDRES          0.244532 Hz
AQ             2.0447233 sec
RG                     32
DW                 62.400 usec
DE                   6.50 usec
TE                  298.0 K
D1            1.00000000 sec
TD0                     1

======== CHANNEL f1 ========
SFO1         500.1332508 MHz
NUC1                   1H
P1                  10.10 usec
PLW1           8.00000000 W

F2 - Processing parameters
SI                  16384
SF           500.1300088 MHz
WDW                    EM
SSB      0
LB                   0.30 Hz
GB       0
PC                   1.00
```

DP-H-1 / 1H

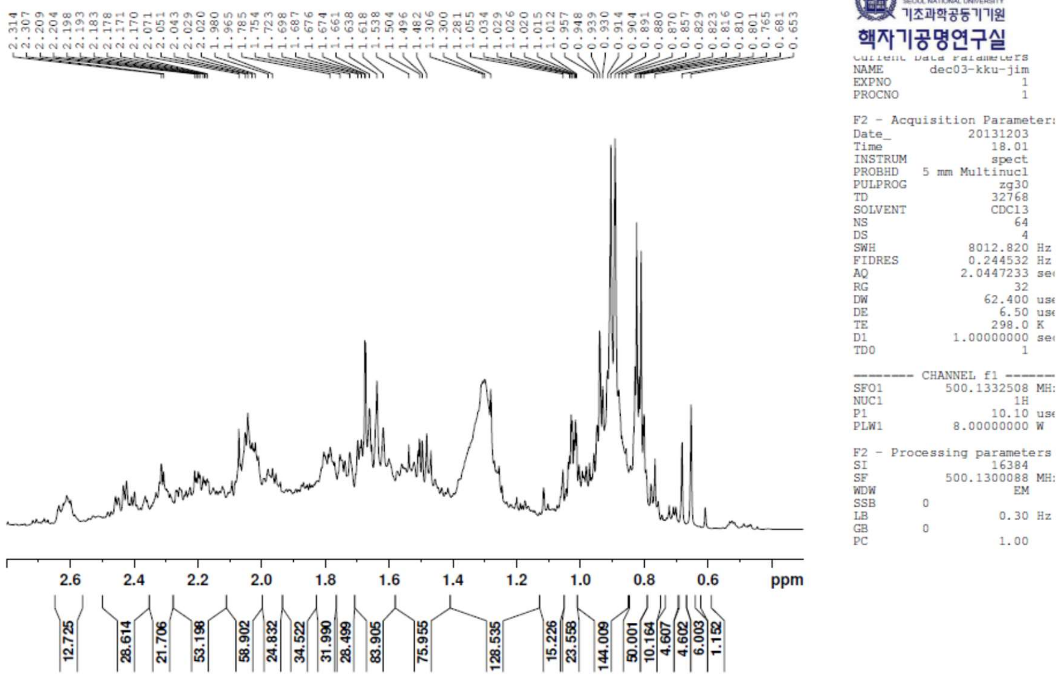

DP-H-1 / 13C

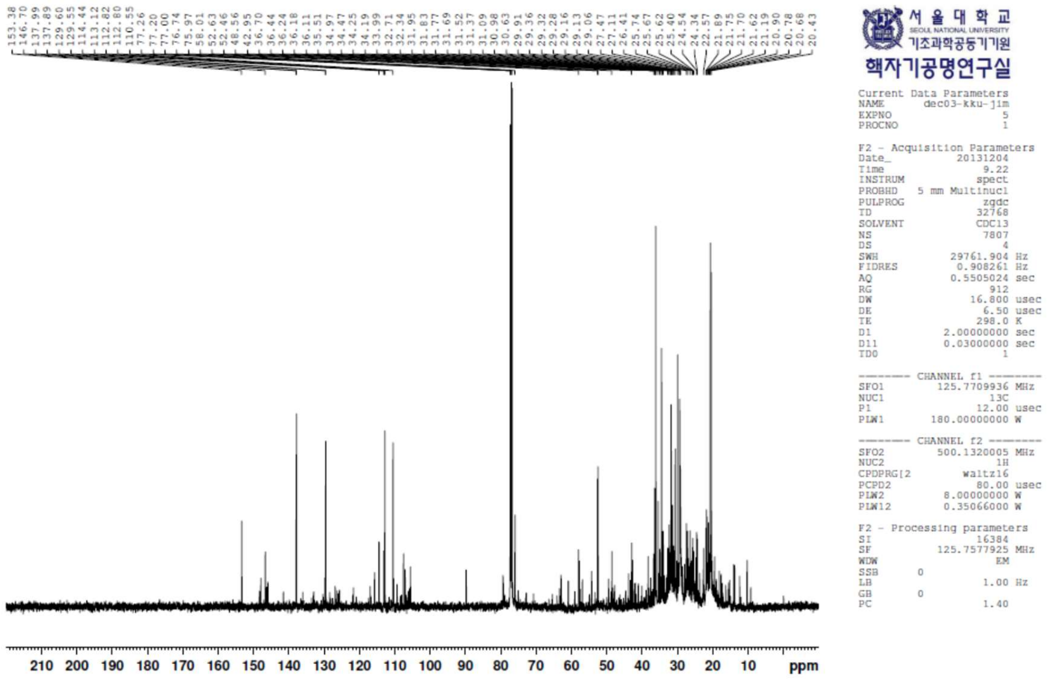

DP-H-1 / 13C

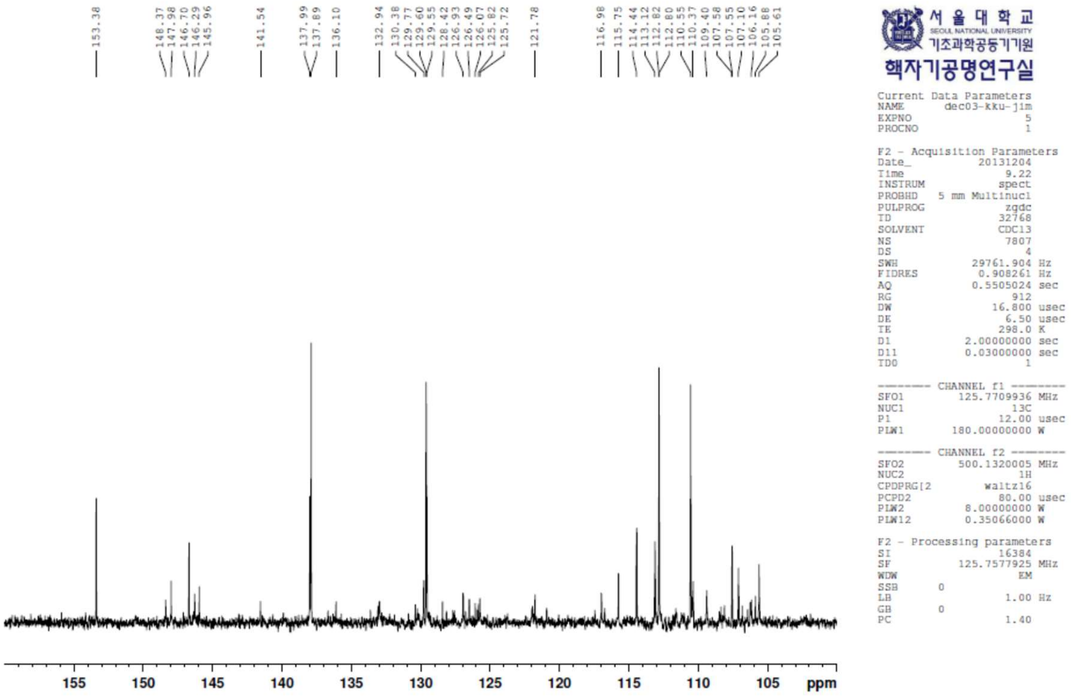

DP-H-1 / 13C

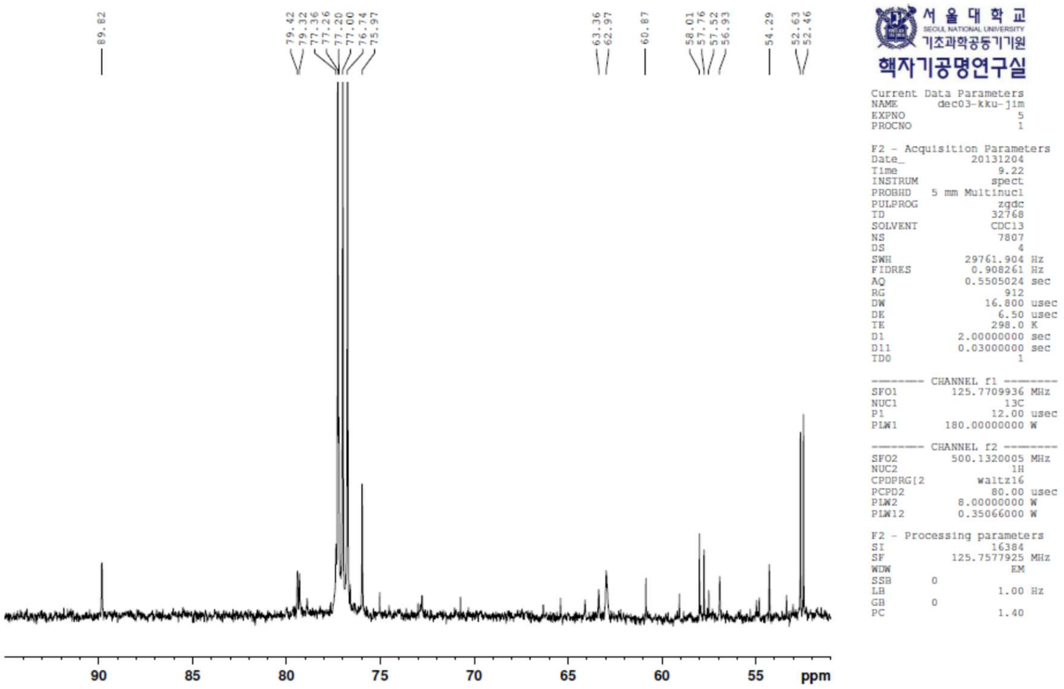

DP-H-1 / 13C

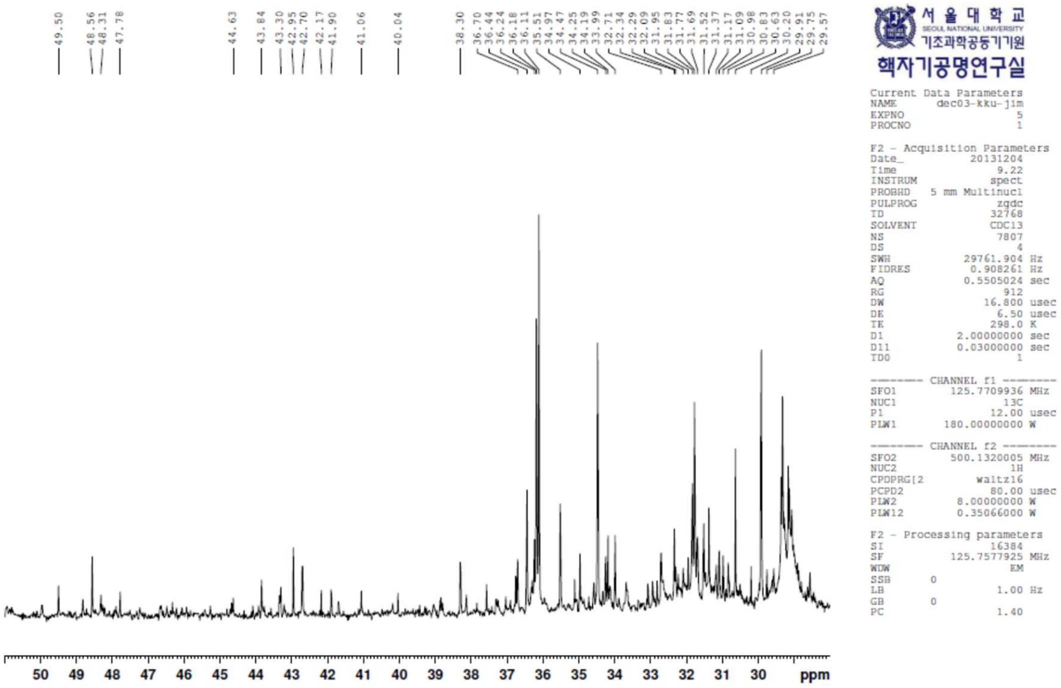

DP-H-1 / 13C

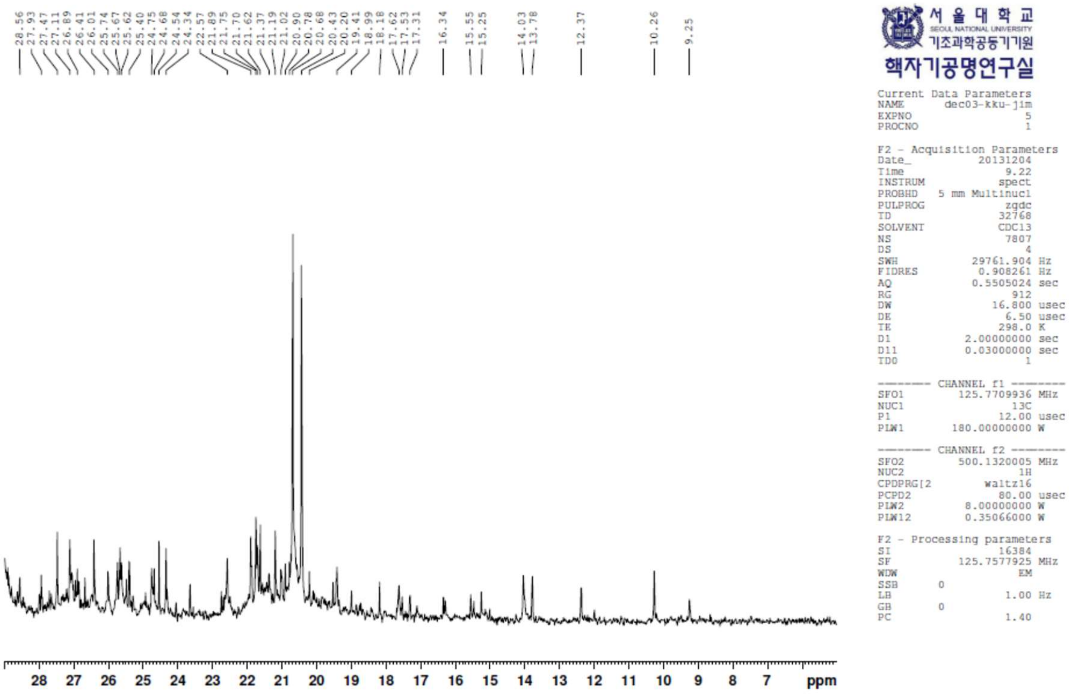

DP-H-1 / HMBC

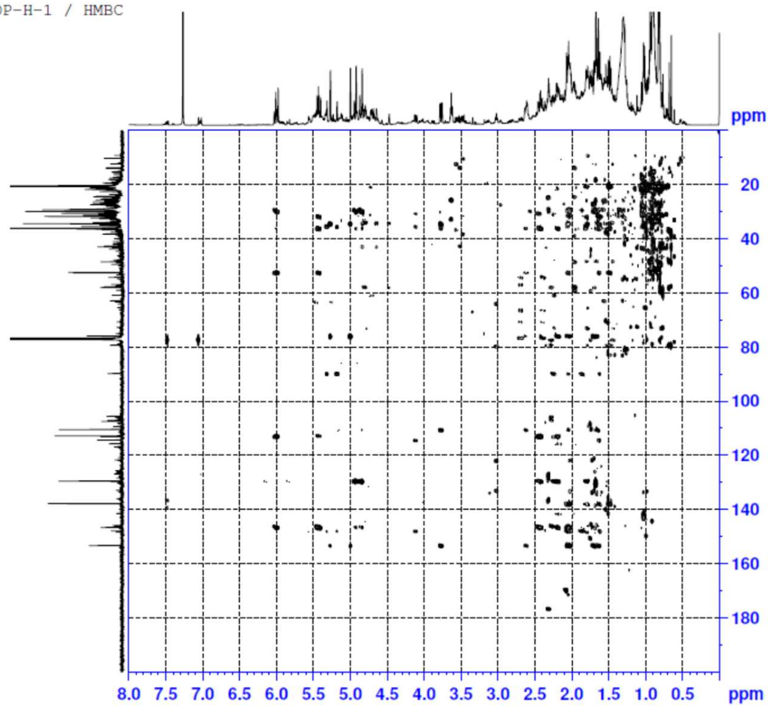

서 울 대 학 교
SEOUL NATIONAL UNIVERSITY
기초과학공동기기원
핵자기공명연구실

```
Current Data Parameters
NAME          dec03-kku-jin
EXPNO                     2
PROCNO                    1

F2 - Acquisition Parameters
Date_              20131203
Time                  18.03
INSTRUM               spect
PROBHD   5 mm Multinucl
PULPROG        hmbcgplpndqf
TD                     4096
SOLVENT               CDCl3
NS                       32
DS                       16
SWH                5411.255 Hz
FIDRES             1.321107 Hz
AQ                0.3784704 sec
RG                     2050
DW                   92.400 usec
DE                     6.50 usec
TE                    298.0 K
CNST2           145.0000000
CNST13           10.0000000
D0               0.00000300 sec
D1               1.50000000 sec
D2               0.00344828 sec
D6               0.05000000 sec
D16              0.00020000 sec
IN0              0.00001730 sec

======== CHANNEL f1 ========
SFO1            500.1324006 MHz
NUC1                     1H
P1                    10.40 usec
P2                    20.80 usec
PLW1             7.00000000 W

======== CHANNEL f2 ========
SFO2            125.7709936 MHz
NUC2                    13C
P3                    12.00 usec
PLW2           180.00000000 W

====== GRADIENT CHANNEL =====
GPNAM[1]         SMSQ10.100
GPNAM[2]         SMSQ10.100
GPNAM[3]         SMSQ10.100
GPZ1                  50.00 %
GPZ2                  30.00 %
GPZ3                  40.10 %
P16                 1000.00 usec

F1 - Acquisition parameters
TD                      256
SFO1                125.771 MHz
FIDRES           112.897400 Hz
SW                  229.796 ppm
FnMODE                   QF

F2 - Processing parameters
SI                     2048
SF              500.1300088 MHz
WDW                    SINE
SSB       0
LB        0 Hz
GB        0
PC                     1.40

F1 - Processing parameters
SI                     1024
MC2                      QF
SF              125.7577829 MHz
WDW                    SINE
SSB       0
LB        0 Hz
GB        0
```

DP-H-1 / HMBC

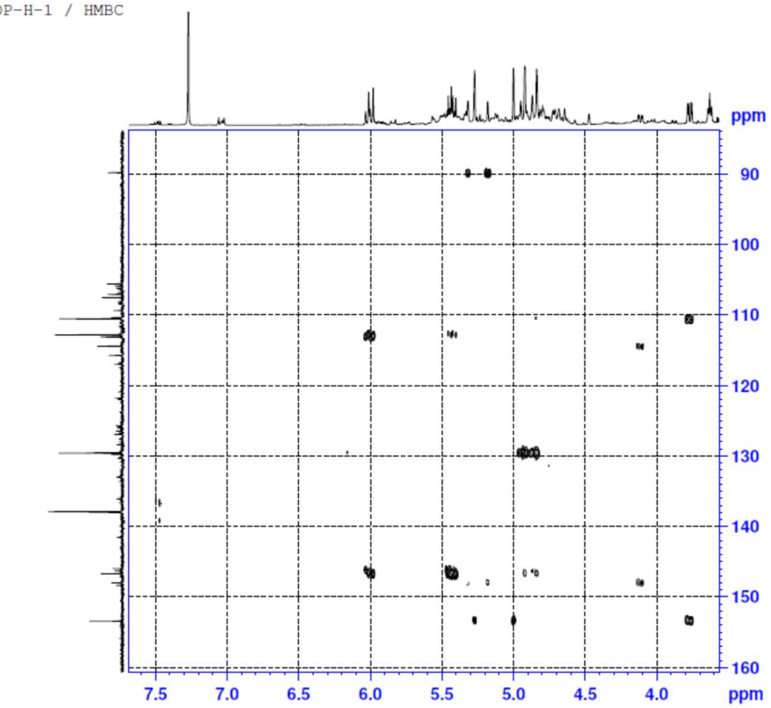

서 울 대 학 교
SEOUL NATIONAL UNIVERSITY
기초과학공동기기원
핵자기공명연구실

```
Current Data Parameters
NAME          dec03-kku-jin
EXPNO                     2
PROCNO                    1

F2 - Acquisition Parameters
Date_              20131203
Time                  18.03
INSTRUM               spect
PROBHD   5 mm Multinucl
PULPROG        hmbcgplpndqf
TD                     4096
SOLVENT               CDCl3
NS                       32
DS                       16
SWH                5411.255 Hz
FIDRES             1.321107 Hz
AQ                0.3784704 sec
RG                     2050
DW                   92.400 usec
DE                     6.50 usec
TE                    298.0 K
CNST2           145.0000000
CNST13           10.0000000
D0               0.00000300 sec
D1               1.50000000 sec
D2               0.00344828 sec
D6               0.05000000 sec
D16              0.00020000 sec
IN0              0.00001730 sec

======== CHANNEL f1 ========
SFO1            500.1324006 MHz
NUC1                     1H
P1                    10.40 usec
P2                    20.80 usec
PLW1             7.00000000 W

======== CHANNEL f2 ========
SFO2            125.7709936 MHz
NUC2                    13C
P3                    12.00 usec
PLW2           180.00000000 W

====== GRADIENT CHANNEL =====
GPNAM[1]         SMSQ10.100
GPNAM[2]         SMSQ10.100
GPNAM[3]         SMSQ10.100
GPZ1                  50.00 %
GPZ2                  30.00 %
GPZ3                  40.10 %
P16                 1000.00 usec

F1 - Acquisition parameters
TD                      256
SFO1                125.771 MHz
FIDRES           112.897400 Hz
SW                  229.796 ppm
FnMODE                   QF

F2 - Processing parameters
SI                     2048
SF              500.1300088 MHz
WDW                    SINE
SSB       0
LB        0 Hz
GB        0
PC                     1.40

F1 - Processing parameters
SI                     1024
MC2                      QF
SF              125.7577829 MHz
WDW                    SINE
SSB       0
LB        0 Hz
GB        0
```

DP-H-1 / HMBC

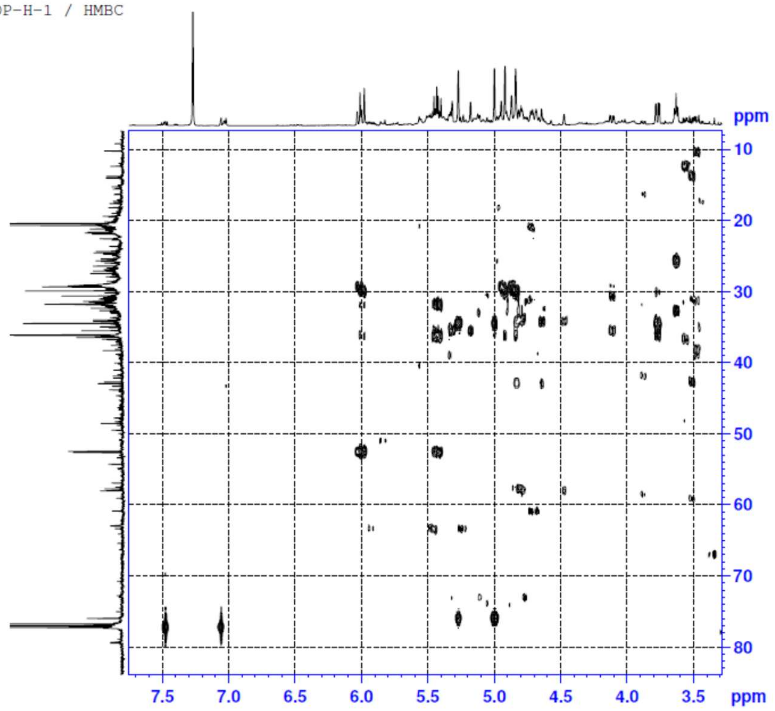

서 울 대 학 교
SEOUL NATIONAL UNIVERSITY
기초과학공동기기원
핵자기공명연구실

Current Data Parameters
NAME dec03-kku-jin
EXPNO 2
PROCNO 1

F2 - Acquisition Parameters
Date_ 20131203
Time 18.03
INSTRUM spect
PROBHD 5 mm Multinucl
PULPROG hmbcgplpndqf
TD 4096
SOLVENT CDCl3
NS 32
DS 16
SWH 5411.255 Hz
FIDRES 1.321107 Hz
AQ 0.3784704 sec
RG 2050
DW 92.400 usec
DE 6.50 usec
TE 298.0 K
CNST2 145.0000000
CNST13 10.0000000
D0 0.00000300 sec
D1 1.50000000 sec
D2 0.00344828 sec
D6 0.05000000 sec
D16 0.00020000 sec
IN0 0.00001730 sec

======== CHANNEL f1 ========
SFO1 500.1324006 MHz
NUC1 1H
P1 10.40 usec
P2 20.80 usec
PLW1 7.00000000 W

======== CHANNEL f2 ========
SFO2 125.7709936 MHz
NUC2 13C
P3 12.00 usec
PLW2 180.00000000 W

====== GRADIENT CHANNEL =====
GPNAM[1] SMSQ10.100
GPNAM[2] SMSQ10.100
GPNAM[3] SMSQ10.100
GPZ1 50.00 %
GPZ2 30.00 %
GPZ3 40.10 %
P16 1000.00 usec

F1 - Acquisition parameters
TD 256
SFO1 125.771 MHz
FIDRES 112.897400 Hz
SW 229.796 ppm
FnMODE QF

F2 - Processing parameters
SI 2048
SF 500.1300088 MHz
WDW SINE
SSB 0
LB 0 Hz
GB 0
PC 1.40

F1 - Processing parameters
SI 1024
MC2 QF
SF 125.7577829 MHz
WDW SINE
SSB 0
LB 0 Hz
GB 0

DP-H-1 / HMBC

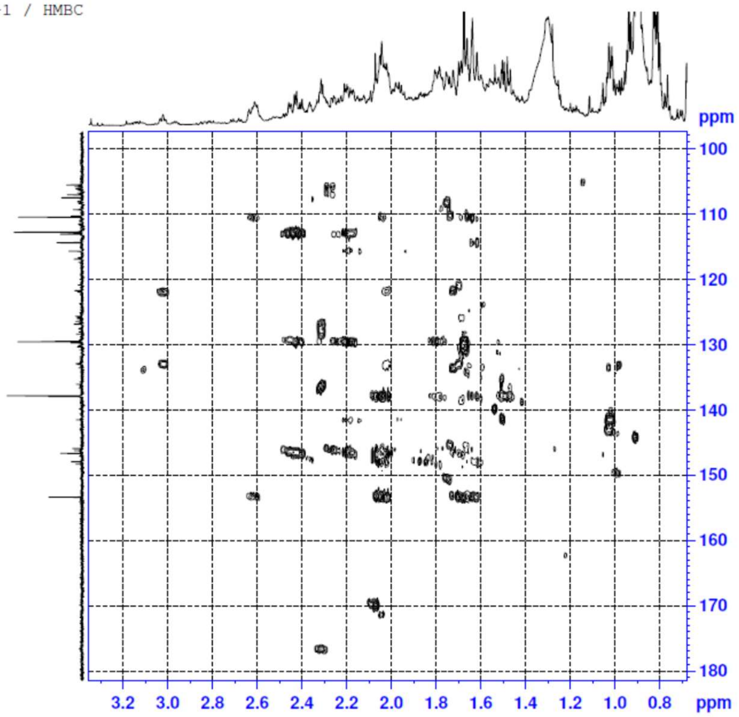

서 울 대 학 교
SEOUL NATIONAL UNIVERSITY
기초과학공동기기원
핵자기공명연구실

Current Data Parameters
NAME dec03-kku-jin
EXPNO 2
PROCNO 1

F2 - Acquisition Parameters
Date_ 20131203
Time 18.03
INSTRUM spect
PROBHD 5 mm Multinucl
PULPROG hmbcgplpndqf
TD 4096
SOLVENT CDCl3
NS 32
DS 16
SWH 5411.255 Hz
FIDRES 1.321107 Hz
AQ 0.3784704 sec
RG 2050
DW 92.400 usec
DE 6.50 usec
TE 298.0 K
CNST2 145.0000000
CNST13 10.0000000
D0 0.00000300 sec
D1 1.50000000 sec
D2 0.00344828 sec
D6 0.05000000 sec
D16 0.00020000 sec
IN0 0.00001730 sec

======== CHANNEL f1 ========
SFO1 500.1324006 MHz
NUC1 1H
P1 10.40 usec
P2 20.80 usec
PLW1 7.00000000 W

======== CHANNEL f2 ========
SFO2 125.7709936 MHz
NUC2 13C
P3 12.00 usec
PLW2 180.00000000 W

====== GRADIENT CHANNEL =====
GPNAM[1] SMSQ10.100
GPNAM[2] SMSQ10.100
GPNAM[3] SMSQ10.100
GPZ1 50.00 %
GPZ2 30.00 %
GPZ3 40.10 %
P16 1000.00 usec

F1 - Acquisition parameters
TD 256
SFO1 125.771 MHz
FIDRES 112.897400 Hz
SW 229.796 ppm
FnMODE QF

F2 - Processing parameters
SI 2048
SF 500.1300088 MHz
WDW SINE
SSB 0
LB 0 Hz
GB 0
PC 1.40

F1 - Processing parameters
SI 1024
MC2 QF
SF 125.7577829 MHz
WDW SINE
SSB 0
LB 0 Hz
GB 0

DP-H-1 / HMBC

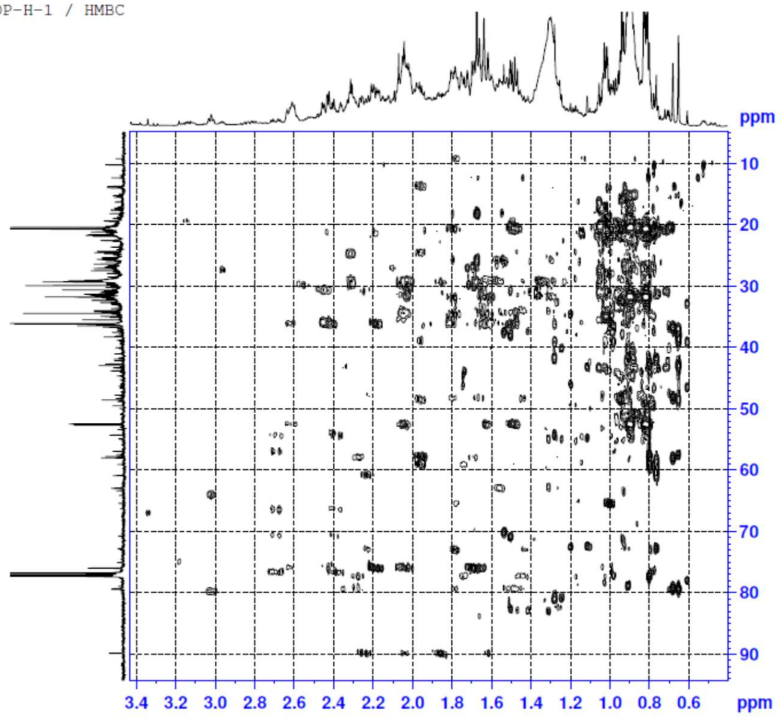

서 울 대 학 교
SEOUL NATIONAL UNIVERSITY
기초과학공동기기원
핵자기공명연구실

```
Current Data Parameters
NAME          dec03-kku-jin
EXPNO                     2
PROCNO                    1

F2 - Acquisition Parameters
Date_              20131203
Time                  18.03
INSTRUM               spect
PROBHD    5 mm Multinucl
PULPROG        hmbcgplpndqf
TD                     4096
SOLVENT               CDCl3
NS                       32
DS                       16
SWH                5411.255 Hz
FIDRES             1.321107 Hz
AQ                0.3784704 sec
RG                     2050
DW                   92.400 usec
DE                     6.50 usec
TE                    298.0 K
CNST2           145.0000000
CNST13           10.0000000
D0               0.00000300 sec
D1               1.50000000 sec
D2               0.00344828 sec
D6               0.05000000 sec
D16              0.00020000 sec
IN0              0.00001730 sec

======== CHANNEL f1 ========
SFO1            500.1324006 MHz
NUC1                     1H
P1                    10.40 usec
P2                    20.80 usec
PLW1             7.00000000 W

======== CHANNEL f2 ========
SFO2            125.7709936 MHz
NUC2                    13C
P3                    12.00 usec
PLW2           180.00000000 W

====== GRADIENT CHANNEL =====
GPNAM[1]        SMSQ10.100
GPNAM[2]        SMSQ10.100
GPNAM[3]        SMSQ10.100
GPZ1                  50.00 %
GPZ2                  30.00 %
GPZ3                  40.10 %
P16                 1000.00 usec

F1 - Acquisition parameters
TD                      256
SFO1                125.771 MHz
FIDRES           112.897400 Hz
SW                  229.796 ppm
FnMODE                   QF

F2 - Processing parameters
SI                     2048
SF              500.1300088 MHz
WDW                    SINE
SSB       0
LB        0 Hz
GB        0
PC                     1.40

F1 - Processing parameters
SI                     1024
MC2                      QF
SF              125.7577829 MHz
WDW                    SINE
SSB       0
LB        0 Hz
GB        0
```

DP-H-1 / HSQC

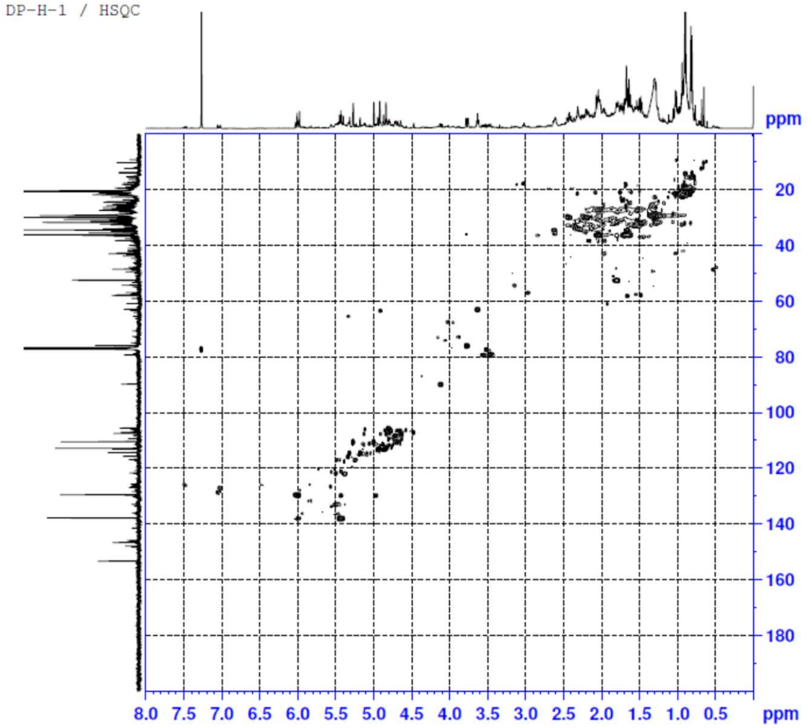

서 울 대 학 교
SEOUL NATIONAL UNIVERSITY
기초과학공동기기원
핵자기공명연구실

```
Current Data Parameters
NAME          dec03-kku-jin
EXPNO                     3
PROCNO                    1

F2 - Acquisition Parameters
Date_              20131203
Time                  22.29
INSTRUM               spect
PROBHD    5 mm Multinucl
PULPROG        hsqcetgpsi
TD                     2048
SOLVENT               CDCl3
NS                       32
DS                       16
SWH                5411.255 Hz
FIDRES             2.642215 Hz
AQ                0.1892352 sec
RG                     2050
DW                   92.400 usec
DE                     6.50 usec
TE                    298.0 K
CNST2           145.0000000
D0               0.00000300 sec
D1               1.50000000 sec
D4               0.00172414 sec
D11              0.03000000 sec
D16              0.00020000 sec
D24              0.00089000 sec
IN0              0.00001730 sec
ZGOPTNS

======== CHANNEL f1 ========
SFO1            500.1324006 MHz
NUC1                     1H
P1                    10.40 usec
P2                    20.80 usec
P28       0 usec
PLW1             7.00000000 W

======== CHANNEL f2 ========
SFO2            125.7709936 MHz
NUC2                    13C
CPDPRG[2 bi_p5m4sp_4sp.2
P3                    12.00 usec
P4                    24.00 usec
P63                 1500.00 usec
PLW2           180.00000000 W
PLW12            5.28980017 W
SPNAM[14 Crp32,1.5,20.2
SPOAL14               0.500
SPOFFS14 0 Hz
SPW14           16.89699936 W
SPNAM[31 Crp32,1.5,20.2
SPOAL31               0.500
SPOFFS31 0 Hz
SPW31            4.22429991 W

====== GRADIENT CHANNEL =====
GPNAM[1]        SMSQ10.100
GPNAM[2]        SMSQ10.100
GPZ1                  80.00 %
GPZ2                  20.10 %
P16                 1000.00 usec

F1 - Acquisition parameters
TD                      256
SFO1                125.771 MHz
FIDRES           112.897400 Hz
SW                  229.796 ppm
FnMODE     Echo-Antiecho

F2 - Processing parameters
SI                     1024
SF              500.1300067 MHz
WDW                   QSINE
SSB                       2
LB        0 Hz
GB        0
PC                     1.40

F1 - Processing parameters
SI                     1024
MC2         echo-antiecho
SF              125.7577837 MHz
WDW                   QSINE
SSB                       2
LB        0 Hz
GB        0
```

DP-H-1 / HSQC

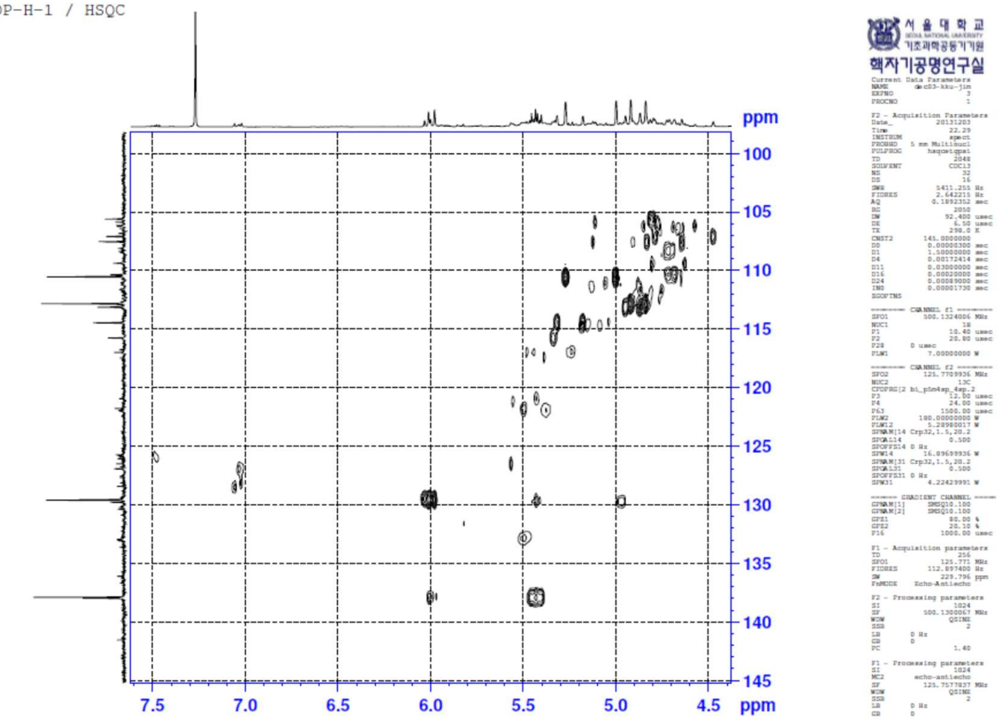

DP-H-1 / HSQC

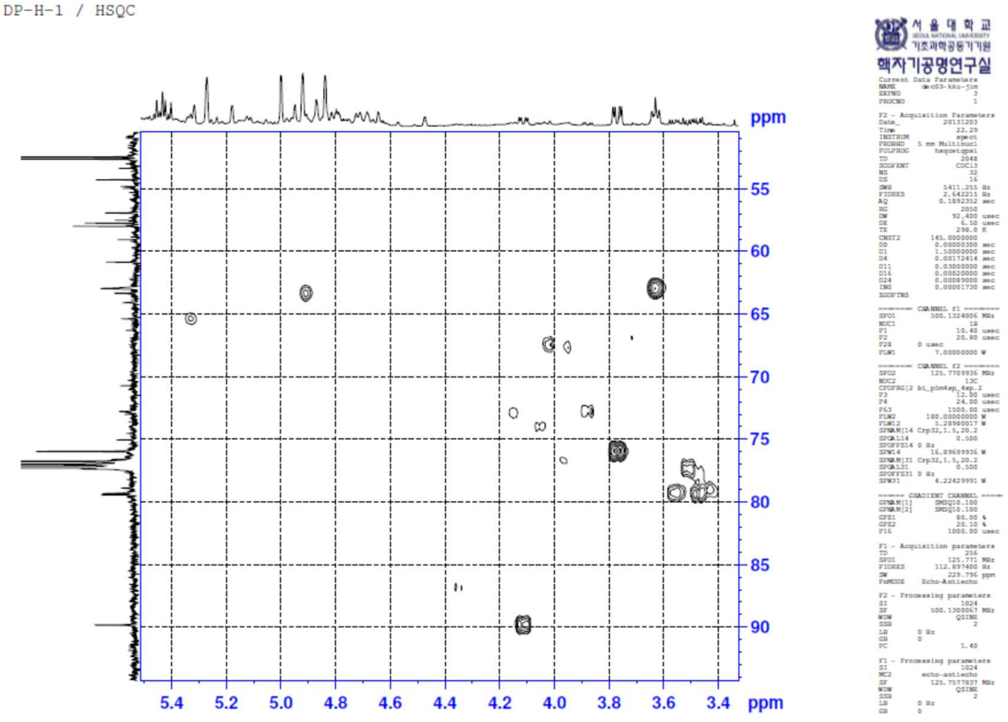

DP-H-1 / HSQC

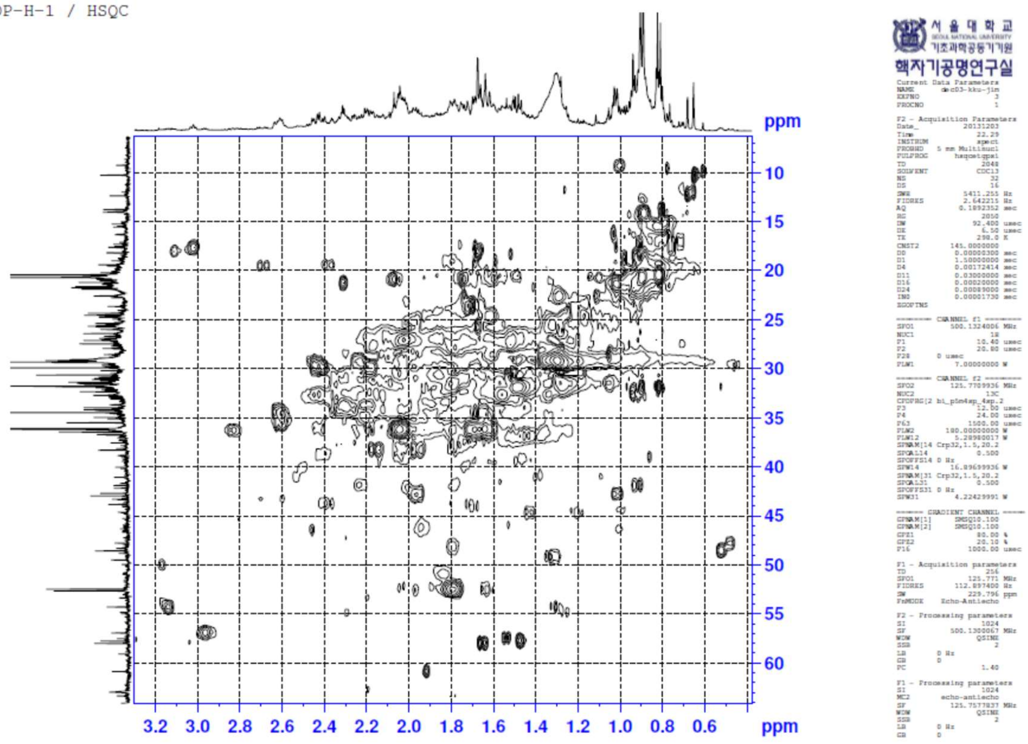

DP-H-1 / COSY

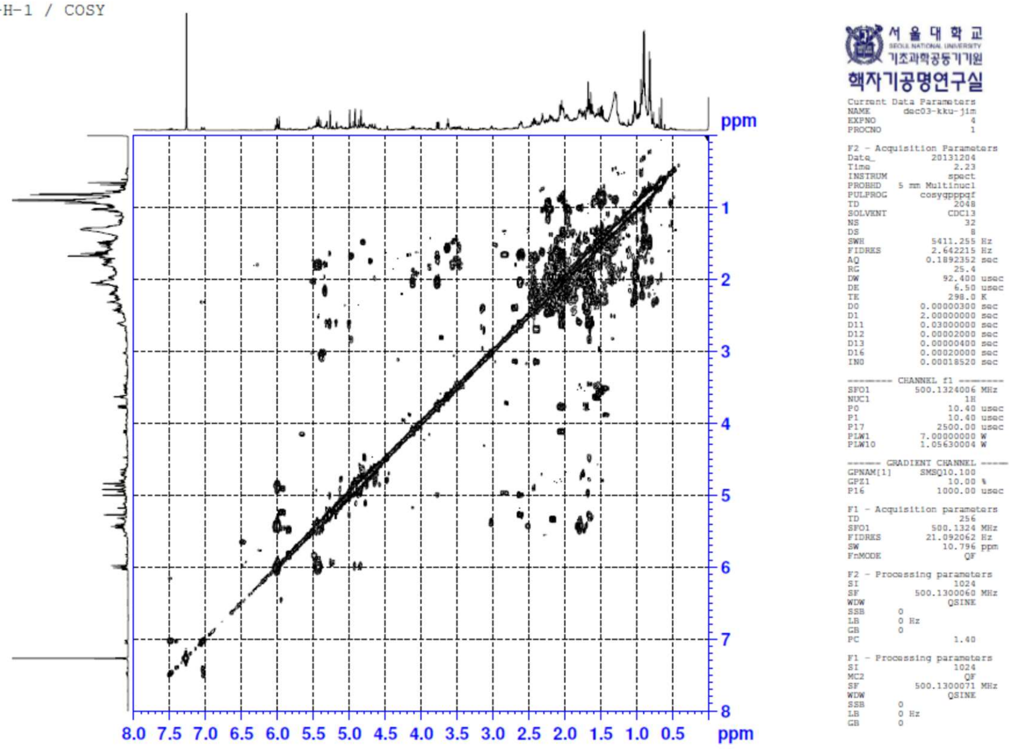

DP-H-1 / COSY

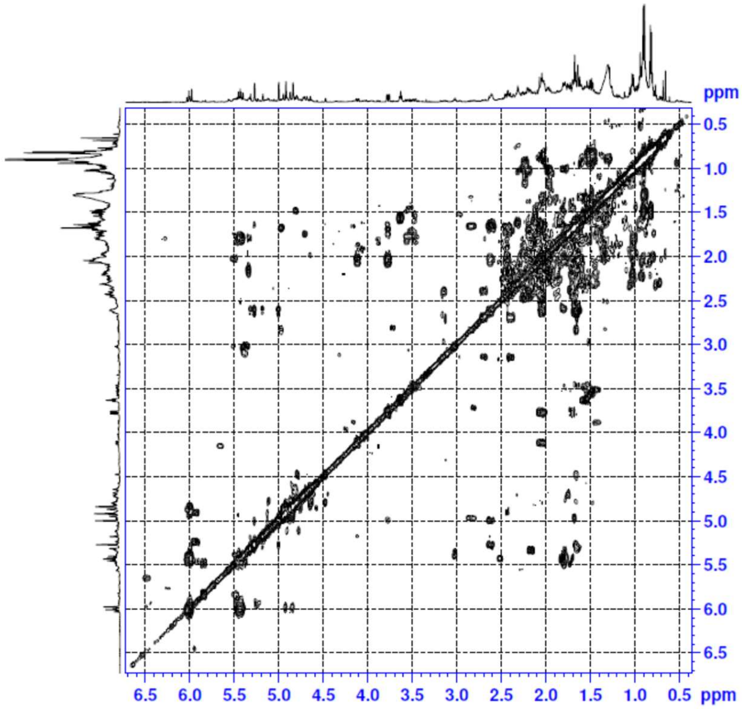

서 울 대 학 교
SEOUL NATIONAL UNIVERSITY
기초과학공동기기원
핵자기공명연구실

```
Current Data Parameters
NAME        dec03-kku-jim
EXPNO                   4
PROCNO                  1

F2 - Acquisition Parameters
Date_            20131204
Time                 2.23
INSTRUM             spect
PROBHD   5 mm Multinucl
PULPROG        cosygpppqf
TD                   2048
SOLVENT             CDCl3
NS                     32
DS                      8
SWH             5411.255 Hz
FIDRES          2.642215 Hz
AQ             0.1892352 sec
RG                   25.4
DW                 92.400 usec
DE                   6.50 usec
TE                  298.0 K
D0             0.00000300 sec
D1             2.00000000 sec
D11            0.03000000 sec
D12            0.00002000 sec
D13            0.00000400 sec
D16            0.00020000 sec
IN0            0.00018520 sec

======== CHANNEL f1 ========
SFO1          500.1324006 MHz
NUC1                   1H
P0                  10.40 usec
P1                  10.40 usec
P17               2500.00 usec
PLW1           7.00000000 W
PLW10          1.05630004 W

====== GRADIENT CHANNEL =====
GPNAM[1]       SMSQ10.100
GPZ1                10.00 %
P16               1000.00 usec

F1 - Acquisition parameters
TD                    256
SFO1             500.1324 MHz
FIDRES          21.092062 Hz
SW                 10.796 ppm
FnMODE                 QF

F2 - Processing parameters
SI                   1024
SF            500.1300060 MHz
WDW                 QSINE
SSB      0
LB       0 Hz
GB       0
PC                   1.40

F1 - Processing parameters
SI                   1024
MC2                    QF
SF            500.1300071 MHz
WDW                 QSINE
SSB      0
LB       0 Hz
GB       0
```

[Mass Spectrum]
Data : FAB-H079 Date : 13-Dec-2013 15:04
Sample: DP-H-1
Note : m-NBA
Inlet : Direct Ion Mode : FAB+
Spectrum Type : Normal Ion [MF-Linear]
RT : 2.71 min Scan# : (13,16)
BP : m/z 203.0000 Int. : 154.09
Output m/z range : 10.0000 to 400.0593 Cut Level : 0.00 %

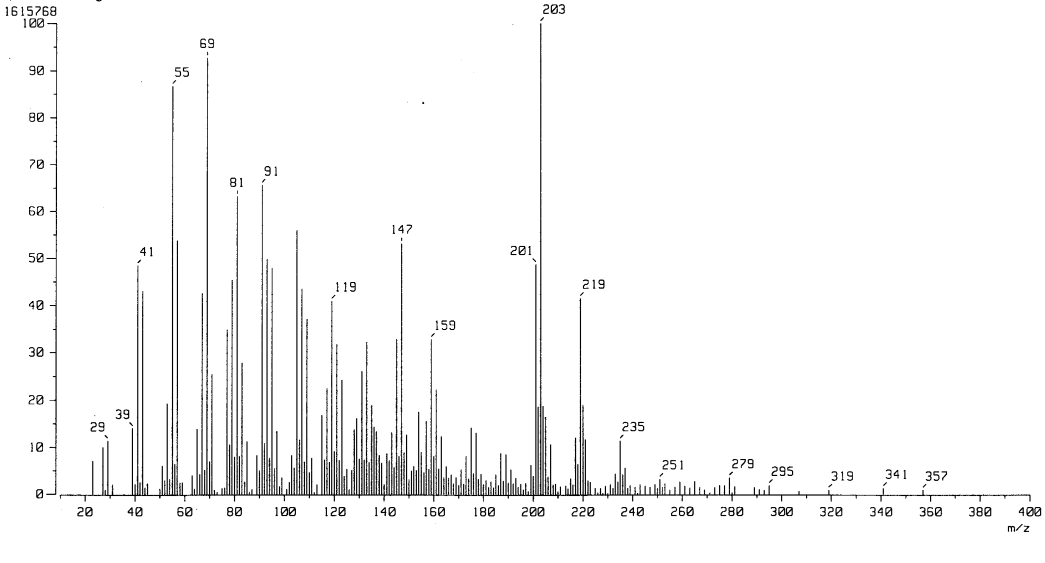

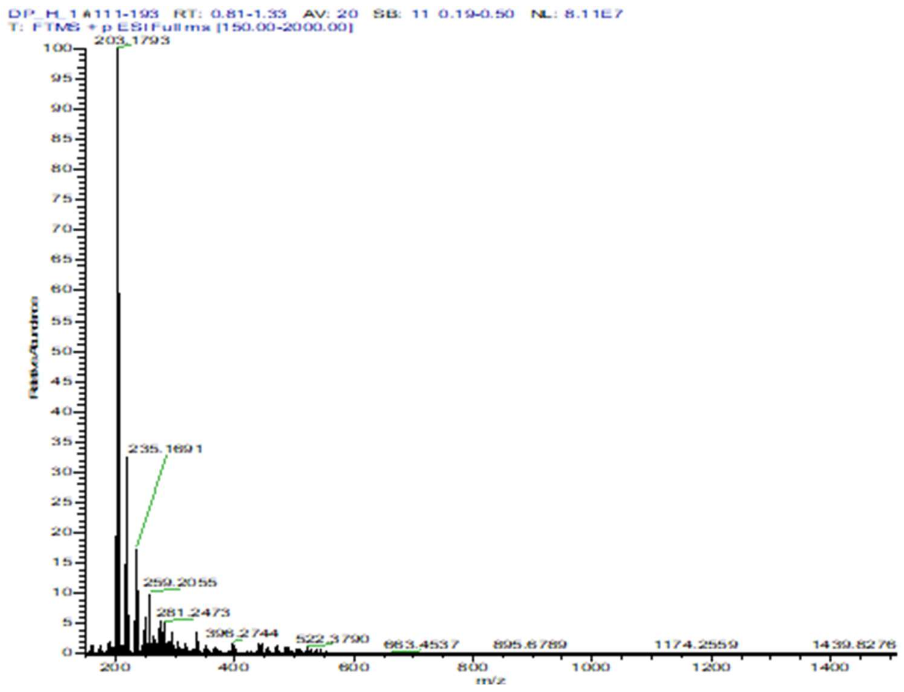
DP_H_1 #111-193 RT: 0.81-1.33 AV: 20 SB: 11 0.19-0.50 NL: 8.11E7
T: FTMS + p ESI Full ms [150.00-2000.00]
203.1793
235.1691
259.2055
281.2473
396.2744
522.3790
663.4537
895.6789
1174.2559
1439.8276
Relative Abundance
m/z

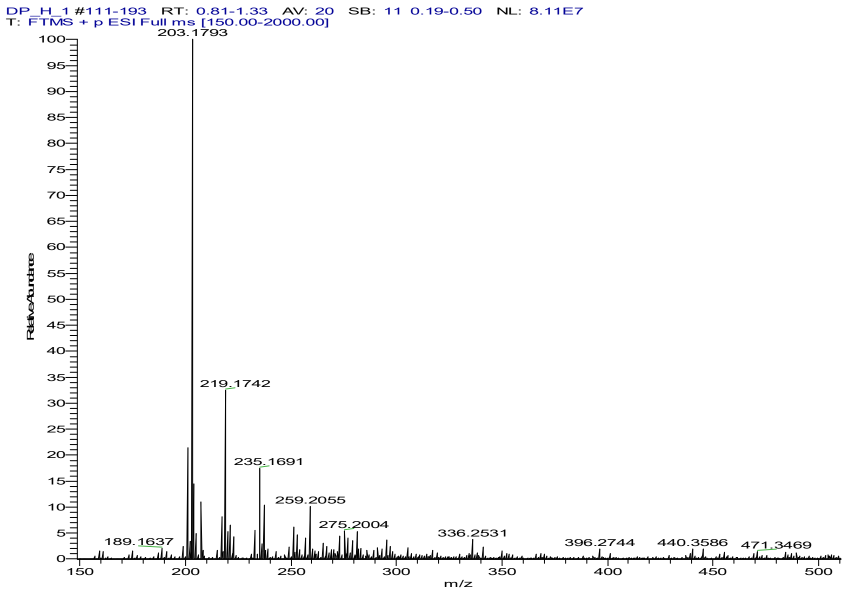
DP_H_1 #111-193 RT: 0.81-1.33 AV: 20 SB: 11 0.19-0.50 NL: 8.11E7
T: FTMS + p ESI Full ms [150.00-2000.00]
203.1793
219.1742
235.1691
259.2055
275.2004
336.2531
189.1637
396.2744
440.3586
471.3469
Relative Abundance
m/z

Compound 3

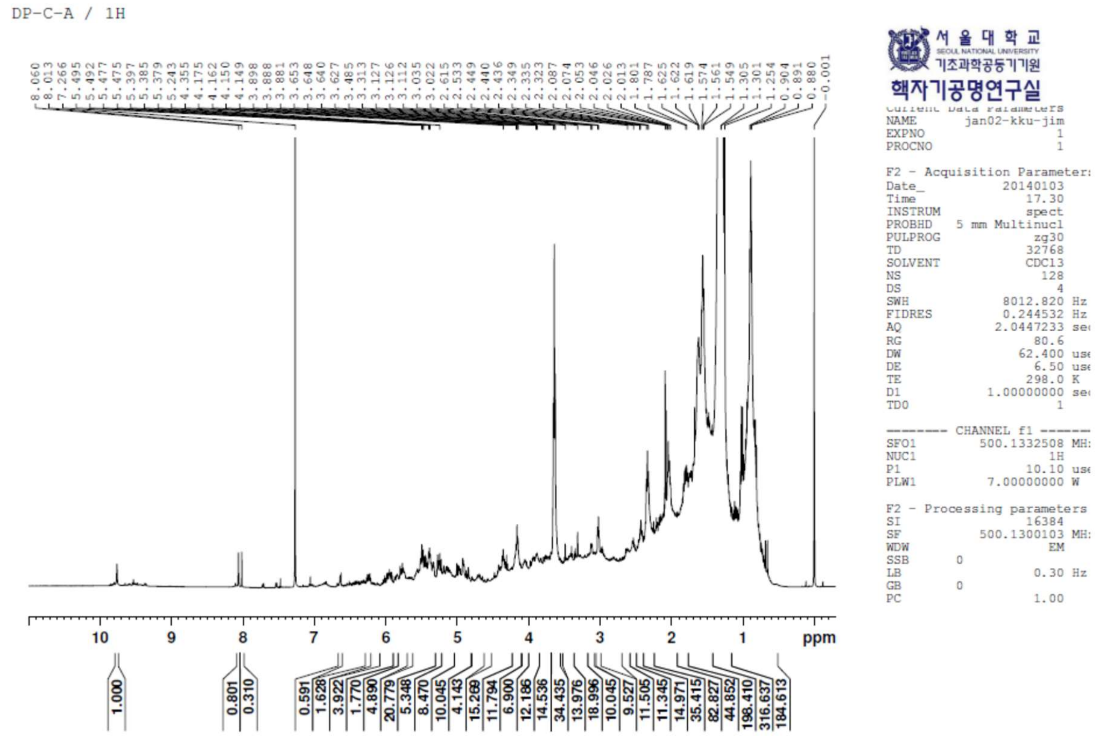
DP-C-A / 1H
NAME jan02-kku-jim
EXPNO 1
PROCNO 1
F2 - Acquisition Parameters
Date_ 20140103
Time 17.30
INSTRUM spect
PROBHD 5 mm Multinucl
PULPROG zg30
TD 32768
SOLVENT CDCl3
NS 128
DS 4
SWH 8012.820 Hz
FIDRES 0.244532 Hz
AQ 2.0447233 sec
RG 80.6
DW 62.400 usec
DE 6.50 usec
TE 298.0 K
D1 1.00000000 sec
TD0 1
CHANNEL f1
SFO1 500.1332508 MHz
NUC1 1H
P1 10.10 usec
PLW1 7.00000000 W
F2 - Processing parameters
SI 16384
SF 500.1300103 MHz
WDW EM
SSB 0
LB 0.30 Hz
GB 0
PC 1.00
10 9 8 7 6 5 4 3 2 1 ppm
1.000
0.801
0.310
0.591
1.628
3.922
1.770
4.890
20.779
5.348
8.470
10.045
4.143
15.269
11.794
6.900
12.186
14.536
34.435
13.976
18.996
10.045
9.527
11.505
11.345
14.971
35.415
82.827
44.852
198.410
316.637
184.613

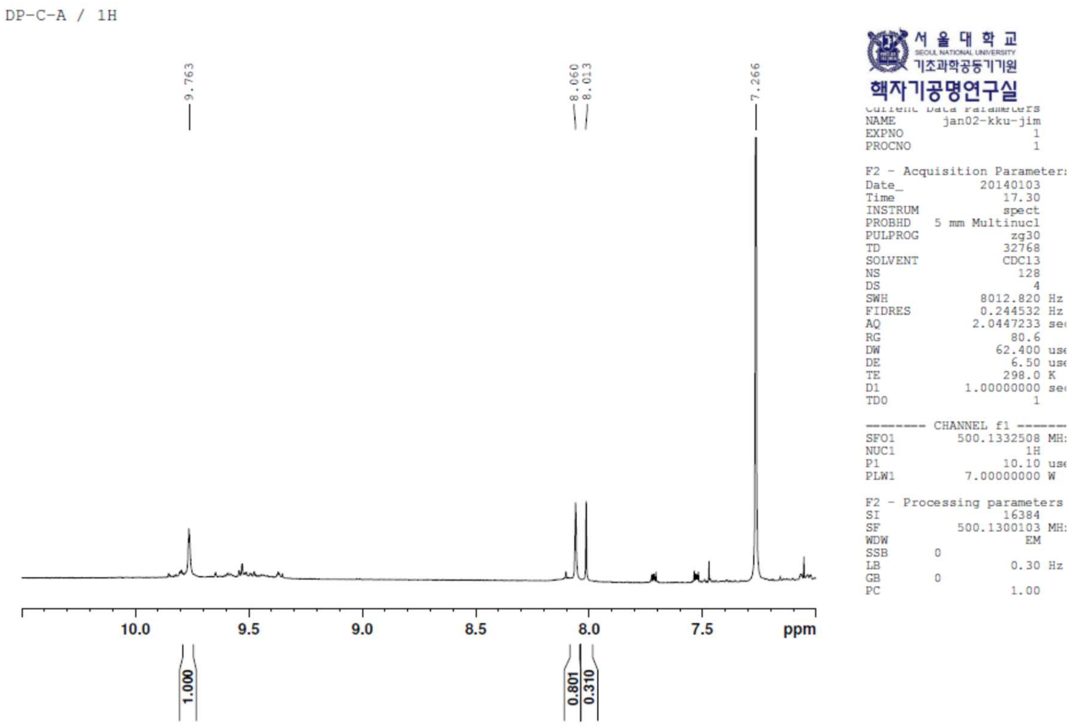
DP-C-A / 1H
9.763
8.060
8.013
7.266
NAME jan02-kku-jim
EXPNO 1
PROCNO 1
F2 - Acquisition Parameters
Date_ 20140103
Time 17.30
INSTRUM spect
PROBHD 5 mm Multinucl
PULPROG zg30
TD 32768
SOLVENT CDCl3
NS 128
DS 4
SWH 8012.820 Hz
FIDRES 0.244532 Hz
AQ 2.0447233 sec
RG 80.6
DW 62.400 usec
DE 6.50 usec
TE 298.0 K
D1 1.00000000 sec
TD0 1
CHANNEL f1
SFO1 500.1332508 MHz
NUC1 1H
P1 10.10 usec
PLW1 7.00000000 W
F2 - Processing parameters
SI 16384
SF 500.1300103 MHz
WDW EM
SSB 0
LB 0.30 Hz
GB 0
PC 1.00
10.0 9.5 9.0 8.5 8.0 7.5 ppm
1.000
0.801
0.310

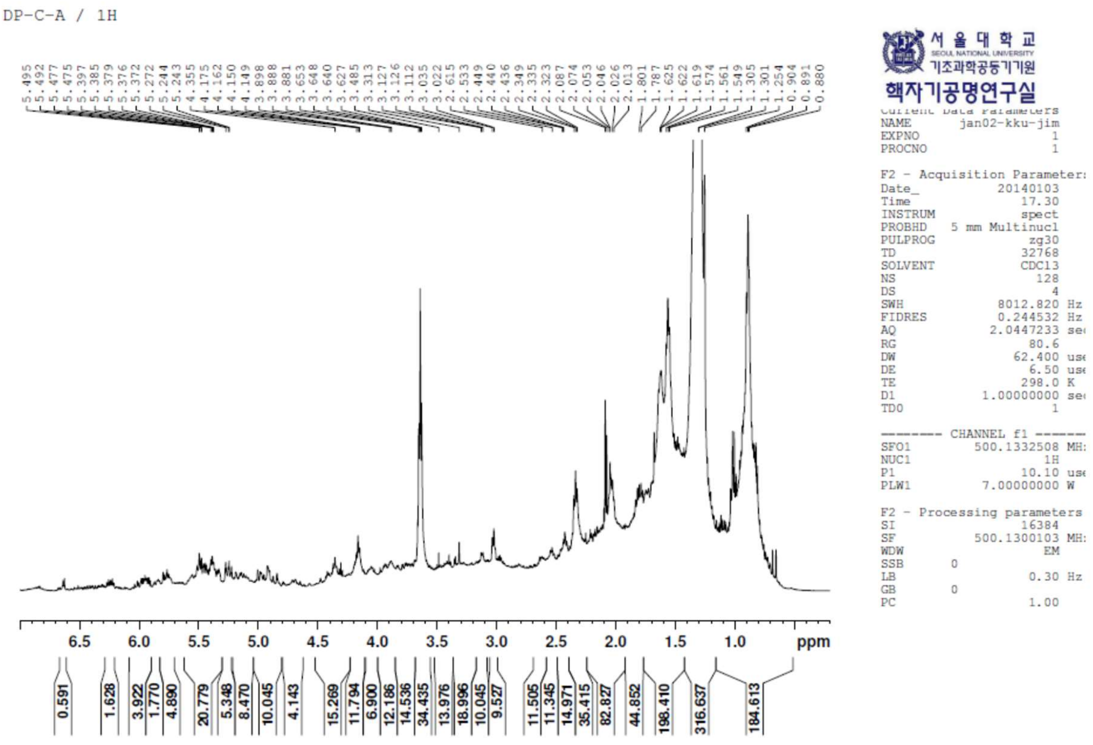
DP-C-A / 1H
서 울 대 학 교
SEOUL NATIONAL UNIVERSITY
기초과학공동기기원
핵자기공명연구실
NAME jan02-kku-jim
EXPNO 1
PROCNO 1
F2 - Acquisition Parameter:
Date_ 20140103
Time 17.30
INSTRUM spect
PROBHD 5 mm Multinucl
PULPROG zg30
TD 32768
SOLVENT CDCl3
NS 128
DS 4
SWH 8012.820 Hz
FIDRES 0.244532 Hz
AQ 2.0447233 se
RG 80.6
DW 62.400 us
DE 6.50 us
TE 298.0 K
D1 1.00000000 se
TD0 1
CHANNEL f1
SFO1 500.1332508 MH
NUC1 1H
P1 10.10 us
PLW1 7.00000000 W
F2 - Processing parameters
SI 16384
SF 500.1300103 MH
WDW EM
SSB 0
LB 0.30 Hz
GB 0
PC 1.00
ppm

13C NMR

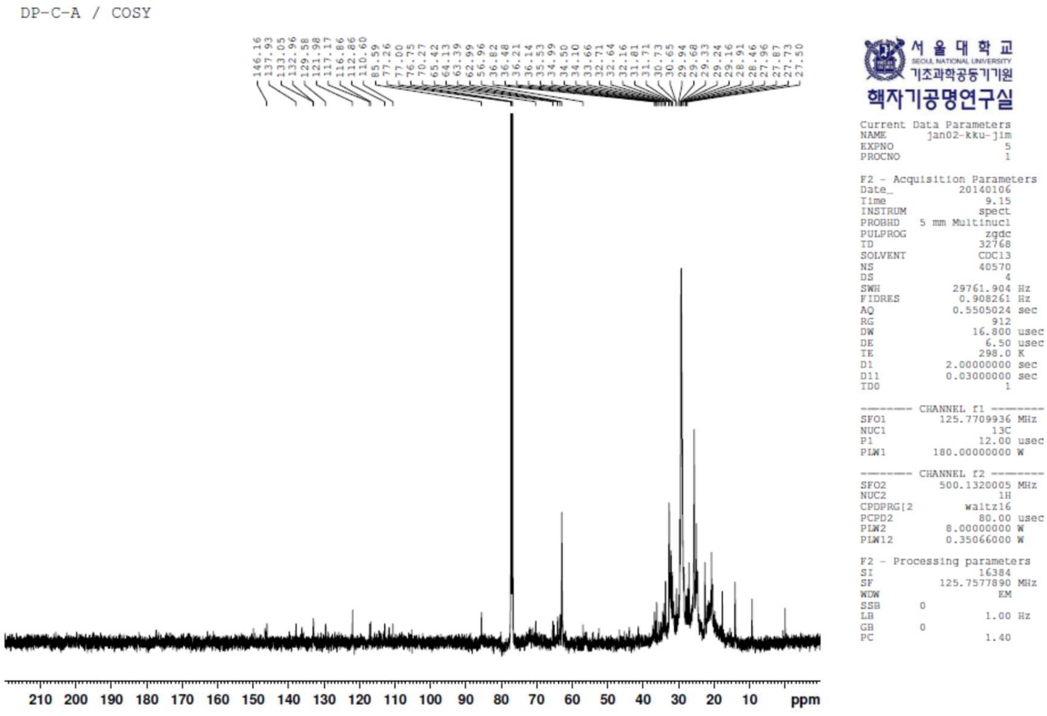
DP-C-A / COSY
서 울 대 학 교
SEOUL NATIONAL UNIVERSITY
기초과학공동기기원
핵자기공명연구실
Current Data Parameters
NAME jan02-kku-jim
EXPNO 5
PROCNO 1
F2 - Acquisition Parameters
Date_ 20140106
Time 9.15
INSTRUM spect
PROBHD 5 mm Multinucl
PULPROG zgdc
TD 32768
SOLVENT CDCl3
NS 40570
DS 4
SWH 29761.904 Hz
FIDRES 0.908261 Hz
AQ 0.5505024 sec
RG 912
DW 16.800 usec
DE 6.50 usec
TE 298.0 K
D1 2.00000000 sec
D11 0.03000000 sec
TD0 1
CHANNEL f1
SFO1 125.7709936 MHz
NUC1 13C
P1 12.00 usec
PLW1 180.00000000 W
CHANNEL f2
SFO2 500.1320005 MHz
NUC2 1H
CPDPRG[2 waltz16
PCPD2 80.00 usec
PLW2 8.00000000 W
PLW12 0.35066000 W
F2 - Processing parameters
SI 16384
SF 125.7577890 MHz
WDW EM
SSB 0
LB 1.00 Hz
GB 0
PC 1.40
ppm

DP-C-A / COSY

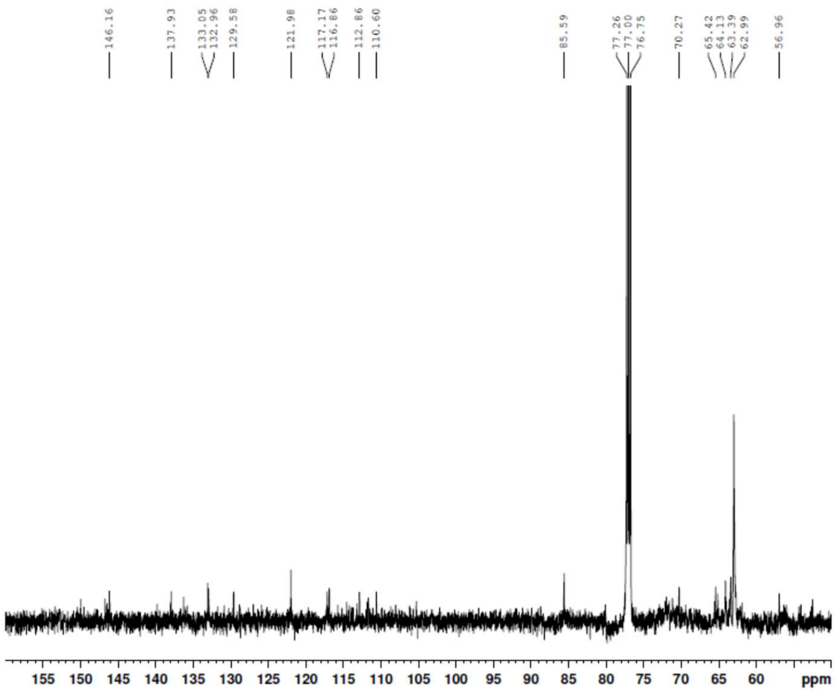

서울대학교
SEOUL NATIONAL UNIVERSITY
기초과학공동기기원
핵자기공명연구실

```
Current Data Parameters
NAME         jan02-kku-jim
EXPNO                    5
PROCNO                   1

F2 - Acquisition Parameters
Date_             20140106
Time                  9.15
INSTRUM              spect
PROBHD   5 mm Multinucl
PULPROG               zgdc
TD                   32768
SOLVENT              CDCl3
NS                   40570
DS                       4
SWH              29761.904 Hz
FIDRES            0.908261 Hz
AQ               0.5505024 sec
RG                     912
DW                  16.800 usec
DE                    6.50 usec
TE                   298.0 K
D1              2.00000000 sec
D11             0.03000000 sec
TD0                      1

======== CHANNEL f1 ========
SFO1           125.7709936 MHz
NUC1                   13C
P1                   12.00 usec
PLW1          180.00000000 W

======== CHANNEL f2 ========
SFO2           500.1320005 MHz
NUC2                    1H
CPDPRG[2           waltz16
PCPD2                80.00 usec
PLW2            8.00000000 W
PLW12           0.35066000 W

F2 - Processing parameters
SI                   16384
SF             125.7577890 MHz
WDW                     EM
SSB      0
LB                    1.00 Hz
GB       0
PC                    1.40
```

DP-C-A / COSY

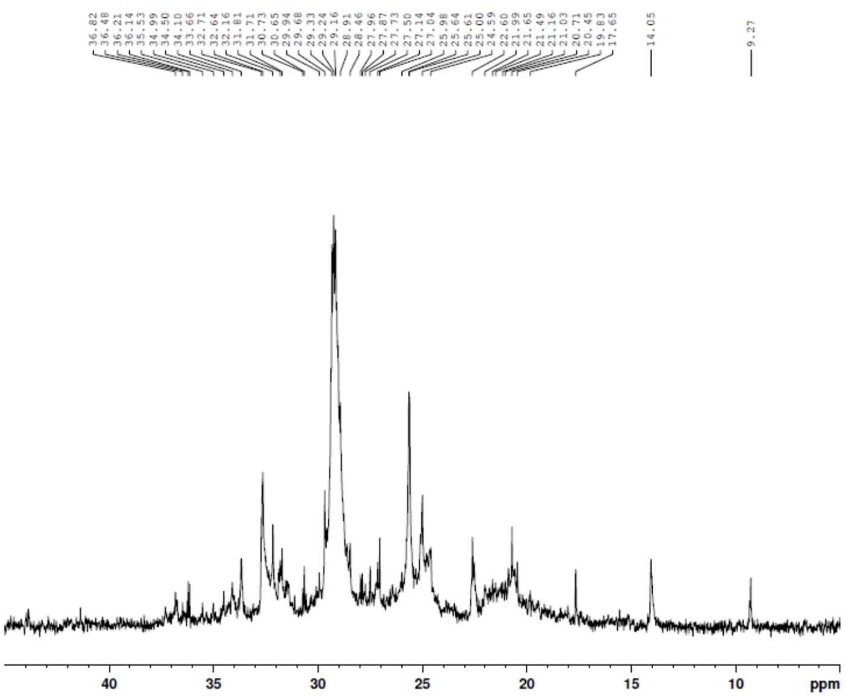

서울대학교
SEOUL NATIONAL UNIVERSITY
기초과학공동기기원
핵자기공명연구실

```
Current Data Parameters
NAME         jan02-kku-jim
EXPNO                    5
PROCNO                   1

F2 - Acquisition Parameters
Date_             20140106
Time                  9.15
INSTRUM              spect
PROBHD   5 mm Multinucl
PULPROG               zgdc
TD                   32768
SOLVENT              CDCl3
NS                   40570
DS                       4
SWH              29761.904 Hz
FIDRES            0.908261 Hz
AQ               0.5505024 sec
RG                     912
DW                  16.800 usec
DE                    6.50 usec
TE                   298.0 K
D1              2.00000000 sec
D11             0.03000000 sec
TD0                      1

======== CHANNEL f1 ========
SFO1           125.7709936 MHz
NUC1                   13C
P1                   12.00 usec
PLW1          180.00000000 W

======== CHANNEL f2 ========
SFO2           500.1320005 MHz
NUC2                    1H
CPDPRG[2           waltz16
PCPD2                80.00 usec
PLW2            8.00000000 W
PLW12           0.35066000 W

F2 - Processing parameters
SI                   16384
SF             125.7577890 MHz
WDW                     EM
SSB      0
LB                    1.00 Hz
GB       0
PC                    1.40
```

DP-C-A / COSY

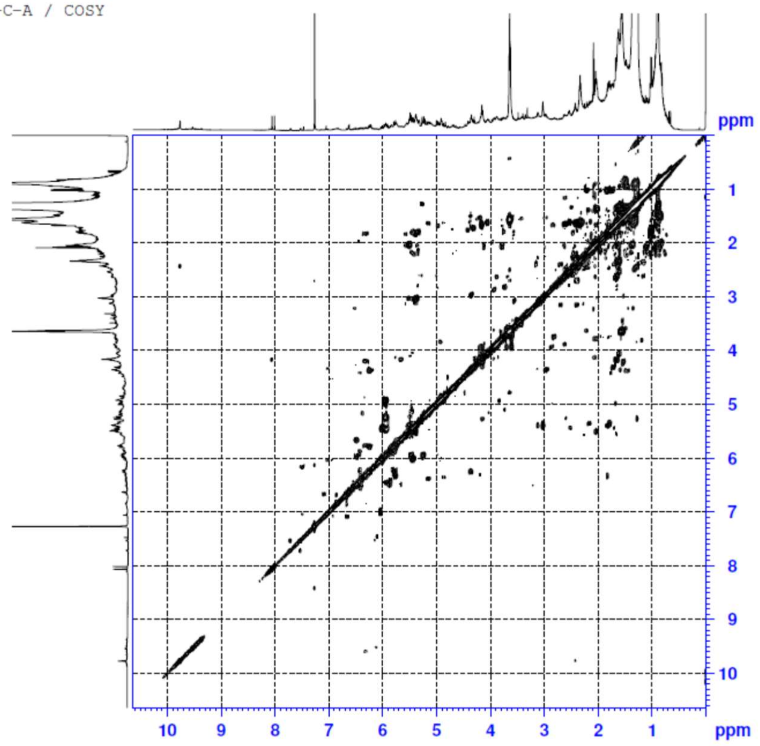

서울대학교
SEOUL NATIONAL UNIVERSITY
기초과학공동기기원
핵자기공명연구실

```
Current Data Parameters
NAME        jan02-kku-jim
EXPNO                   4
PROCNO                  1

F2 - Acquisition Parameters
Date_            20140104
Time                18.51
INSTRUM             spect
PROBHD   5 mm Multinucl
PULPROG        cosygpppqf
TD                   2048
SOLVENT             CDCl3
NS                     64
DS                      8
SWH              5647.590 Hz
FIDRES           2.757612 Hz
AQ              0.1813163 sec
RG                   25.4
DW                 88.533 usec
DE                   6.50 usec
TE                  298.0 K
D0             0.00000300 sec
D1             2.00000000 sec
D11            0.03000000 sec
D12            0.00002000 sec
D13            0.00000400 sec
D16            0.00020000 sec
IN0            0.00017700 sec

======== CHANNEL f1 ========
SFO1          500.1325007 MHz
NUC1                   1H
P0                  10.80 usec
P1                  10.80 usec
P17               2500.00 usec
PLW1           7.00000000 W
PLW10          1.05630004 W

====== GRADIENT CHANNEL =====
GPNAM[1]       SMSQ10.100
GPZ1                10.00 %
P16               1000.00 usec

F1 - Acquisition parameters
TD                    256
SFO1             500.1325 MHz
FIDRES          22.069208 Hz
SW                 11.296 ppm
FnMODE                 QF

F2 - Processing parameters
SI                   1024
SF            500.1300066 MHz
WDW                 QSINE
SSB       0
LB        0 Hz
GB        0
PC                   1.40

F1 - Processing parameters
SI                   1024
MC2                    QF
SF            500.1300067 MHz
WDW                 QSINE
SSB       0
LB        0 Hz
GB        0
```

DP-C-A / COSY

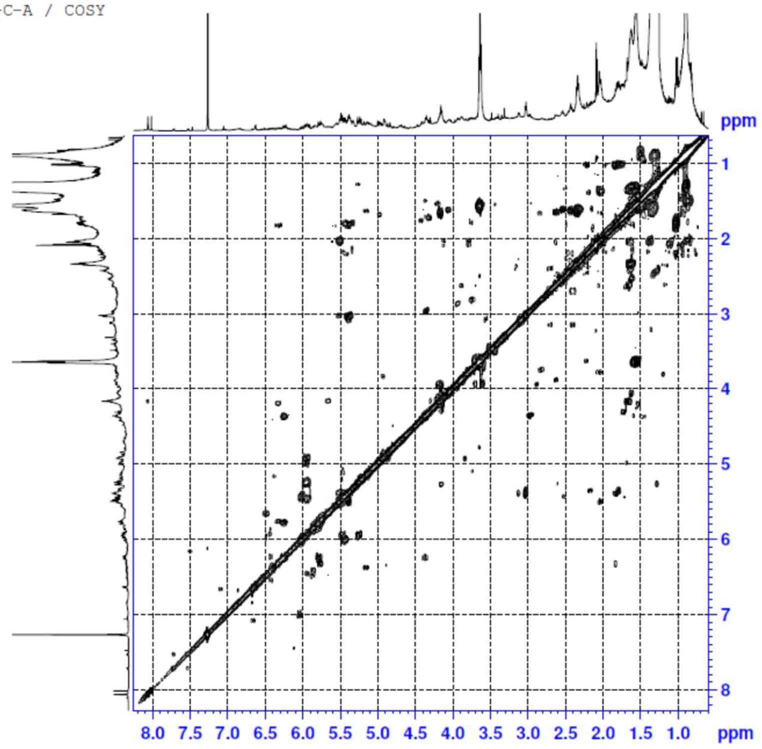

서울대학교
SEOUL NATIONAL UNIVERSITY
기초과학공동기기원
핵자기공명연구실

```
Current Data Parameters
NAME        jan02-kku-jim
EXPNO                   4
PROCNO                  1

F2 - Acquisition Parameters
Date_            20140104
Time                18.51
INSTRUM             spect
PROBHD   5 mm Multinucl
PULPROG        cosygpppqf
TD                   2048
SOLVENT             CDCl3
NS                     64
DS                      8
SWH              5647.590 Hz
FIDRES           2.757612 Hz
AQ              0.1813163 sec
RG                   25.4
DW                 88.533 usec
DE                   6.50 usec
TE                  298.0 K
D0             0.00000300 sec
D1             2.00000000 sec
D11            0.03000000 sec
D12            0.00002000 sec
D13            0.00000400 sec
D16            0.00020000 sec
IN0            0.00017700 sec

======== CHANNEL f1 ========
SFO1          500.1325007 MHz
NUC1                   1H
P0                  10.80 usec
P1                  10.80 usec
P17               2500.00 usec
PLW1           7.00000000 W
PLW10          1.05630004 W

====== GRADIENT CHANNEL =====
GPNAM[1]       SMSQ10.100
GPZ1                10.00 %
P16               1000.00 usec

F1 - Acquisition parameters
TD                    256
SFO1             500.1325 MHz
FIDRES          22.069208 Hz
SW                 11.296 ppm
FnMODE                 QF

F2 - Processing parameters
SI                   1024
SF            500.1300066 MHz
WDW                 QSINE
SSB       0
LB        0 Hz
GB        0
PC                   1.40

F1 - Processing parameters
SI                   1024
MC2                    QF
SF            500.1300067 MHz
WDW                 QSINE
SSB       0
LB        0 Hz
GB        0
```

DP-C-A / COSY

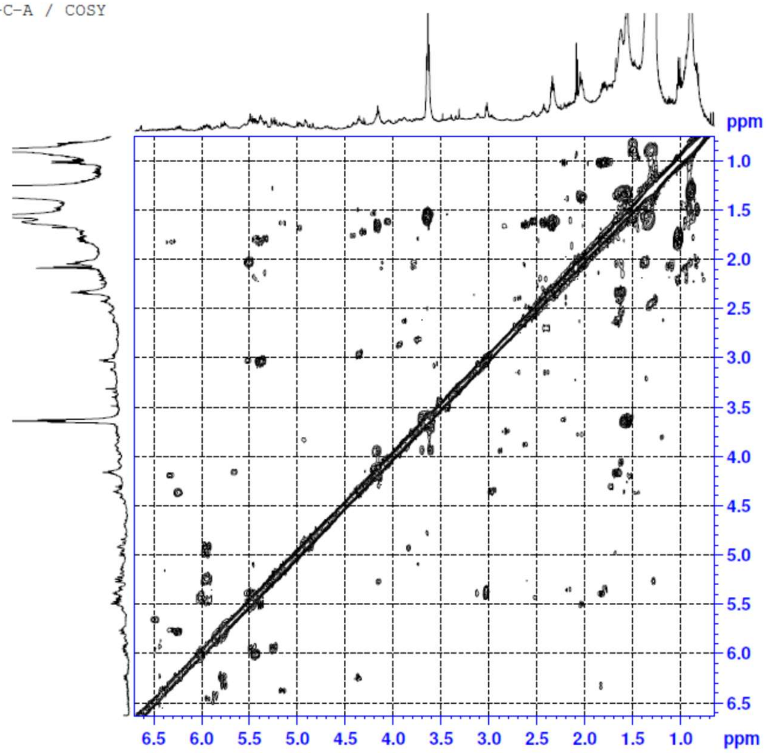

서울대학교
SEOUL NATIONAL UNIVERSITY
기초과학공동기기원
핵자기공명연구실

```
Current Data Parameters
NAME          jan02-kku-jim
EXPNO                     4
PROCNO                    1

F2 - Acquisition Parameters
Date_              20140104
Time                  18.51
INSTRUM               spect
PROBHD   5 mm Multinucl
PULPROG          cosygpppqf
TD                     2048
SOLVENT               CDCl3
NS                       64
DS                        8
SWH                5647.590 Hz
FIDRES             2.757612 Hz
AQ                0.1813163 sec
RG                     25.4
DW                   88.533 usec
DE                     6.50 usec
TE                    298.0 K
D0               0.00000300 sec
D1               2.00000000 sec
D11              0.03000000 sec
D12              0.00002000 sec
D13              0.00000400 sec
D16              0.00020000 sec
IN0              0.00017700 sec

======== CHANNEL f1 ========
SFO1            500.1325007 MHz
NUC1                     1H
P0                    10.80 usec
P1                    10.80 usec
P17                 2500.00 usec
PLW1             7.00000000 W
PLW10            1.05630004 W

====== GRADIENT CHANNEL =====
GPNAM[1]         SMSQ10.100
GPZ1                  10.00 %
P16                 1000.00 usec

F1 - Acquisition parameters
TD                      256
SFO1               500.1325 MHz
FIDRES            22.069208 Hz
SW                   11.296 ppm
FnMODE                   QF

F2 - Processing parameters
SI                     1024
SF              500.1300066 MHz
WDW                   QSINE
SSB        0
LB         0 Hz
GB         0
PC                     1.40

F1 - Processing parameters
SI                     1024
MC2                      QF
SF              500.1300067 MHz
WDW                   QSINE
SSB        0
LB         0 Hz
GB         0
```

DP-C-A / HSQC

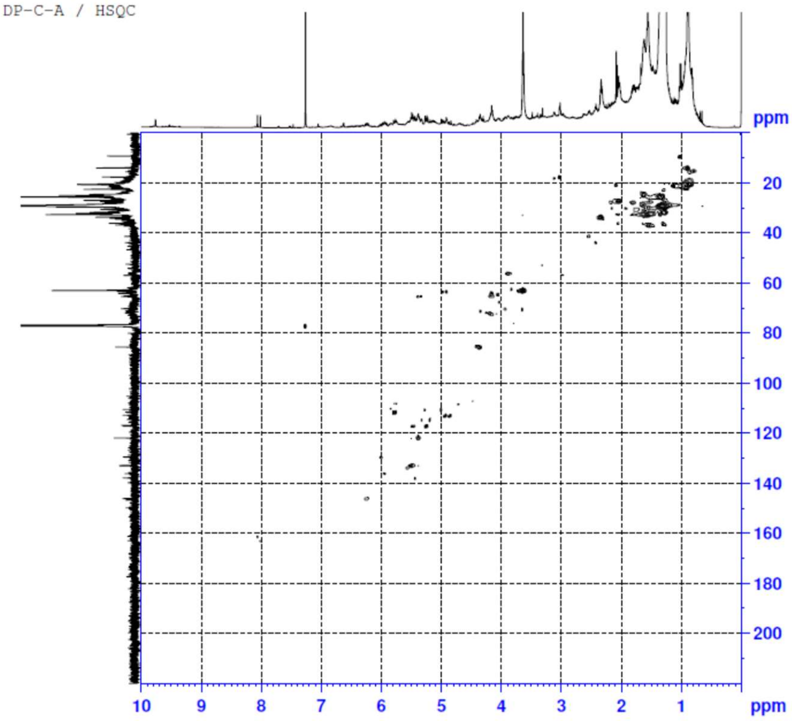

서울대학교
SEOUL NATIONAL UNIVERSITY
기초과학공동기기원
핵자기공명연구실

```
Current Data Parameters
NAME          jan02-kku-jim
EXPNO                     3
PROCNO                    1

F2 - Acquisition Parameters
Date_              20140104
Time                  11.06
INSTRUM               spect
PROBHD   5 mm Multinucl
PULPROG          hsqcetgpsi
TD                     2048
SOLVENT               CDCl3
NS                       64
DS                       16
SWH                5647.590 Hz
FIDRES             2.757612 Hz
AQ                0.1813163 sec
RG                     2050
DW                   88.533 usec
DE                     6.50 usec
TE                    298.0 K
CNST2           145.0000000
D0               0.00000300 sec
D1               1.50000000 sec
D4               0.00172414 sec
D11              0.03000000 sec
D16              0.00020000 sec
D24              0.00089000 sec
IN0              0.00001730 sec
ZGOPTNS

======== CHANNEL f1 ========
SFO1            500.1325007 MHz
NUC1                     1H
P1                    10.80 usec
P2                    21.60 usec
P28        0 usec
PLW1             7.00000000 W

======== CHANNEL f2 ========
SFO2            125.7709936 MHz
NUC2                    13C
CPDPRG[2 bi_p5m4sp_4sp.2
P3                    12.00 usec
P4                    24.00 usec
P63                 1500.00 usec
PLW2           180.00000000 W
PLW12            5.28980017 W
SPNAM[14 Crp32,1.5,20.2
SPOAL14               0.500
SPOFFS14 0 Hz
SPW14           16.89699936 W
SPNAM[31 Crp32,1.5,20.2
SPOAL31               0.500
SPOFFS31 0 Hz
SPW31            4.22429991 W

====== GRADIENT CHANNEL =====
GPNAM[1]         SMSQ10.100
GPNAM[2]         SMSQ10.100
GPZ1                  80.00 %
GPZ2                  20.10 %
P16                 1000.00 usec

F1 - Acquisition parameters
TD                      256
SFO1                125.771 MHz
FIDRES           112.897400 Hz
SW                  229.796 ppm
FnMODE     Echo-Antiecho

F2 - Processing parameters
SI                     1024
SF              500.1300071 MHz
WDW                   QSINE
SSB                       2
LB         0 Hz
GB         0
PC                     1.40

F1 - Processing parameters
SI                     1024
MC2           echo-antiecho
SF              125.7577782 MHz
WDW                   QSINE
SSB                       2
LB         0 Hz
GB         0
```

DP-C-A / HSQC

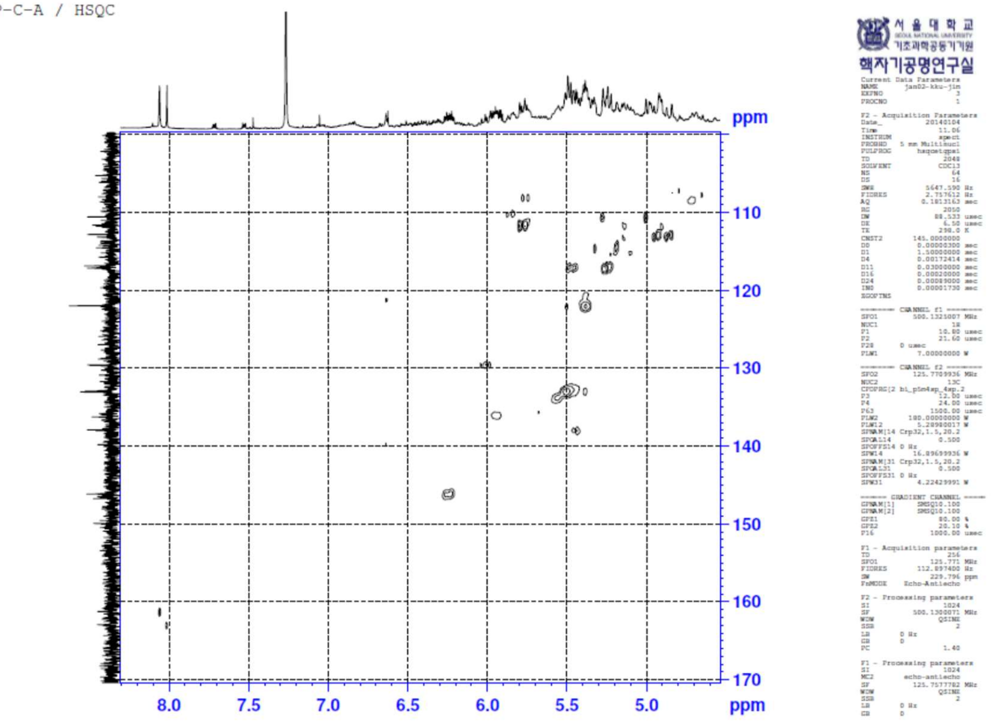

DP-C-A / HSQC

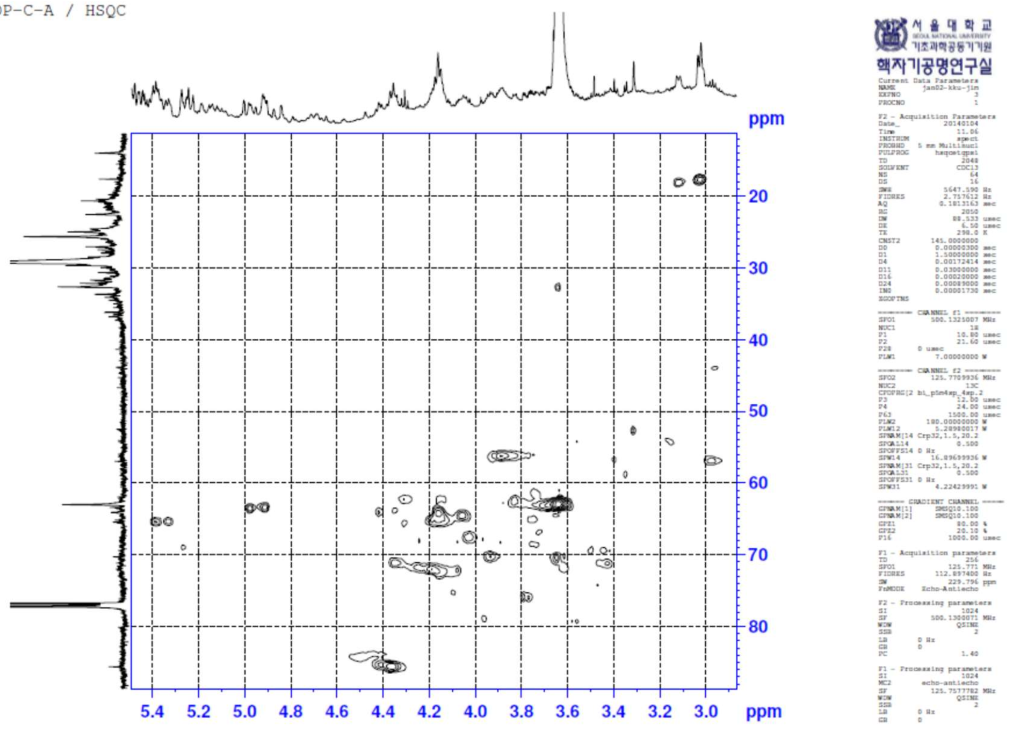

DP-C-A / HSQC

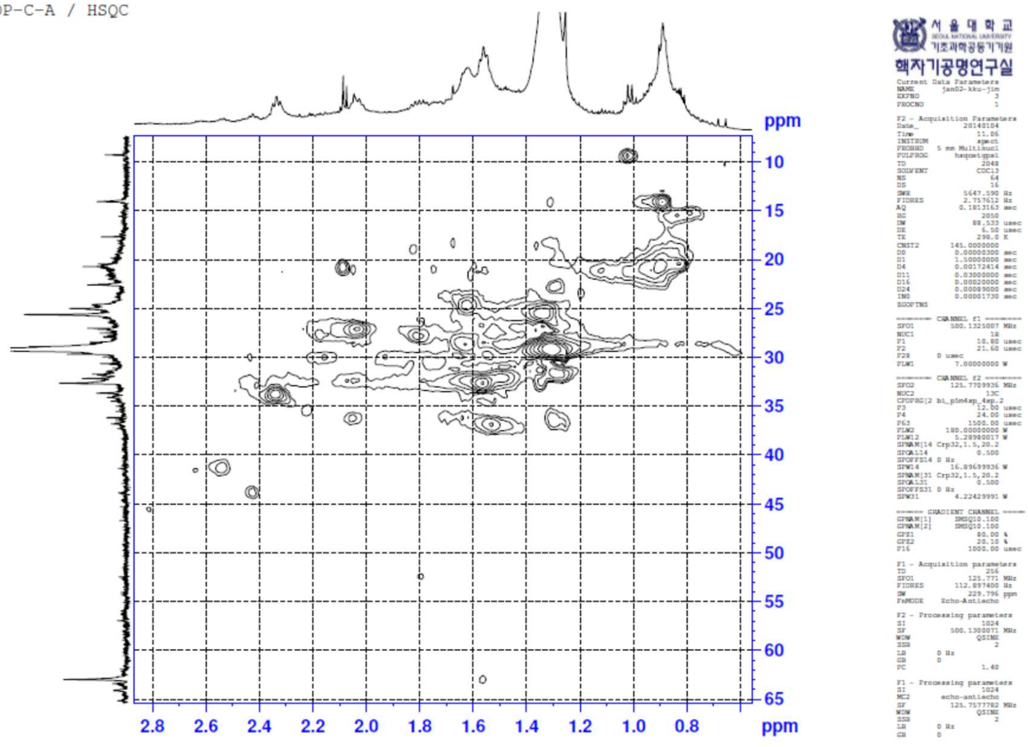

DP-C-A / HMBC

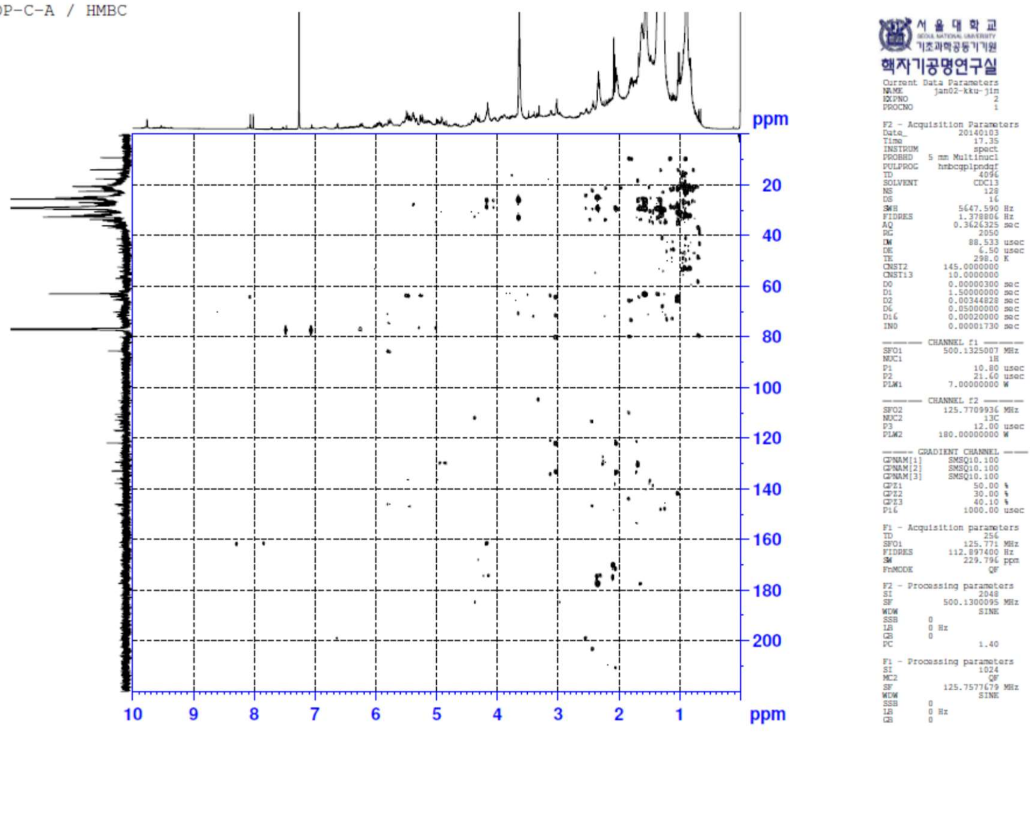

DP-C-A / HMBC

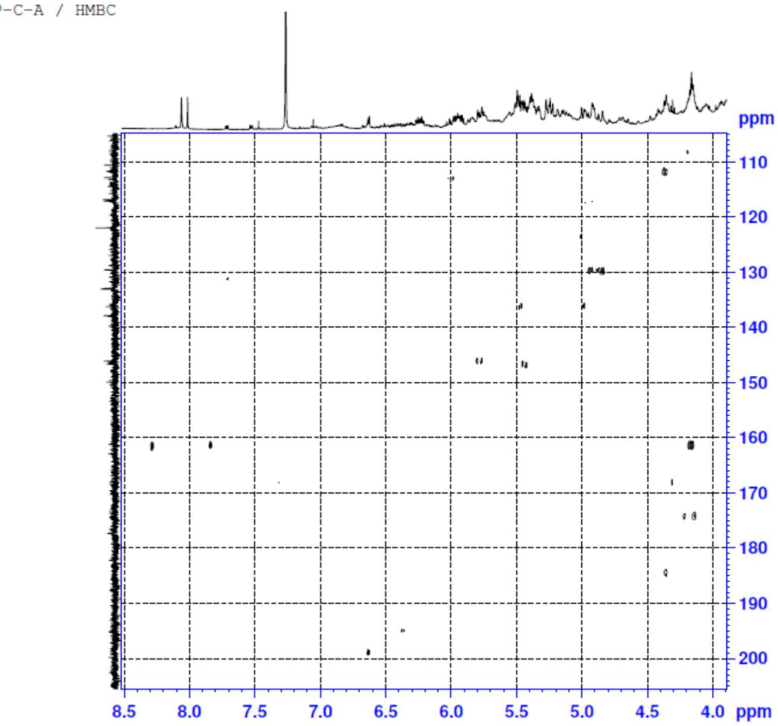

서 울 대 학 교
SEOUL NATIONAL UNIVERSITY
기초과학공동기기원
핵자기공명연구실

```
Current Data Parameters
NAME          jan02-kku-jim
EXPNO                     2
PROCNO                    1

F2 - Acquisition Parameters
Date_              20140103
Time                  17.35
INSTRUM               spect
PROBHD   5 mm Multinucl
PULPROG       hmbcgplpndqf
TD                     4096
SOLVENT               CDCl3
NS                      128
DS                       16
SWH            5647.590 Hz
FIDRES         1.378806 Hz
AQ            0.3626325 sec
RG                     2050
DW              88.533 usec
DE                6.50 usec
TE                  298.0 K
CNST2       145.0000000
CNST13       10.0000000
D0         0.00000300 sec
D1         1.50000000 sec
D2         0.00344828 sec
D6         0.05000000 sec
D16        0.00020000 sec
IN0        0.00001730 sec

======== CHANNEL f1 ========
SFO1        500.1325007 MHz
NUC1                     1H
P1               10.80 usec
P2               21.60 usec
PLW1          7.00000000 W

======== CHANNEL f2 ========
SFO2        125.7709936 MHz
NUC2                    13C
P3               12.00 usec
PLW2        180.00000000 W

====== GRADIENT CHANNEL =====
GPNAM[1]        SMSQ10.100
GPNAM[2]        SMSQ10.100
GPNAM[3]        SMSQ10.100
GPZ1                50.00 %
GPZ2                30.00 %
GPZ3                40.10 %
P16            1000.00 usec

F1 - Acquisition parameters
TD                      256
SFO1            125.771 MHz
FIDRES        112.897400 Hz
SW              229.796 ppm
FnMODE                   QF

F2 - Processing parameters
SI                     2048
SF          500.1300095 MHz
WDW                    SINE
SSB      0
LB       0 Hz
GB       0
PC                     1.40

F1 - Processing parameters
SI                     1024
MC2                      QF
SF          125.7577679 MHz
WDW                    SINE
SSB      0
LB       0 Hz
GB       0
```

DP-C-A / HMBC

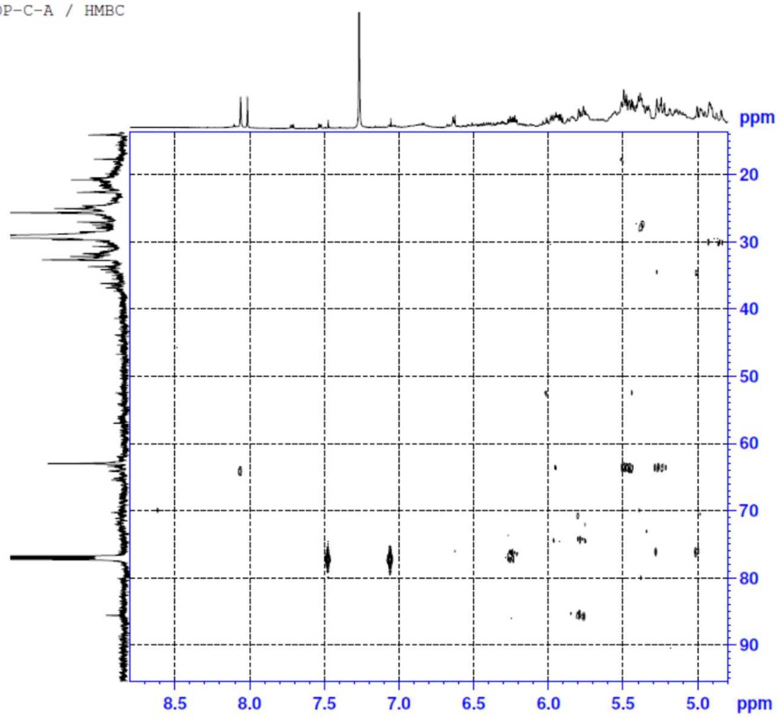

서 울 대 학 교
SEOUL NATIONAL UNIVERSITY
기초과학공동기기원
핵자기공명연구실

```
Current Data Parameters
NAME          jan02-kku-jim
EXPNO                     2
PROCNO                    1

F2 - Acquisition Parameters
Date_              20140103
Time                  17.35
INSTRUM               spect
PROBHD   5 mm Multinucl
PULPROG       hmbcgplpndqf
TD                     4096
SOLVENT               CDCl3
NS                      128
DS                       16
SWH            5647.590 Hz
FIDRES         1.378806 Hz
AQ            0.3626325 sec
RG                     2050
DW              88.533 usec
DE                6.50 usec
TE                  298.0 K
CNST2       145.0000000
CNST13       10.0000000
D0         0.00000300 sec
D1         1.50000000 sec
D2         0.00344828 sec
D6         0.05000000 sec
D16        0.00020000 sec
IN0        0.00001730 sec

======== CHANNEL f1 ========
SFO1        500.1325007 MHz
NUC1                     1H
P1               10.80 usec
P2               21.60 usec
PLW1          7.00000000 W

======== CHANNEL f2 ========
SFO2        125.7709936 MHz
NUC2                    13C
P3               12.00 usec
PLW2        180.00000000 W

====== GRADIENT CHANNEL =====
GPNAM[1]        SMSQ10.100
GPNAM[2]        SMSQ10.100
GPNAM[3]        SMSQ10.100
GPZ1                50.00 %
GPZ2                30.00 %
GPZ3                40.10 %
P16            1000.00 usec

F1 - Acquisition parameters
TD                      256
SFO1            125.771 MHz
FIDRES        112.897400 Hz
SW              229.796 ppm
FnMODE                   QF

F2 - Processing parameters
SI                     2048
SF          500.1300095 MHz
WDW                    SINE
SSB      0
LB       0 Hz
GB       0
PC                     1.40

F1 - Processing parameters
SI                     1024
MC2                      QF
SF          125.7577679 MHz
WDW                    SINE
SSB      0
LB       0 Hz
GB       0
```

DP-C-A / HMBC

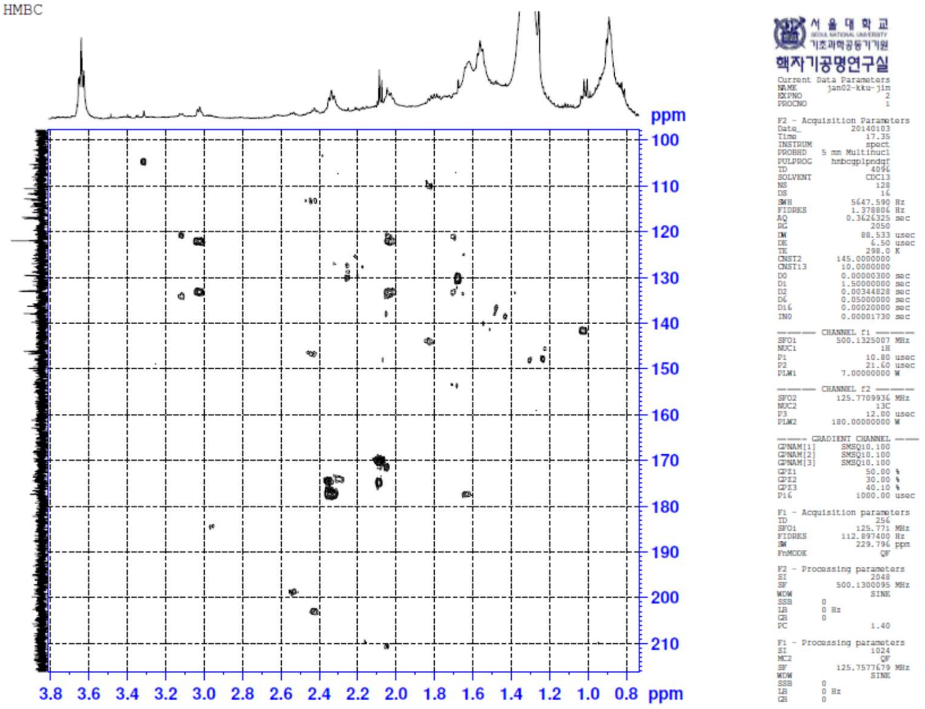

DP-C-A / HMBC

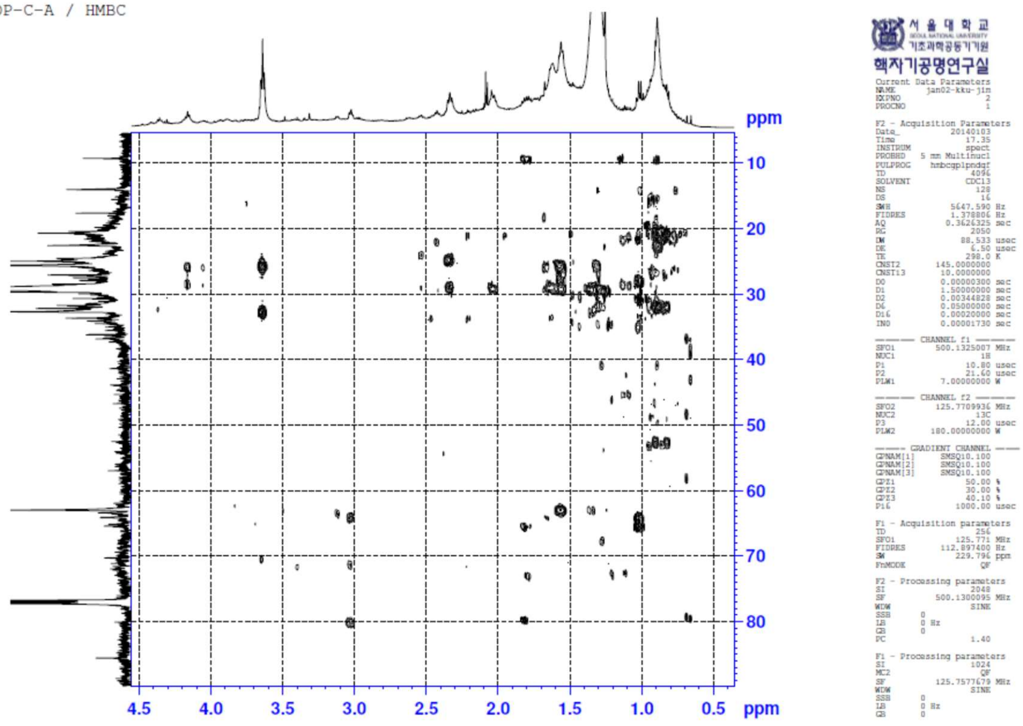

[Mass Spectrum]
Data : FAB-H100 Date : 16-Dec-2013 16:57
Sample: DP-C-A
Note : m-NBA
Inlet : Direct Ion Mode : FAB+
Spectrum Type : Normal Ion [MF-Linear]
RT : 0.00 min Scan# : (1,2)
BP : m/z 154.0000 Int. : 134.14
Output m/z range : 10.0000 to 550.2012 Cut Level : 0.00 %

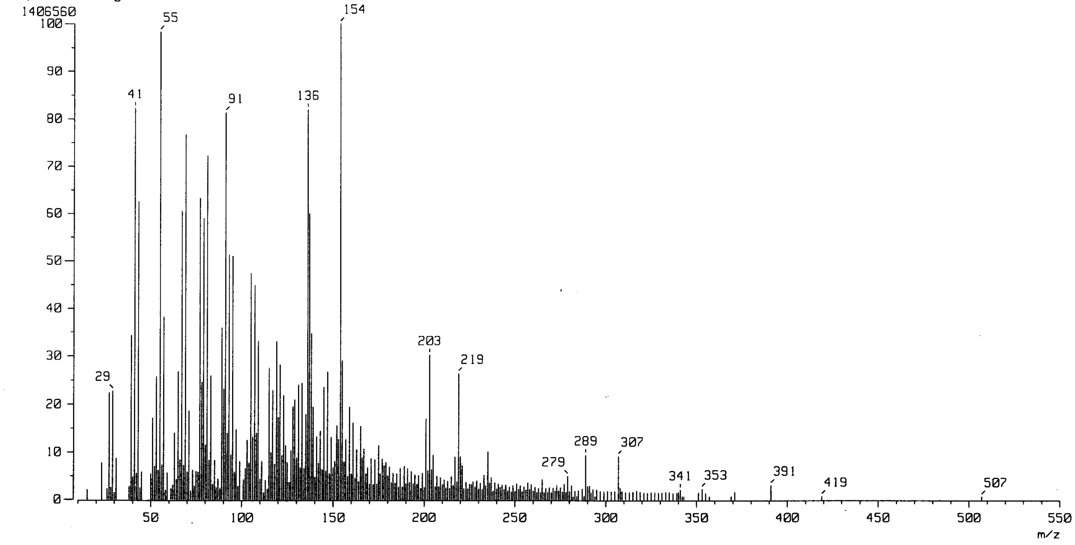

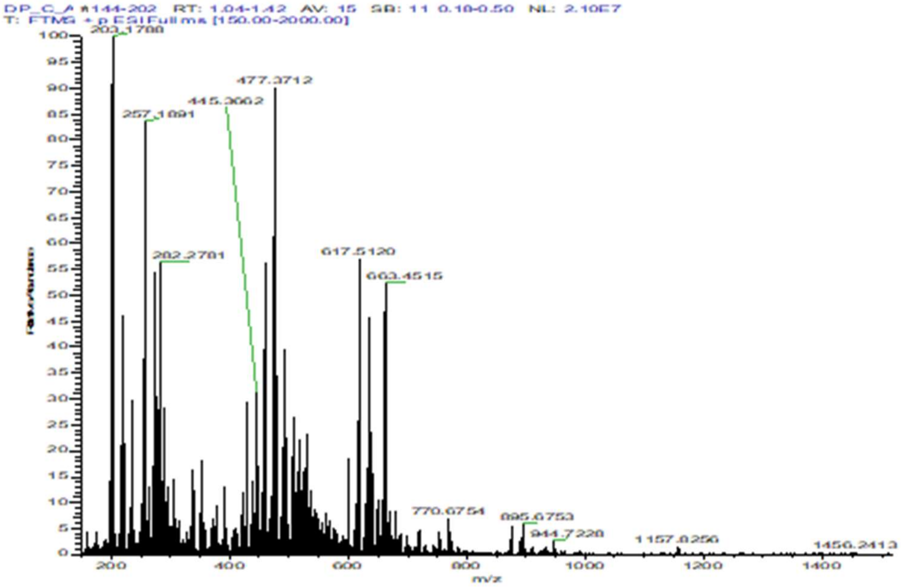

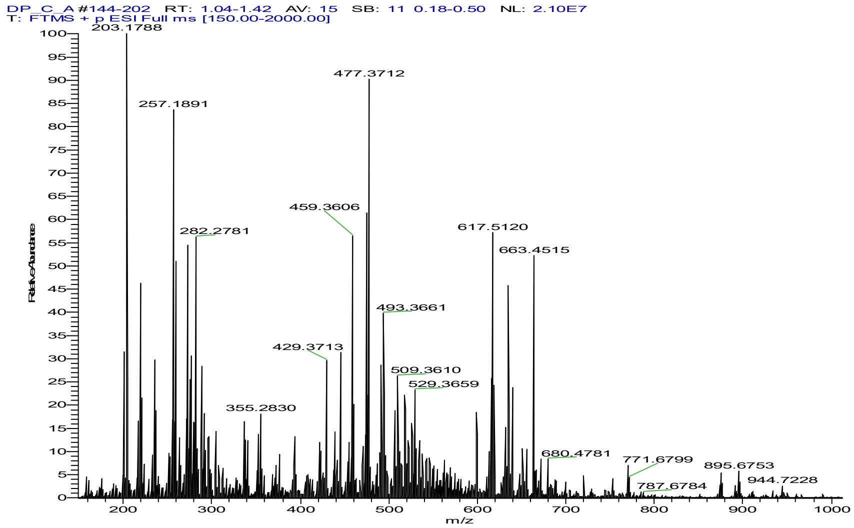
DP_C_A #144-202 RT: 1.04-1.42 AV: 15 SB: 11 0.18-0.50 NL: 2.10E7
T: FTMS + p ESI Full ms [150.00-2000.00]
203.1788
477.3712
257.1891
459.3606
282.2781
617.5120
663.4515
493.3661
429.3713
509.3610
529.3659
355.2830
680.4781
771.6799
895.6753
944.7228
787.6784
Relative Abundance
m/z

Compound 4

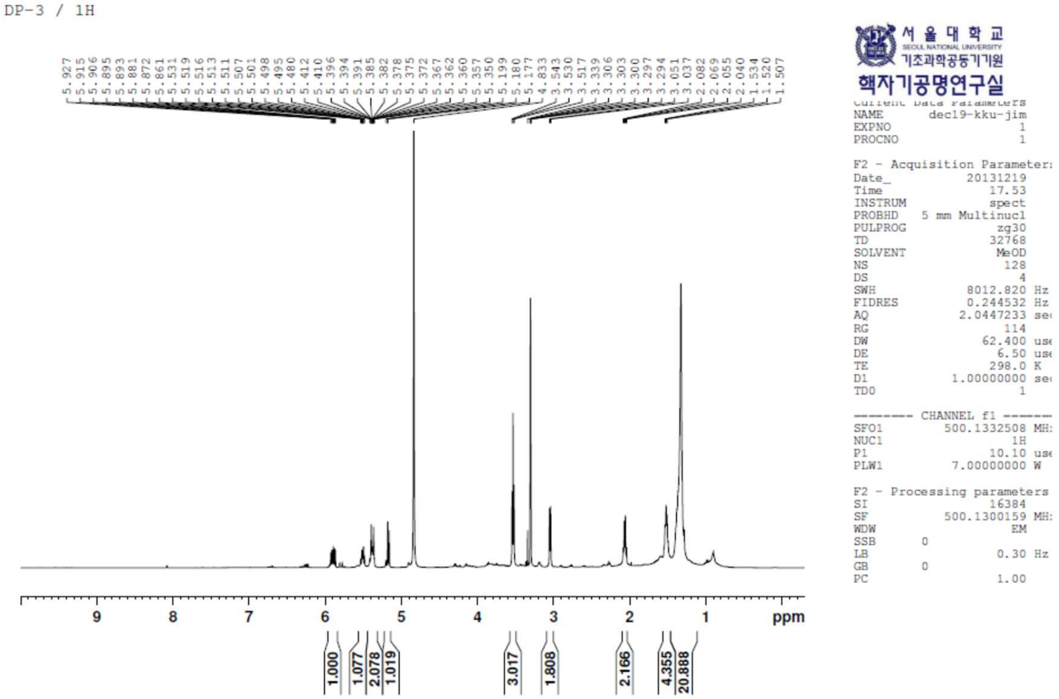
DP-3 / 1H
5.927
5.915
5.906
5.895
5.893
5.881
5.872
5.861
5.531
5.519
5.516
5.513
5.511
5.507
5.501
5.498
5.495
5.480
5.412
5.410
5.396
5.394
5.391
5.385
5.382
5.378
5.375
5.372
5.367
5.362
5.360
5.357
5.350
5.199
5.180
5.177
4.833
3.543
3.530
3.517
3.339
3.306
3.303
3.300
3.297
3.294
3.051
3.037
2.082
2.069
2.055
2.040
1.534
1.520
1.507
서울대학교
SEOUL NATIONAL UNIVERSITY
기초과학공동기기원
핵자기공명연구실
Current Data Parameters
NAME dec19-kku-jim
EXPNO 1
PROCNO 1
F2 - Acquisition Parameters
Date_ 20131219
Time 17.53
INSTRUM spect
PROBHD 5 mm Multinucl
PULPROG zg30
TD 32768
SOLVENT MeOD
NS 128
DS 4
SWH 8012.820 Hz
FIDRES 0.244532 Hz
AQ 2.0447233 sec
RG 114
DW 62.400 usec
DE 6.50 usec
TE 298.0 K
D1 1.00000000 sec
TD0 1
======== CHANNEL f1 ========
SFO1 500.1332508 MHz
NUC1 1H
P1 10.10 usec
PLW1 7.00000000 W
F2 - Processing parameters
SI 16384
SF 500.1300159 MHz
WDW EM
SSB 0
LB 0.30 Hz
GB 0
PC 1.00
ppm
1.000
1.077
2.078
1.019
3.017
1.808
2.166
4.355
20.888

DP-3 / 1H

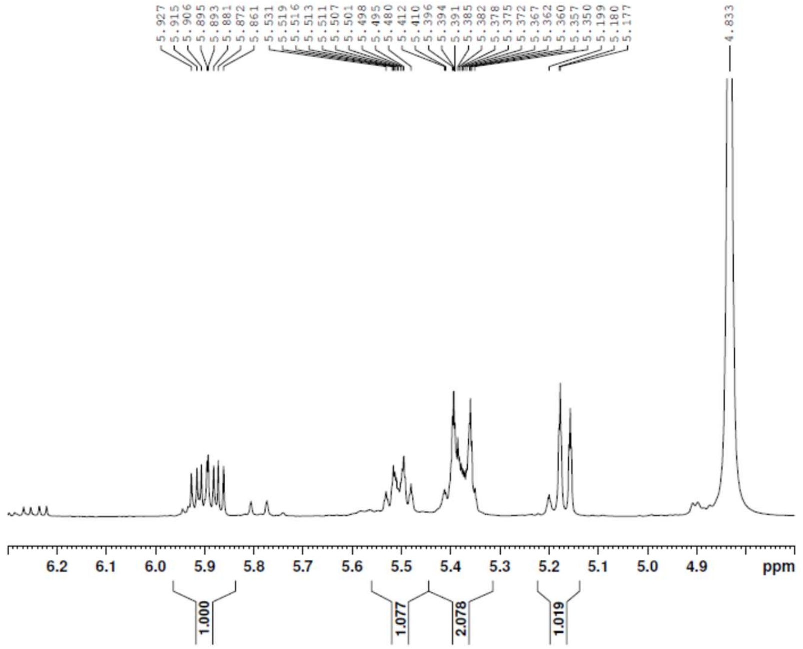

서 울 대 학 교
SEOUL NATIONAL UNIVERSITY
기초과학공동기기원
핵자기공명연구실

```
Current Data Parameters
NAME        dec19-kku-jim
EXPNO                   1
PROCNO                  1

F2 - Acquisition Parameter[illegible]
Date_            20131219
Time                17.53
INSTRUM             spect
PROBHD   5 mm Multinucl
PULPROG              zg30
TD                  32768
SOLVENT              MeOD
NS                    128
DS                      4
SWH             8012.820 Hz
FIDRES          0.244532 Hz
AQ             2.0447233 se[illegible]
RG                    114
DW                 62.400 us[illegible]
DE                   6.50 us[illegible]
TE                  298.0 K
D1            1.00000000 se[illegible]
TD0                     1

======= CHANNEL f1 ======
SFO1         500.1332508 MH[illegible]
NUC1                   1H
P1                  10.10 us[illegible]
PLW1          7.00000000 W

F2 - Processing parameters
SI                  16384
SF           500.1300159 MH[illegible]
WDW                    EM
SSB      0
LB                   0.30 Hz
GB       0
PC                   1.00
```

DP-3 / 1H

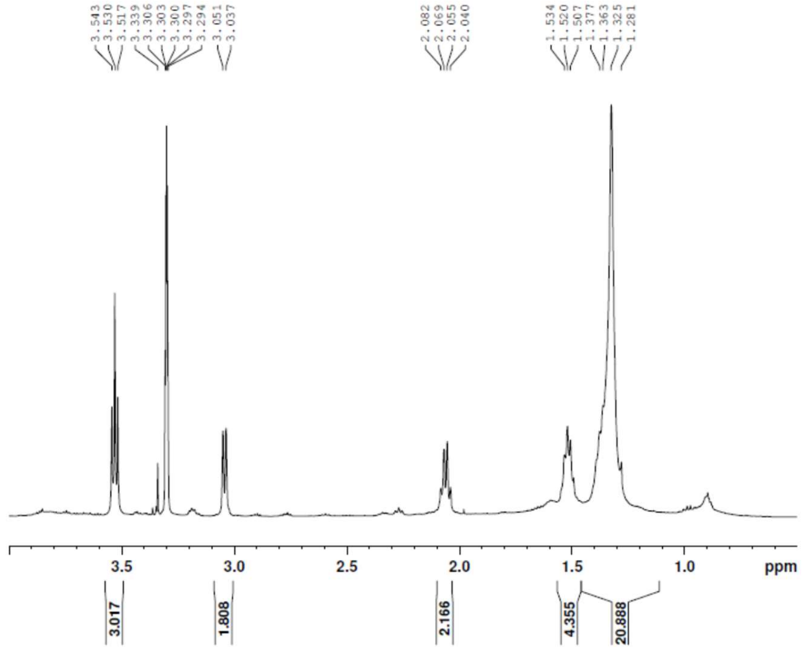

서 울 대 학 교
SEOUL NATIONAL UNIVERSITY
기초과학공동기기원
핵자기공명연구실

```
Current Data Parameters
NAME        dec19-kku-jim
EXPNO                   1
PROCNO                  1

F2 - Acquisition Parameter[illegible]
Date_            20131219
Time                17.53
INSTRUM             spect
PROBHD   5 mm Multinucl
PULPROG              zg30
TD                  32768
SOLVENT              MeOD
NS                    128
DS                      4
SWH             8012.820 Hz
FIDRES          0.244532 Hz
AQ             2.0447233 se[illegible]
RG                    114
DW                 62.400 us[illegible]
DE                   6.50 us[illegible]
TE                  298.0 K
D1            1.00000000 se[illegible]
TD0                     1

======= CHANNEL f1 ======
SFO1         500.1332508 MH[illegible]
NUC1                   1H
P1                  10.10 us[illegible]
PLW1          7.00000000 W

F2 - Processing parameters
SI                  16384
SF           500.1300159 MH[illegible]
WDW                    EM
SSB      0
LB                   0.30 Hz
GB       0
PC                   1.00
```

DP-3 / 13C

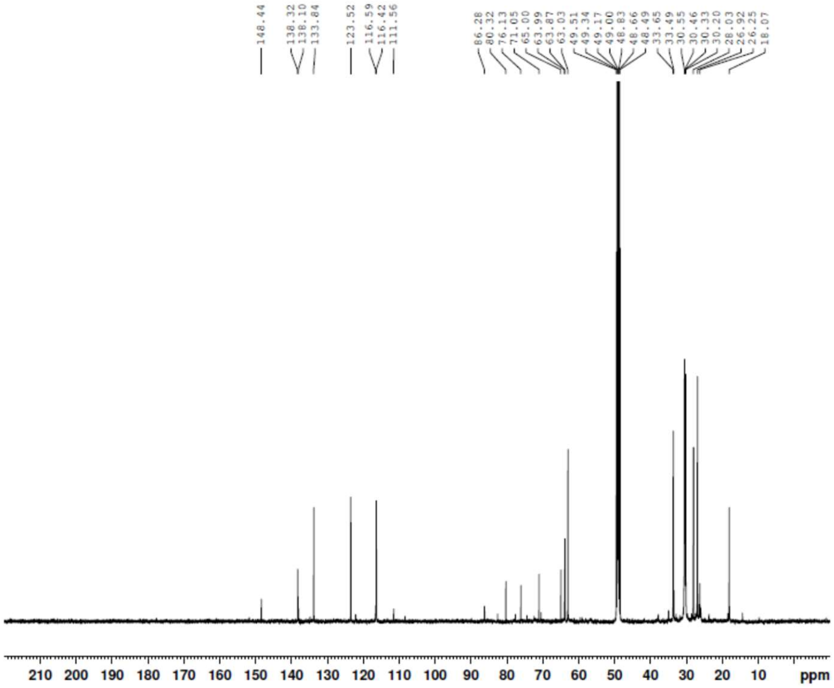

Current Data Parameters
NAME dec19-kku-jim
EXPNO 5
PROCNO 1

F2 - Acquisition Parameters
Date_ 20131221
Time 23.42
INSTRUM spect
PROBHD 5 mm Multinucl
PULPROG zgdc
TD 32768
SOLVENT MeOD
NS 35840
DS 4
SWH 29761.904 Hz
FIDRES 0.908261 Hz
AQ 0.5505024 sec
RG 912
DW 16.800 usec
DE 6.50 usec
TE 298.0 K
D1 2.00000000 sec
D11 0.03000000 sec
TD0 1

======== CHANNEL f1 ========
SFO1 125.7709936 MHz
NUC1 13C
P1 12.00 usec
PLW1 180.00000000 W

======== CHANNEL f2 ========
SFO2 500.1320005 MHz
NUC2 1H
CPDPRG[2 waltz16
PCPD2 80.00 usec
PLW2 8.00000000 W
PLW12 0.35066000 W

F2 - Processing parameters
SI 16384
SF 125.7576129 MHz
WDW EM
SSB 0
LB 1.00 Hz
GB 0
PC 1.40

DP-3 / 13C

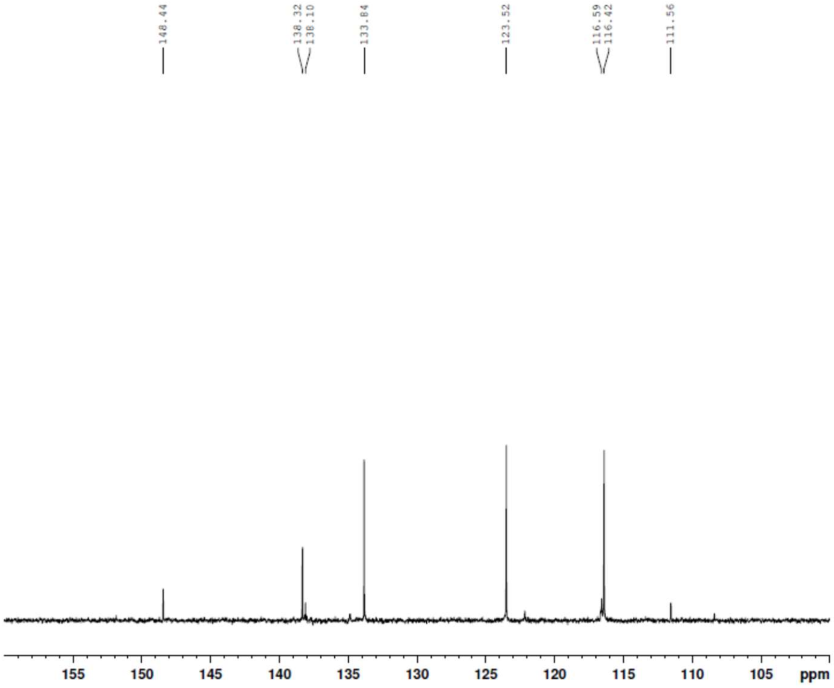

Current Data Parameters
NAME dec19-kku-jim
EXPNO 5
PROCNO 1

F2 - Acquisition Parameters
Date_ 20131221
Time 23.42
INSTRUM spect
PROBHD 5 mm Multinucl
PULPROG zgdc
TD 32768
SOLVENT MeOD
NS 35840
DS 4
SWH 29761.904 Hz
FIDRES 0.908261 Hz
AQ 0.5505024 sec
RG 912
DW 16.800 usec
DE 6.50 usec
TE 298.0 K
D1 2.00000000 sec
D11 0.03000000 sec
TD0 1

======== CHANNEL f1 ========
SFO1 125.7709936 MHz
NUC1 13C
P1 12.00 usec
PLW1 180.00000000 W

======== CHANNEL f2 ========
SFO2 500.1320005 MHz
NUC2 1H
CPDPRG[2 waltz16
PCPD2 80.00 usec
PLW2 8.00000000 W
PLW12 0.35066000 W

F2 - Processing parameters
SI 16384
SF 125.7576129 MHz
WDW EM
SSB 0
LB 1.00 Hz
GB 0
PC 1.40

DP-3 / 13C

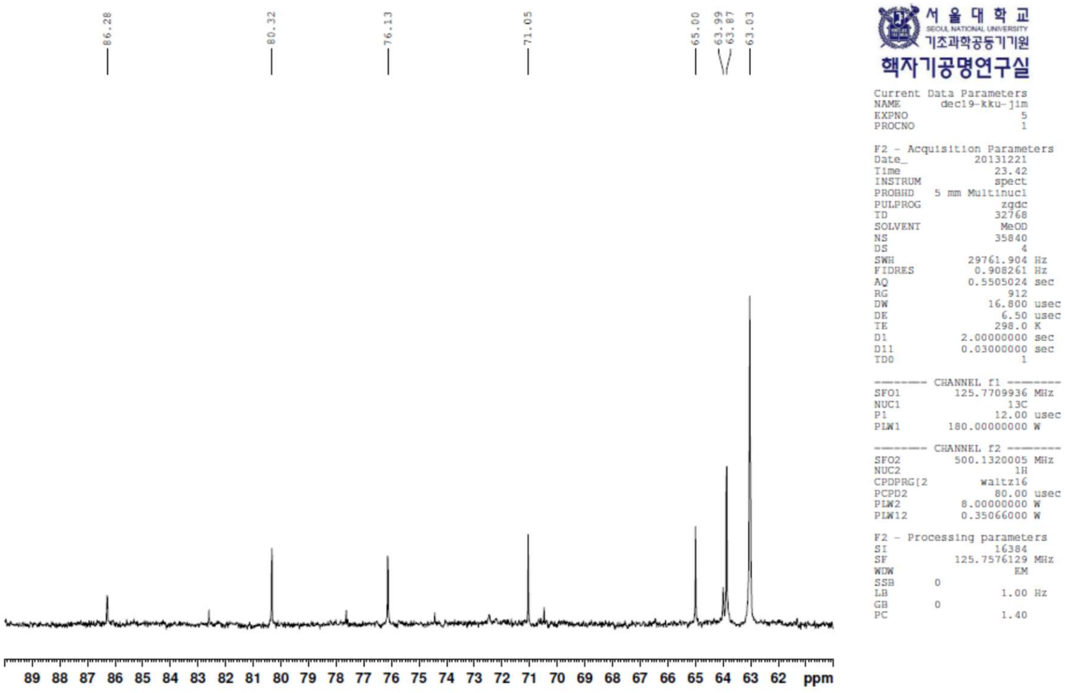

DP-3 / 13C

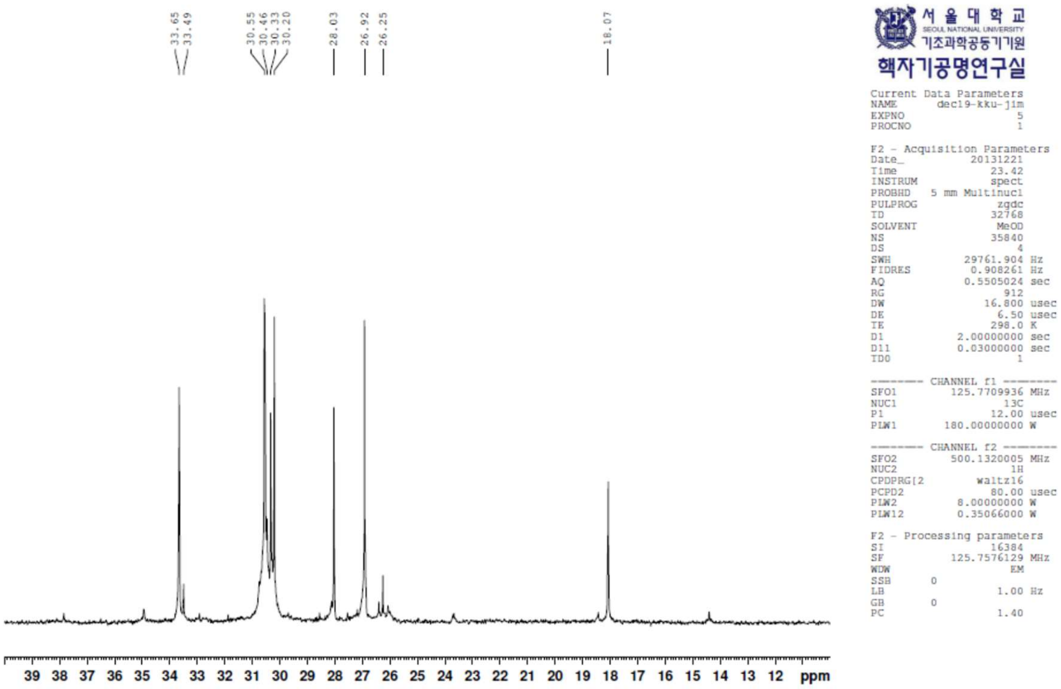

DP-3 / HMBC

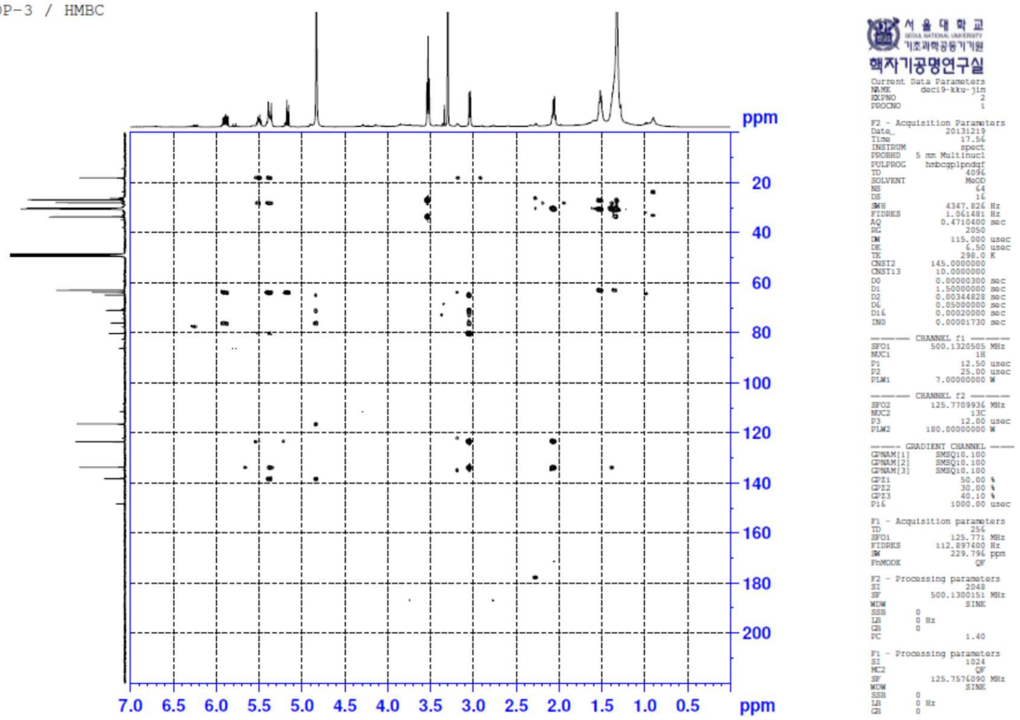

DP-3 / HMBC

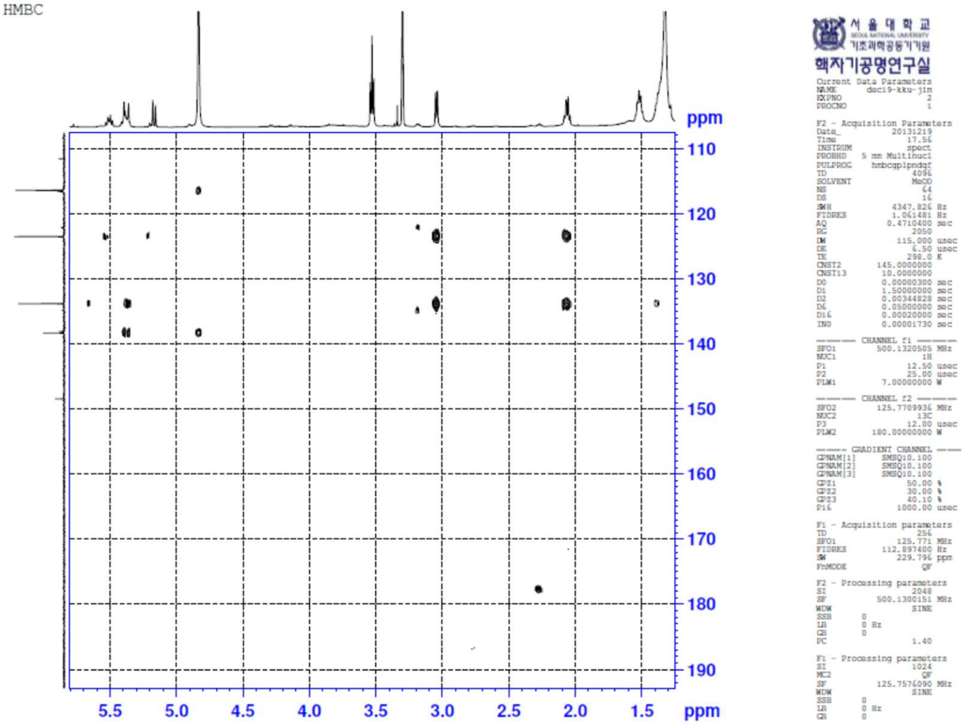

DP-3 / HMBC

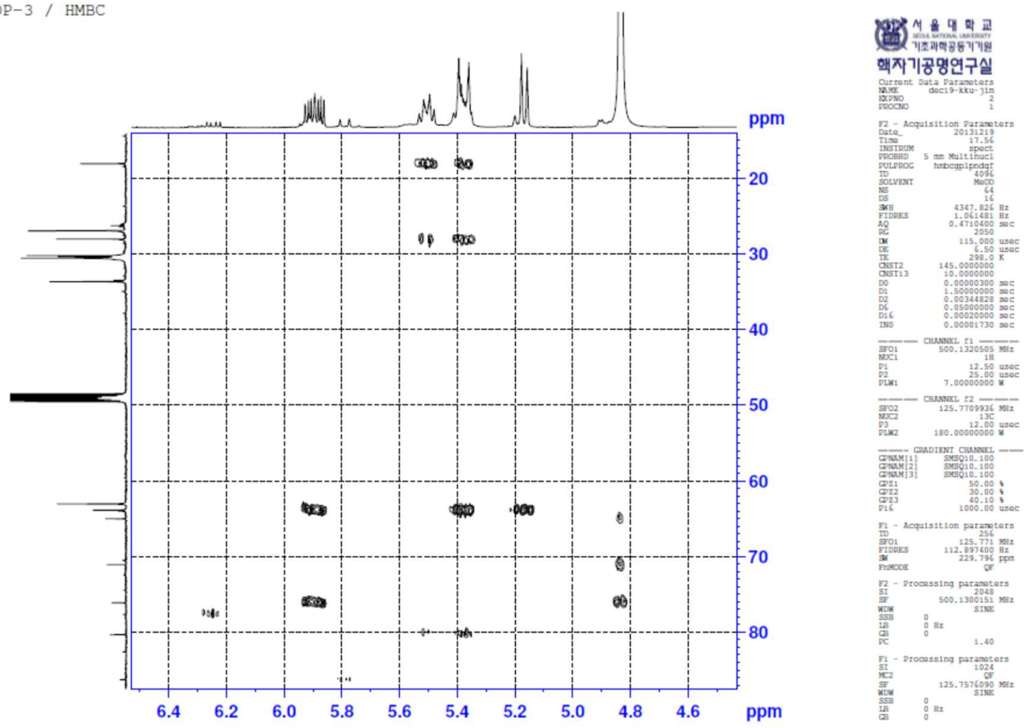

DP-3 / HMBC

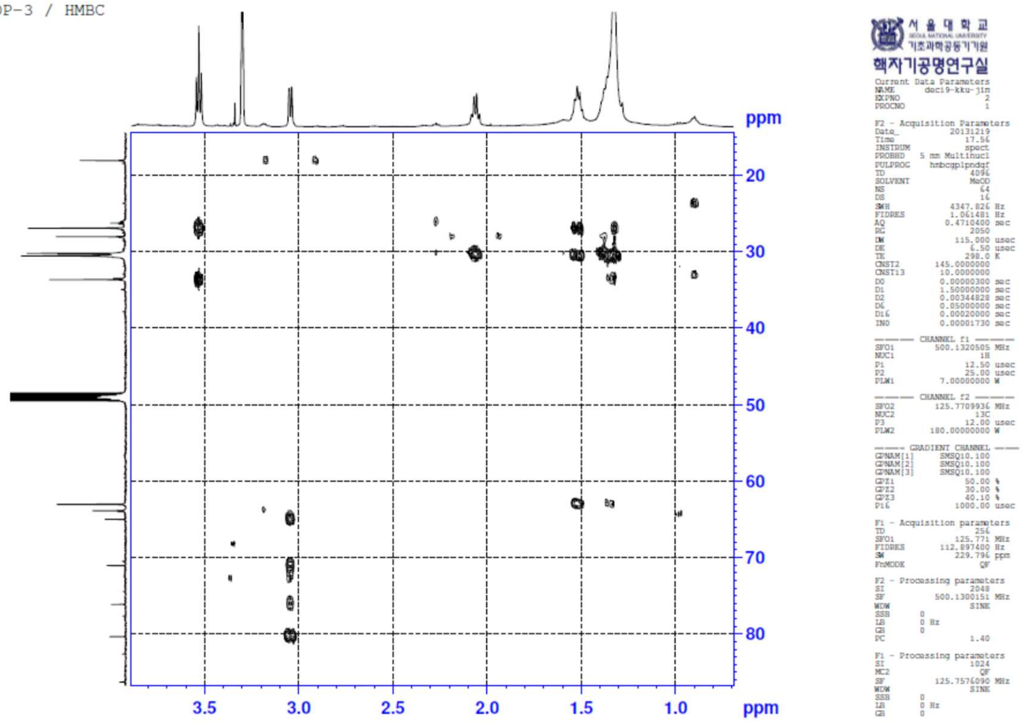

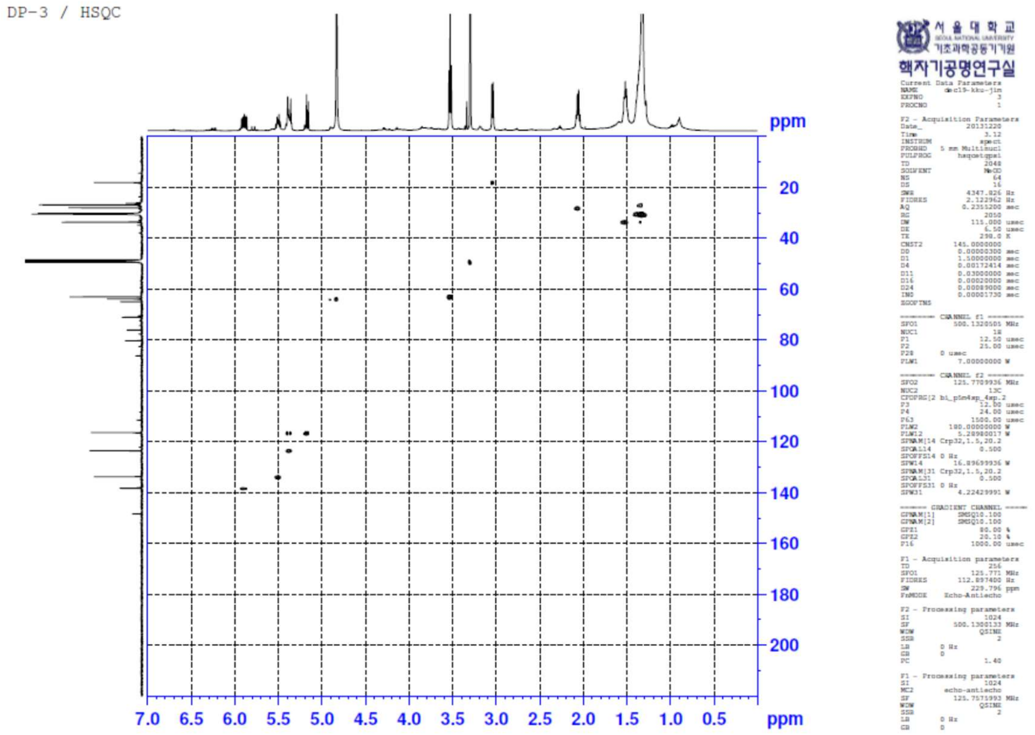
DP-3 / HSQC
서울대학교
기초과학공동기기원
핵자기공명연구실
ppm

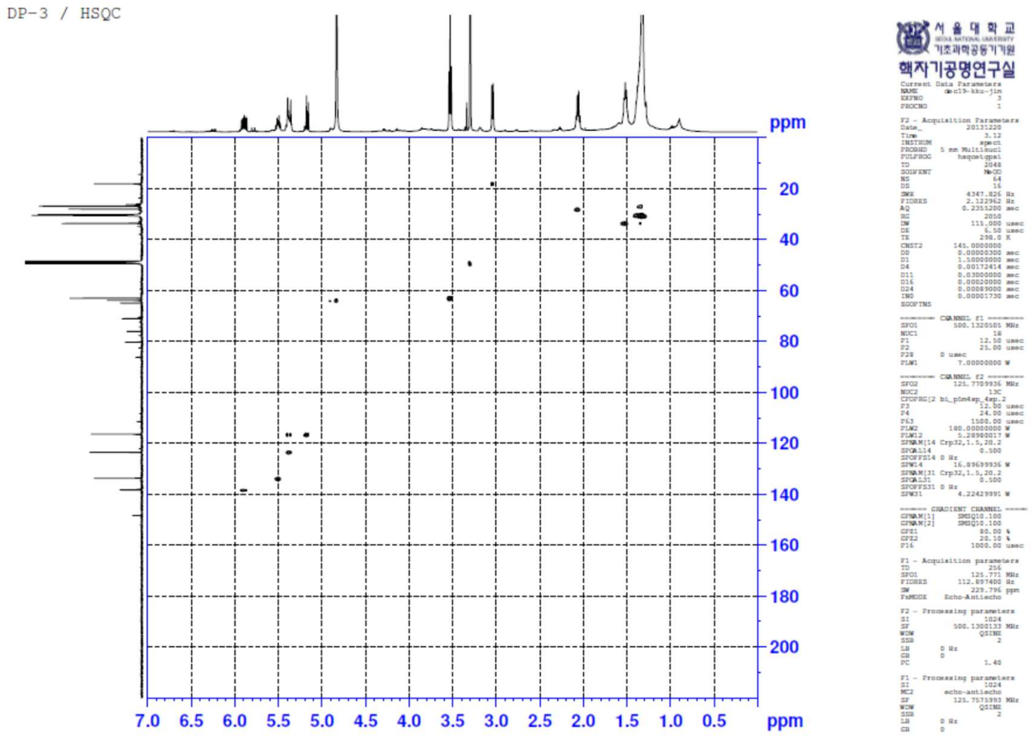
DP-3 / HSQC
서울대학교
기초과학공동기기원
핵자기공명연구실
ppm

DP-3 / HSQC

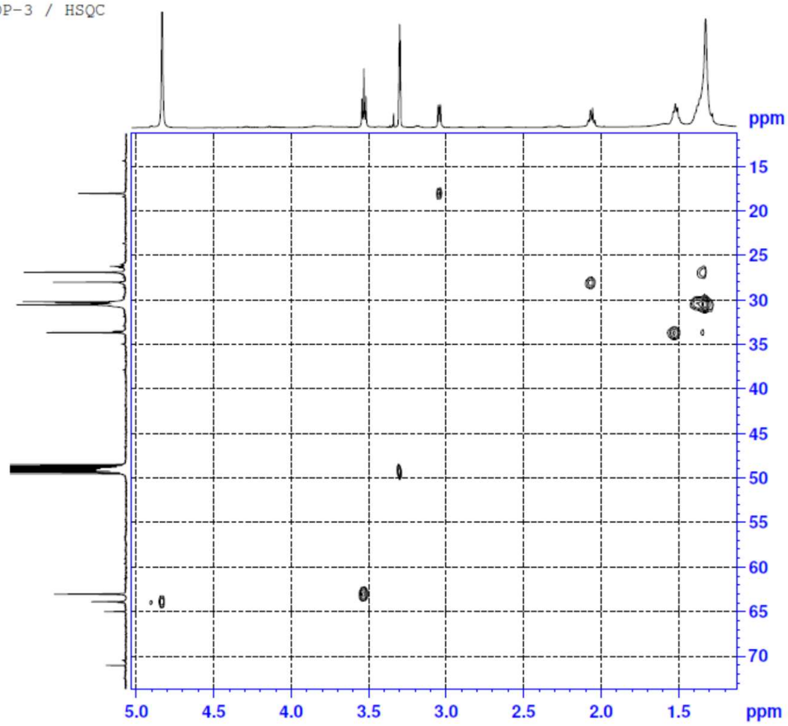

서 울 대 학 교
SEOUL NATIONAL UNIVERSITY
기초과학공동기기원
핵자기공명연구실

```
Current Data Parameters
NAME        dec19-kku-jim
EXPNO                   3
PROCNO                  1

F2 - Acquisition Parameters
Date_            20131220
Time                 3.12
INSTRUM             spect
PROBHD   5 mm Multinucl
PULPROG        hsqcetgpsi
TD                   2048
SOLVENT              MeOD
NS                     64
DS                     16
SWH              4347.826 Hz
FIDRES           2.122962 Hz
AQ              0.2355200 sec
RG                   2050
DW                115.000 usec
DE                   6.50 usec
TE                  298.0 K
CNST2         145.0000000
D0             0.00000300 sec
D1             1.50000000 sec
D4             0.00172414 sec
D11            0.03000000 sec
D16            0.00020000 sec
D24            0.00089000 sec
IN0            0.00001730 sec
ZGOPTNS

======== CHANNEL f1 ========
SFO1          500.1320505 MHz
NUC1                   1H
P1                  12.50 usec
P2                  25.00 usec
P28        0 usec
PLW1           7.00000000 W

======== CHANNEL f2 ========
SFO2          125.7709936 MHz
NUC2                  13C
CPDPRG[2 bi_p5m4sp_4sp.2
P3                  12.00 usec
P4                  24.00 usec
P63               1500.00 usec
PLW2         180.00000000 W
PLW12          5.28980017 W
SPNAM[14 Crp32,1.5,20.2
SPOAL14             0.500
SPOFFS14 0 Hz
SPW14         16.89699936 W
SPNAM[31 Crp32,1.5,20.2
SPOAL31             0.500
SPOFFS31 0 Hz
SPW31          4.22429991 W

====== GRADIENT CHANNEL =====
GPNAM[1]      SMSQ10.100
GPNAM[2]      SMSQ10.100
GPZ1                80.00 %
GPZ2                20.10 %
P16               1000.00 usec

F1 - Acquisition parameters
TD                    256
SFO1              125.771 MHz
FIDRES         112.897400 Hz
SW                229.796 ppm
FnMODE      Echo-Antiecho

F2 - Processing parameters
SI                   1024
SF            500.1300133 MHz
WDW                 QSINE
SSB                     2
LB         0 Hz
GB         0
PC                   1.40

F1 - Processing parameters
SI                   1024
MC2          echo-antiecho
SF            125.7571993 MHz
WDW                 QSINE
SSB                     2
LB         0 Hz
GB         0
```

DP-3 / COSY

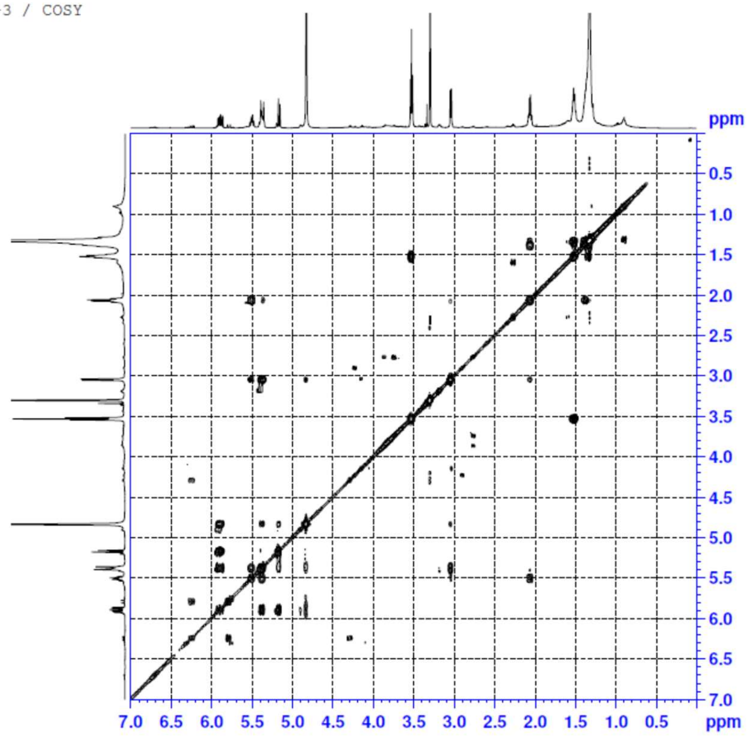

서 울 대 학 교
SEOUL NATIONAL UNIVERSITY
기초과학공동기기원
핵자기공명연구실

```
Current Data Parameters
NAME        dec19-kku-jim
EXPNO                   4
PROCNO                  1

F2 - Acquisition Parameters
Date_            20131220
Time                11.12
INSTRUM             spect
PROBHD   5 mm Multinucl
PULPROG         cosygpppqf
TD                   2048
SOLVENT              MeOD
NS                     64
DS                      8
SWH              4347.826 Hz
FIDRES           2.122962 Hz
AQ              0.2355200 sec
RG                   25.4
DW                115.000 usec
DE                   6.50 usec
TE                  298.0 K
D0             0.00000300 sec
D1             2.00000000 sec
D11            0.03000000 sec
D12            0.00002000 sec
D13            0.00000400 sec
D16            0.00020000 sec
IN0            0.00022980 sec

======== CHANNEL f1 ========
SFO1          500.1320505 MHz
NUC1                   1H
P0                  12.50 usec
P1                  12.50 usec
P17               2500.00 usec
PLW1           7.00000000 W
PLW10          1.05630004 W

====== GRADIENT CHANNEL =====
GPNAM[1]      SMSQ10.100
GPZ1                10.00 %
P16               1000.00 usec

F1 - Acquisition parameters
TD                    256
SFO1             500.1321 MHz
FIDRES          16.998476 Hz
SW                  8.701 ppm
FnMODE                 QF

F2 - Processing parameters
SI                   1024
SF            500.1300138 MHz
WDW                 QSINE
SSB        0
LB         0 Hz
GB         0
PC                   1.40

F1 - Processing parameters
SI                   1024
MC2                    QF
SF            500.1300144 MHz
WDW                 QSINE
SSB        0
LB         0 Hz
GB         0
```

DP-3 / COSY

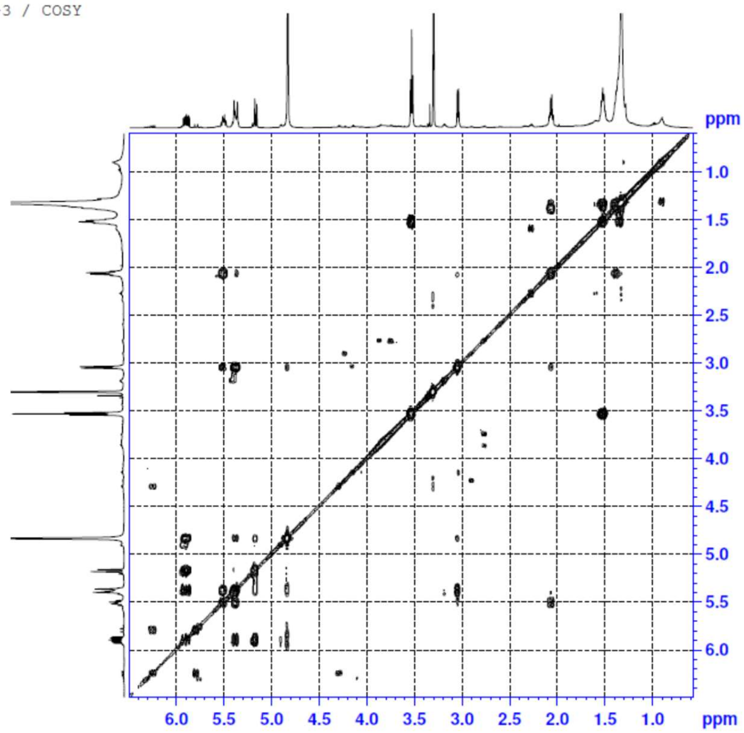

서울대학교
SEOUL NATIONAL UNIVERSITY
기초과학공동기기원
핵자기공명연구실

```
Current Data Parameters
NAME          dec19-kku-jim
EXPNO                     4
PROCNO                    1

F2 - Acquisition Parameters
Date_              20131220
Time                  11.12
INSTRUM               spect
PROBHD    5 mm Multinucl
PULPROG          cosygpppqf
TD                     2048
SOLVENT                MeOD
NS                       64
DS                        8
SWH                4347.826 Hz
FIDRES             2.122962 Hz
AQ                0.2355200 sec
RG                     25.4
DW                  115.000 usec
DE                     6.50 usec
TE                    298.0 K
D0               0.00000300 sec
D1               2.00000000 sec
D11              0.03000000 sec
D12              0.00002000 sec
D13              0.00000400 sec
D16              0.00020000 sec
IN0              0.00022980 sec

======== CHANNEL f1 ========
SFO1            500.1320505 MHz
NUC1                     1H
P0                    12.50 usec
P1                    12.50 usec
P17                 2500.00 usec
PLW1             7.00000000 W
PLW10            1.05630004 W

====== GRADIENT CHANNEL =====
GPNAM[1]          SMSQ10.100
GPZ1                  10.00 %
P16                 1000.00 usec

F1 - Acquisition parameters
TD                      256
SFO1               500.1321 MHz
FIDRES            16.998476 Hz
SW                    8.701 ppm
FnMODE                   QF

F2 - Processing parameters
SI                     1024
SF              500.1300138 MHz
WDW                   QSINE
SSB        0
LB         0 Hz
GB         0
PC                     1.40

F1 - Processing parameters
SI                     1024
MC2                      QF
SF              500.1300144 MHz
WDW                   QSINE
SSB        0
LB         0 Hz
GB         0
```

[Mass Spectrum]
Data : FAB-H119 Date : 17-Dec-2013 15:46
Sample: DP-3
Note : m-NBA
Inlet : Direct Ion Mode : FAB+
Spectrum Type : Normal Ion [MF-Linear]
RT : 0.21 min Scan# : (1,3)
BP : m/z 55.0000 Int. : 170.82
Output m/z range : 10.0000 to 701.4688 Cut Level : 0.00 %

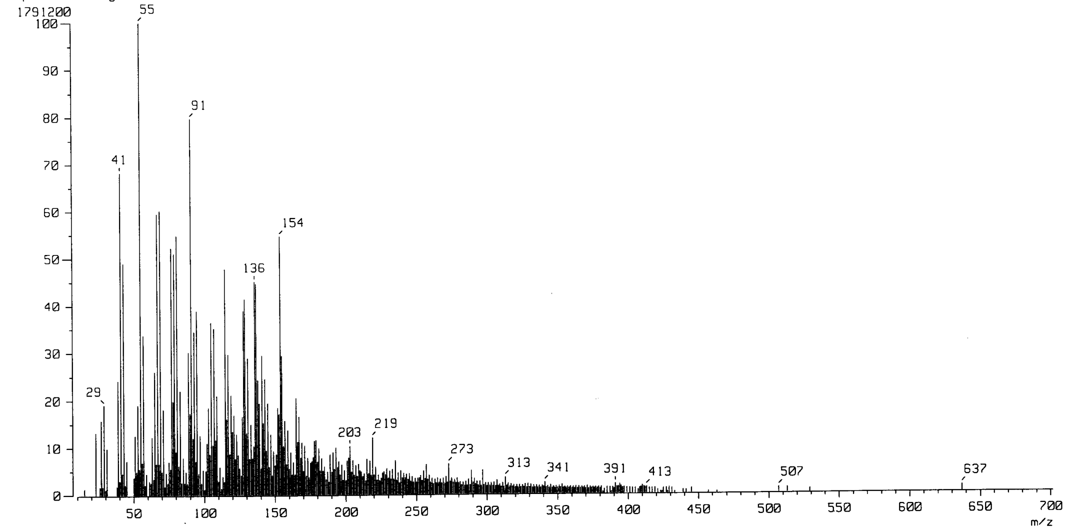

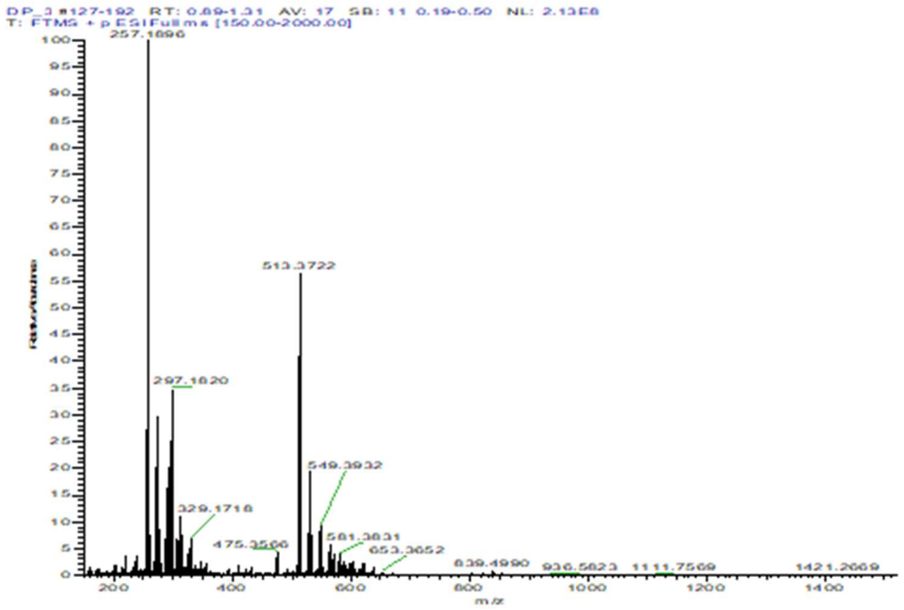
DP_3 #127-192 RT: 0.89-1.31 AV: 17 SB: 11 0.19-0.50 NL: 2.13E8
T: FTMS + p ESI Full ms [150.00-2000.00]
257.1896
513.3722
297.1820
549.3932
329.1718
475.3566
581.3831
653.3652
839.4990
936.5823
1111.7569
1421.2669
m/z

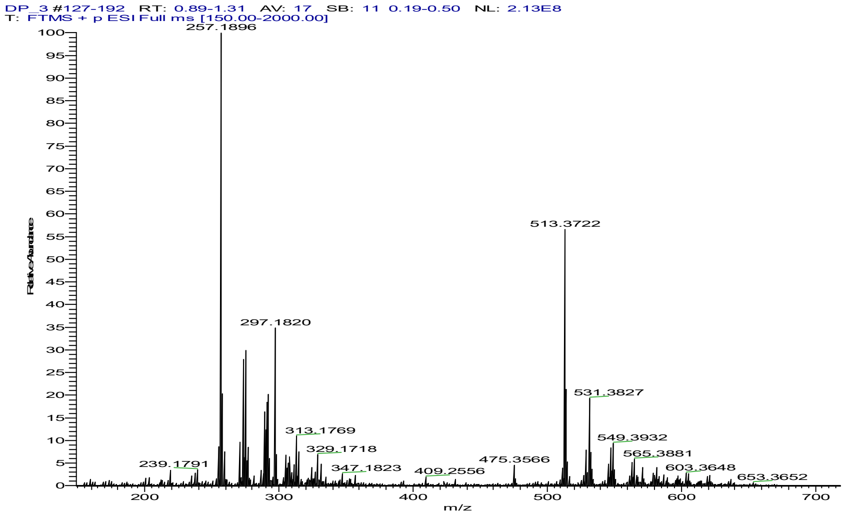
DP_3 #127-192 RT: 0.89-1.31 AV: 17 SB: 11 0.19-0.50 NL: 2.13E8
T: FTMS + p ESI Full ms [150.00-2000.00]
257.1896
513.3722
297.1820
531.3827
313.1769
549.3932
329.1718
565.3881
239.1791
347.1823
409.2556
475.3566
603.3648
653.3652
m/z
